# A network meta-analysis of efficacy and safety for first-line and second/further-line therapies in postmenopausal women with hormone receptor-positive, HER2-negative, advanced breast cancer

**DOI:** 10.1186/s12916-023-03238-2

**Published:** 2024-01-12

**Authors:** Hanqiao Shao, Mingye Zhao, Ai-Jia Guan, Taihang Shao, Dachuang Zhou, Guo Yu, Wenxi Tang

**Affiliations:** 1 School of International Pharmaceutical Business, China Pharmaceutical University, Nanjing, Jiangsu China; 2 Center for Pharmacoeconomics and Outcomes Research & Department of Public Affairs Management, School of International Pharmaceutical Business, China Pharmaceutical University, Nanjing, Jiangsu China; 3 Department of Rehabilitation Medicine, West China Hospital, Sichuan University, Sichuan, China; 4 School of Basic Medicine and Clinical Pharmacy, China Pharmaceutical University, Nanjing, Jiangsu China

**Keywords:** HR-positive/HER2-negative, Advanced breast cancer, Efficacy, Safety, Network meta-analysis

## Abstract

**Background:**

Hormone receptor-positive/human epidermal growth factor receptor 2-negative (HR + /HER2 −) advanced breast cancer is a prevalent subtype among postmenopausal women. Despite the growing number of randomized clinical trials (RCTs) exploring this topic, the efficacy and safety of first-line and second/further-line treatments remain uncertain. Accordingly, our aim was to conduct a comprehensive evaluation of the efficacy and safety of these therapies through network meta-analysis.

**Methods:**

RCTs were identified by searching Pubmed, Embase, and major cancer conferences. The efficacy of interventions was assessed using the hazard ratios (HRs) of progression-free survival (PFS) and overall survival (OS), while safety was indicated by the incidence of any grade adverse events (AEs), grade 3–5 AEs, AEs leading to treatment discontinuation, and AEs leading to death. Both time-variant HRs fractional polynomial models and time-invariant HRs Cox-proportional hazards models were considered for handling time-to-event data. Safety indicators were analyzed using Bayesian network meta-analysis. Additionally, subgroup analyses were conducted based on patient characteristics.

**Results:**

A total of 41 RCTs (first-line 17, second/further-lines 27) were included in the analysis. For first-line treatment, the addition of Cyclin-dependent kinase 4 and 6 (CDK4/6) inhibitors to endocrine therapy significantly improved therapeutic efficacy in terms of both PFS and OS, demonstrating the best performance across all mechanisms. Specifically, the combination of Abemaciclib and Letrozole demonstrated the most favorable performance in terms of PFS, while Ribociclib plus Fulvestrant yielded the best outcomes in OS. Incorporating the immune checkpoint inhibitor Avelumab into the regimen with CDK4/6 inhibitors and selective estrogen receptor degraders significantly enhanced both PFS and OS in second-line or later treatments. Regarding safety, endocrine monotherapy performed well. Regarding safety, endocrine monotherapy performed well. There is mounting evidence suggesting that most CDK4/6 inhibitors may demonstrate poorer performance with respect to hematologic AEs. However, additional evidence is required to further substantiate these findings.

**Conclusions:**

CDK4/6 inhibitors, combined with endocrine therapy, are pivotal in first-line treatment due to their superior efficacy and manageable AEs. For second/further-line treatment, adding immune checkpoint inhibitors to CDK4/6 inhibitors plus endocrine therapy may produce promising results. However, to reduce the results’ uncertainty, further trials comparing these novel treatments are warranted.

**Trial registration:**

Registration number: PROSPERO (CRD42022377431).

**Supplementary Information:**

The online version contains supplementary material available at 10.1186/s12916-023-03238-2.

## Background

Breast cancer is a prevalent malignancy and the primary cause of cancer-related death among women globally. It constitutes 15.5% of all female cancer fatalities [1], with around 2.3 million new breast cancer cases recorded worldwide in 2020 and approximately 685,000 deaths [2]. According to the Surveillance, Epidemiology, and End Results database of the National Cancer Institute in the USA, it is estimated that there will be about 297,790 new cases of breast cancer and 43,170 breast cancer-related deaths in 2023. These account for 15.2% of all new cancer cases and 7.1% of all cancer-related deaths, respectively [3]. Most breast cancer cases occur in females (approximately 99%), but there are approximately 2500 new cases of breast cancer diagnosed in males annually in the USA [4]. In clinical practice, breast cancer is commonly subtyped by hormone receptor (HR) and human epidermal growth factor receptor 2 (HER2). The subtype of HR-positive/HER2-negative represents the most prevalent among all racial groups, accounting for approximately 68% of cases across all ethnicities [5]. Historically, the estrogen receptor signaling pathway primarily drives cancer cell growth and survival in these tumors, these tumors were treated with endocrine therapy (ET) [6, 7]. However, due to limitations in single-agent endocrine therapy efficacy and the presence of primary or secondary drug resistance, endocrine monotherapy is increasingly insufficient for clinical treatment. The development of targeted therapies including Cyclin-dependent kinase 4 and 6 inhibitors (CDK4/6i) and immune checkpoint inhibitor (ICI), particularly in combination with ET, has revolutionized the management of these tumors [8]. Nonetheless, the comparative efficacy and safety of most first-line and second/further-line treatments remain uncertain. While some randomized controlled trials (RCTs) have verified the safety and efficacy of targeted therapies or combined targeted endocrine therapy for patients with HR + /HER2 − advanced breast cancer, the absence of head-to-head comparisons prevents us from determining which treatment offers the greatest survival advantage.

Furthermore, existing network meta-analyses (NMA) have only evaluated a limited range of treatment options for efficacy and safety [9–12], without delving into the mechanisms of targeted therapies [13]. Notably, nearly all these studies were executed using the proportional hazards (PH) model, without verifying the PH assumption. Nonetheless, it is important to note that the hazard ratios (HRs) represent the efficacy ratio within a specific time frame between treatments, calculated using a semi-parametric Cox-proportional hazards (Cox-PH) model [14]. In most studies, the PH assumption was not valid, indicating that the relative efficacy between treatments varied over time. This potential limitation may compromise the reliability and accuracy of the results [15]. Consequently, our study aimed to compare the efficacy and safety of first-line and second/further-line therapies in treating postmenopausal female patients with HR + /HER2 − advanced breast cancer, using an adjusted indirect comparison under a Bayesian framework that assumes non-PH.

## Methods

### Protocol

This study was conducted according to the Preferred Reporting Items for Systematic Reviews and Meta-Analyses extension statement for network meta-analyses of healthcare interventions [16] (Additional file 1: Table S1). The research plan for this project has been registered in PROSPERO (CRD42022377431).

### Data sources

A systematic search was conducted across various databases and websites such as PubMed, Embase, the European Society for Medical Oncology, the American Society of Clinical Oncology, the San Antonio Breast Cancer Symposium conference, and the Chinese Society of Clinical Oncology. Our focus was on studies published between November 2007 and November 2022, specifically those concerning the treatment of HR + /HER2 − advanced postmenopausal women with breast cancer. Considering that in 2007, the US Food and Drug Administration (FDA) updated the guidance for clinical trials in oncology, leading to more consistent choices of endpoints [17]. The following keywords were mainly used: "hormone receptor-positive", "human epidermal factor receptor 2 negative", "advanced/metastatic breast cancer", and "randomized controlled trials". Details of the search strategies are given in Additional file 1: Table S2.

### Inclusion and exclusion criteria

Studies that met the following inclusion criteria and provided a clear description of patient characteristics were included in the NMA:

1. Population: Women with HR + /HER2 − postmenopausal advanced breast cancer.
2. Interventions and comparisons: Single-agent chemotherapy, endocrine therapy monotherapy, targeted therapy, and combinations of endocrine therapy with targeted therapy were considered.
3. Outcome: The HRs of overall survival (OS), progression-free survival (PFS), and the objective response rate were examined. Adverse events (AEs) incidences were categorized into multiple groups: AEs of any grade, grade 3–5 AEs, AEs leading to discontinuation, and AEs leading to death. Attention was also given to the incidence rates of the three most common specific AEs, which included both hematologic and non-hematologic types, across any grade and specifically within grades 3–5. The presence of at least one Kaplan–Meier curve for either OS or PFS was a requirement. If specific data related to postmenopausal women were provided in any RCTs, those trials were included in this study.
4. Study design: phase II or phase III RCTs.

Some articles were excluded based on the following criteria:

1. Papers published before 2007.
2. Non-RCTs or single-arm RCTs.
3. Trials with unclear clinical outcomes.
4. RCTs that included only premenopausal patients and HER2-positive or triple-negative breast cancer patients alone.

All retrieved articles were imported into Note Express (version 3.2.0.7535). The literature was reviewed by two researchers (HS and MZ). Disagreements among the reviewers were settled through discussion. A third reviewer (GY) was consulted if necessary. Titles and abstracts were first screened. The full text of the literature selected for inclusion was then evaluated. Finally, the included literature was reviewed for the inclusion of the most recent data from the relevant studies.

### Data extraction

Baseline characteristics and clinical outcomes were extracted independently by two investigators for participants in each treatment group in the following study designs: RCTs’ names, sample size, median age, and follow-up time. Clinical outcomes extracted included HRs of OS and PFS, any grade AEs, grade 3–5 AEs, AEs leading to discontinuation of treatment, and AEs leading to death. Additionally, we considered the three most common AEs, encompassing both hematologic and non-hematologic types, across all grades and specifically within grades 3–5. Survival data from the independent review committee were prioritized. For trials where independent review committee data were not available, investigator-assessed outcomes were extracted.

### Quality assessment

RCTs were assessed using the Cochrane risk of bias tool measure [18]. We used Egger regression tests with funnel plots to assess publication bias and which were considered significantly asymmetric and publication biased if *p* < 0.1.

### Statistical analysis

Interventions identified in the RCTs were extracted and categorized according to their mechanisms of action, utilizing resources from PubChem [19]. Furthermore, the FDA approval status of the drugs was ascertained by referencing the National Cancer Institute [20]. The Engauge Digitizer (version 4.1) was utilized to extract survival data from PFS and OS Kaplan–Meier curves [21]. Guyot’s method was used to reconstruct individual patient data and then fit the survival data [22]. This is one of the most accurate data-reconstruction methods for cases where individual patient data are not available [23]. Through visual inspection, the reconstructed curves were consistent with the original curves.

The assumption of PH for each trial was first evaluated for time-to-event data, acknowledging that HRs between different treatments often vary over time. Log-cumulative hazard plots and Schoenfeld residual tests were used for this analysis [24, 25]. Clear violations of the PH assumption were detected in several first-line and second/further-lines trials (Additional file 1: Table S3). Therefore, relying solely on the HR derived from the Cox-PH model as the effect measure for NMA is not sufficient. Instead, we fitted a series of first-order fractional polynomial (FP) models with power parameters -2, -1. -0.5, 0.5, 1, 2, and 3. Akaike information criterion was used to assess goodness-of-fit [26, 27]. Frequency models were utilized for time-to-event data outcomes.

When the PH assumption did not hold, FP models were employed. To compare the effects of all treatments, life years for each treatment were calculated within 10 years. This horizon was selected as it is a more representative survival duration for the treatment of advanced breast cancer. When the PH assumption was held, the Cox-PH model was applied using the “netmeta” package in R, version 4.2.2. Additionally, given that some studies did not include Kaplan–Meier curves, we conducted a Cox-PH analysis to establish a comprehensive network and present conservative findings [28]. In the subgroup analysis, factors such as patient age, presence of visceral metastasis, ethnicity, and the occurrence of pivotal gene mutations were considered.

Bayesian NMA was used for safety, which could be realized by the R package "BUGSnet" [29]. The analysis was conducted through four parallel Markov chains comprising 50,000 samples after a 10,000-sample burn-in. We used the "gemtc" package in R, version 4.2.2. Log odds ratios with 95% credible intervals were used as effect sizes. Heterogeneity between studies was assessed using Cochran’s *Q* test and *I*^2^ statistic within a visual forest plot, *I*^2^ statistic > 50%, or the *P* value < 0.1 for the *Q* test was considered as indicating significant heterogeneity, the inconsistency of this model was evaluated using the edge-splitting method, which took into account all direct and indirect evidence [30, 31]. The fixed effect model was only considered when the conditions *I*^2^ < 50% or *P* value of the *Q* test < 0.1 were met, and all points in the leverage plot were within the purple area. In all other circumstances, a random effects model was used [32]. The convergence of Markov chains was checked using trace plots and Gelman-Rubin diagnostic statistics [26].

## Results

### Study selection and characteristics of included studies

**Fig. 1:**
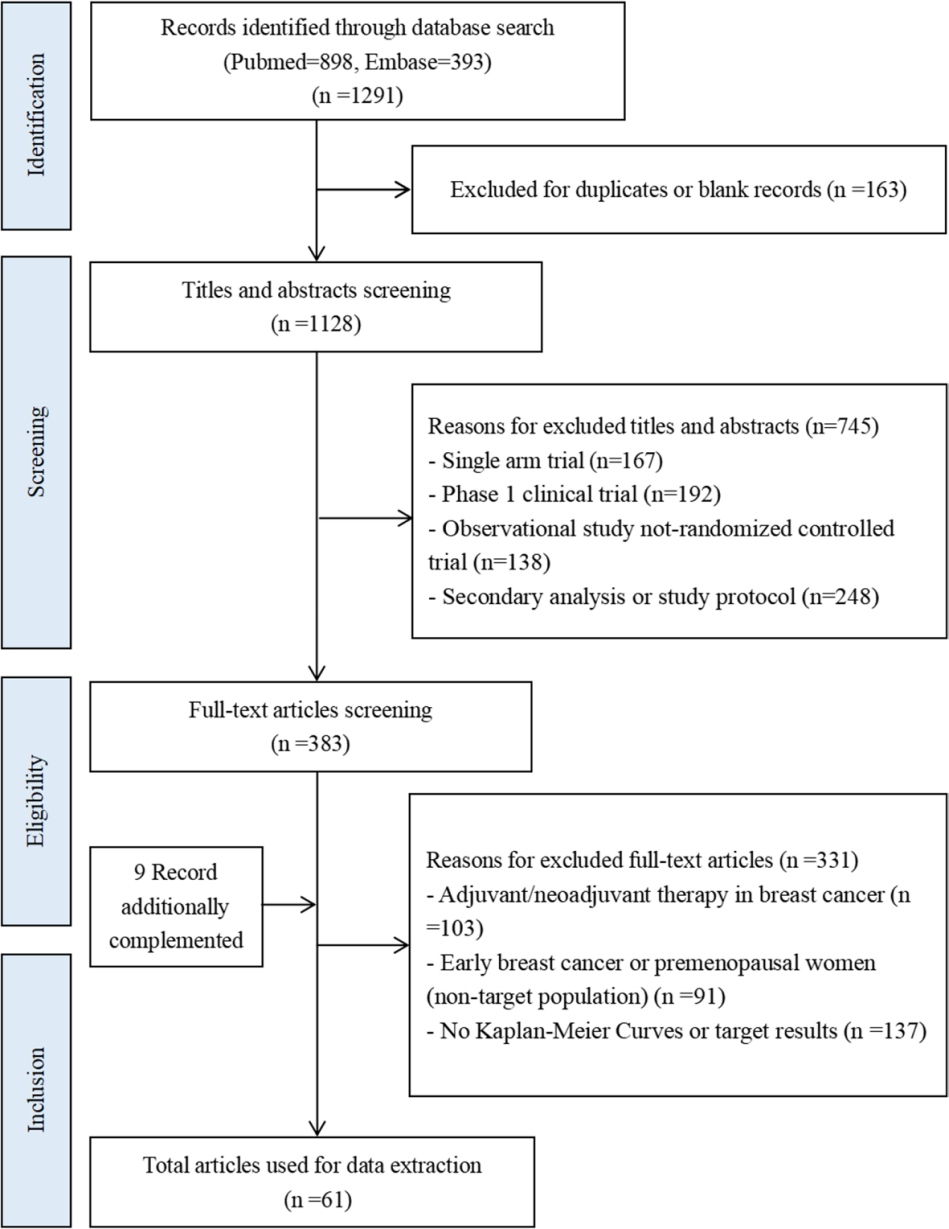
Flow diagram of literature search and study identification

A total of 1291 records were retrieved from the database. After excluding 163 duplicates, 41 RCTs were retained following the primary screening of titles, abstracts, and full-text re-screening based on the PICOS principles. These consisted of 52 full-text articles and 9 abstracts [33–93]. The flow chart of the literature search process is shown in Fig. 1. All included studies exhibited high overall quality. Seventeen RCTs with 7062 patients were included in the first-line analysis, and 27 RCTs with 10,211 patients were included in the second/further-lines analysis (Additional file 1: Table S4-Table S5). The FDA approval status of the drugs is shown in Additional file 1: Table S6.

### Risk of bias

The risk of bias assessment was presented in Additional file 2: Figure S1. Overall, the risk of bias was generally low across all RCTs. However, some included RCTs were open-label, elevating the risk of bias in participant and personnel blinding as well as allocation concealment. The results of the risk of bias assessment were shown in Additional file 2: Figure S2, and Egger regression tests were used to determine publication bias, with *p*-values of 0.69 for first-line PFS and 0.21 for first-line OS, respectively, and 0.14 for second/further-lines PFS and 0.36 for second/further-lines OS. Consequently, there was no publication bias in our network.

### Efficacy outcomes

#### Progression-free survival for first-line treatments

**Fig. 2:**
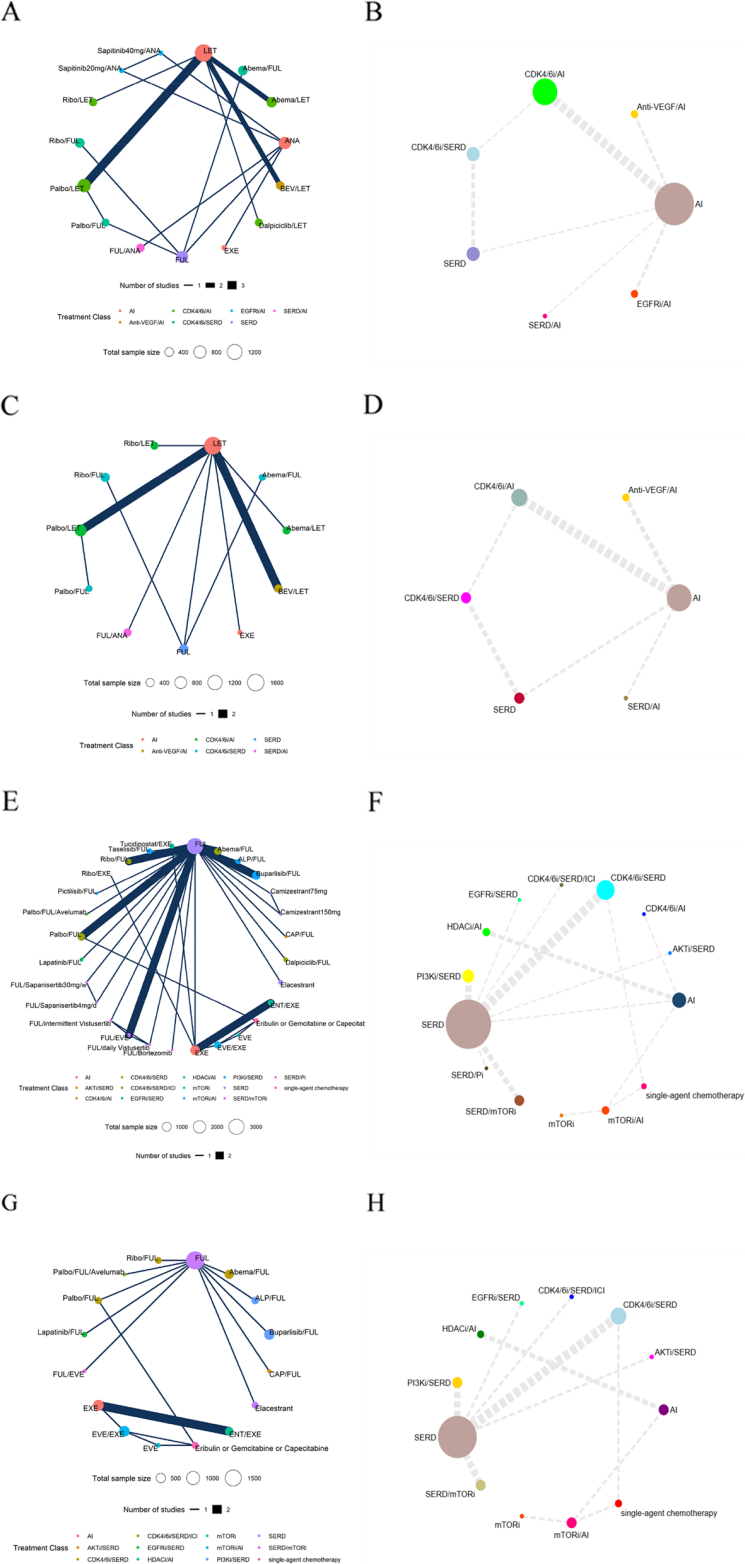
The network plots. (**A** First-line PFS network plot of therapies. **B** First-line PFS network plot of mechanisms. **C** First-line OS network plot of therapies. **D** First-line OS network plot of mechanisms. **E** Second/further-lines PFS network plot of therapies. **F** Second/further-lines PFS network plot of mechanisms. **G** Second/further-lines OS network plot of therapies. **H** Second/further-lines OS network plot of mechanisms). Abbreviations: Abema, Abemaciclib; ALP, Alpelisib; ANA, Anastrozole; BEV, Bevacizumab; CAP, Capivasertib; ENT, Entinostat; EXE, Exemestane; EVE, Everolimus; FUL, Fulvestrant; Palbo, Palbociclib; Ribo, Ribociclib; AI, Aromatase inhibitor; AKTi, AKT inhibitor; Anti-VEGF, Anti-vascular endothelial growth factor; CDK4/6i, Cyclin-dependent kinase 4 and 6 inhibitors; EGFRi: Epidermal growth factor receptor inhibitor; HDACi, Histone deacetylase inhibitor; ICI, Immune checkpoint inhibitors; mTORi, Mammalian target of rapamycin inhibitor; Pi, Protease inhibitor; PI3Ki, Phosphatidylinositol 3-kinase inhibitor; SERD, Selective estrogen receptor degrader

**Fig. 3:**
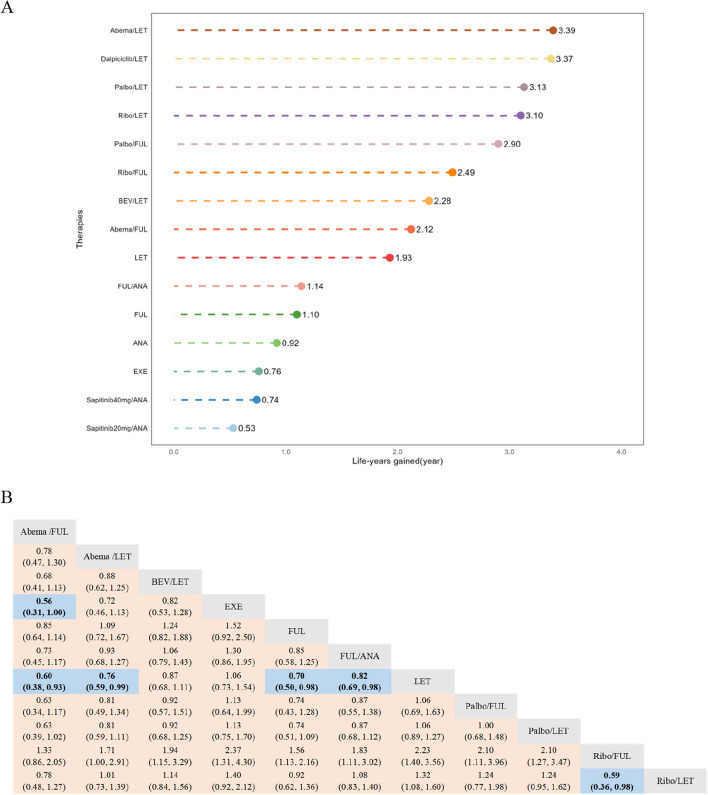
Summary results of efficacy outcomes for the first-line treatment. (**A** Life-year results within 10 years for first-line therapies’ PFS. **B** Cox-PH model result for first-line therapies’ OS). Abbreviations: Abema, Abemaciclib; ANA, Anastrozole; BEV, Bevacizumab; EXE, Exemestane; FUL, Fulvestrant; LET, Letrozole; Palbo, Palbociclib; Ribo, Ribociclib. Note: The direction of the reported relative effects in each cell is defined as treatment on the right vs. treatment on the left. Values < 1 favor the intervention on the right. Values in parenthesis are 95% credible intervals (95% CIs). Bold cells correspond to statistically significant relative effects for the respective treatment categories

The NMA encompassed 15 therapies and 7 mechanisms, respectively (Fig. 2A,B). The PH assumption was invalidated in this network, resulting in the selection of the FP model, which fit the data at power parameters =  − 1 (Additional file 1: Table S7). In terms of 10-year PFS of the therapies (Fig. 3A and Additional file 2: Figure S3A), Abemaciclib/Letrozole demonstrated the best PFS benefit, providing a life-year gain over 10 years of 3.39 years. Dalpiciclib/Letrozole and Palbociclib/Letrozole were found to be comparable to Abemaciclib/Letrozole, with life-years gained over 10 years of 3.37 and 3.13 years, respectively. Bayesian NMA provided consistent treatment rankings for Cox-PH model (Additional file 1: Table S8). Concerning the 10-year PFS of the mechanisms (Fig. 5A and Fig. 5C), CDK4/6i in combination with ET performed the best, with CDK4/6i plus selective estrogen receptor degrader (SERD) (3.48 life years) slightly outperforming CDK4/6i plus aromatase inhibitor (AI) (3.30 life years). A similar trend was observed in the results from the Cox-PH model (Additional file 2: Figure S4).

#### Overall survival for first-line treatments

The NMA respectively incorporated 11 therapies and 6 mechanisms (Fig. 2C,D). The PH assumption was validated in this network, leading to the choice of the Cox-PH model. The results of the Cox-PH model (Fig. 3B) showed that compared with the Letrozole, several treatments, including Abemaciclib/Fulvestrant (HR, 0.60 [95% CI, 0.38 ~ 0.93]), Abemaciclib/Letrozole (0.76 [0.59 ~ 0.99]), Fulvestrant (0.70 [0.50 ~ 0.98]), Ribociclib/Fulvestrant (0.45 [0.28 ~ 0.71]), Ribociclib/Letrozole (0.76 [0.63 ~ 0.92]), and Fulvestrant/Anastrozole (0.82 [0.69 ~ 0.98]) all significantly improved OS in first-line patients to varying extents. Additionally, for first-line mechanisms, whether considering the Cox-PH model (Additional file 2: Figure S4) or the FP model (Fig. 5B,C), the results consistently indicated superior performance of CDK4/6i combined with SERD or AI.

#### Progression-free survival for second/further-line treatments

**Fig. 4:**
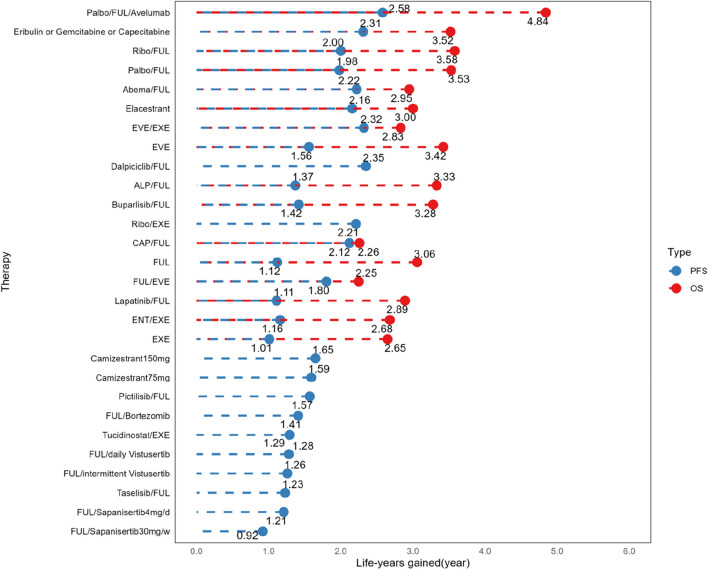
Life-year results within 10 years for second/further-line therapies’ PFS and OS. Abbreviations: Abema, Abemaciclib; ALP, Alpelisib; CAP, Capivasertib; ENT, Entinostat; EVE, Everolimus; EXE, Exemestane; FUL, Fulvestrant; Palbo, Palbociclib; Ribo, Ribociclib

**Fig. 5:**
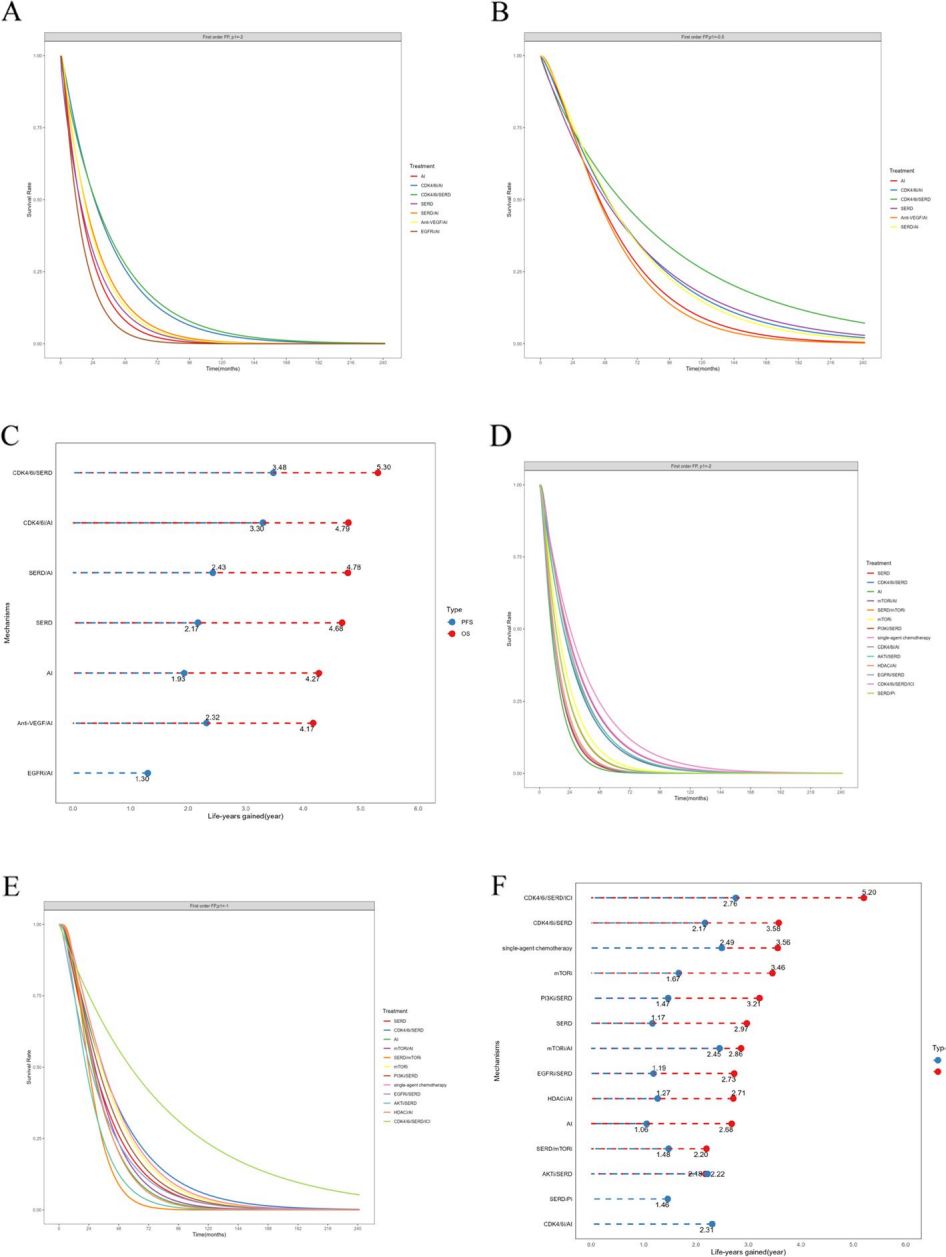
Life-year results within 10 years for first-line and second/further-line mechanisms’ PFS and OS (**A** Extrapolation results of first-line mechanisms’ PFS survival curves based on the Fractional polynomial model. **B** Extrapolation results of first-line mechanisms’ OS survival curves based on the Fractional polynomial model. **C** Fractional polynomial model result for first-line mechanisms’ PFS and OS. **D** Extrapolation results of second/further-lines mechanisms’ PFS survival curves based on the Fractional polynomial model. **E** Extrapolation results of second/further-lines mechanisms’ OS survival curves based on the Fractional polynomial model. **F** Fractional polynomial model result for second/further-line mechanisms’ PFS and OS). Abbreviations: AI, Aromatase inhibitor; AKTi, AKT inhibitor; Anti-VEGF, Anti-vascular endothelial growth factor; CDK4/6i, Cyclin-dependent kinase 4 and 6 inhibitors; EGFRi: Epidermal growth factor receptor inhibitor; HDACi, Histone deacetylase inhibitor; ICI, Immune checkpoint inhibitors; mTORi, Mammalian target of rapamycin inhibitor; Pi, Protease inhibitor; PI3Ki, Phosphatidylinositol 3-kinase inhibitor; SERD, Selective estrogen receptor degrader

In the NMA of second/further-lines PFS, a total of 28 therapies and 14 mechanisms were incorporated (Fig. 2E,F). The PH assumption was invalidated in this network, leading to the selection of the FP model, which fit the data at power parameters =  − 2 (Additional file 1: Table S7). Regarding to 10-year PFS for various therapies (Fig. 4 and Additional file 2: Figure S3B), the combination of Palbociclib, Fulvestrant, and Avelumab emerged as the most effective, contributing to a life-year gain of 2.58 years over a decade. Dalpiciclib/Fulvestrant and Everolimus/Exemestane followed closely, yielding life-year gains of 2.35 and 2.32 years respectively over the same period. Contrarily, results from the Cox-PH model (Additional file 1: Table S9) suggested that single-agent chemotherapy (Eribulin, Gemcitabine, or Capecitabine) outperformed others, with Everolimus/Exemestane ranking second. When examining 10-year PFS for different mechanisms (Fig. 5D and F), the combination of CDK4/6i, SERD, and ICI (2.76 life years) demonstrated the greatest benefit, followed by single-agent chemotherapy (2.49 life years). The Cox-PH model exhibited a similar trend (Additional file 2: Figure S5).

#### Overall survival for second/further-line treatments

In this portion, the NMA incorporated 16 therapies and 12 mechanisms (Fig. 2G,H). The PH assumption was not sustained in this network, prompting the use of the FP model with power parameters set at − 1 (Additional file 1: Table S7). In terms of 10-year OS for therapies (Fig. 4 and Additional file 2: Figure S3C), the combination of Palbociclib, Fulvestrant, and Avelumab exhibited the best OS benefit, contributing to a life-year gain of 4.84 years over a decade. This was followed by Ribociclib/Fulvestrant (3.58 life years) and Palbociclib/Fulvestrant (3.53 life years). However, the results derived from the Cox-PH model (Additional file 1: Table S9) suggested superior performance by Abemaciclib/Fulvestrant. For the mechanisms’ 10-year OS (Fig. 5E,F), the combination of CDK4/6i, SERD, and ICI (5.20 life years) showed the best outcome, followed by CDK4/6i/SERD (3.58 life years), and single-agent chemotherapy (3.56 life years). The Cox-PH model displayed similar results (Additional file 2: Figure S5).

### Safety outcomes

**Fig. 6:**
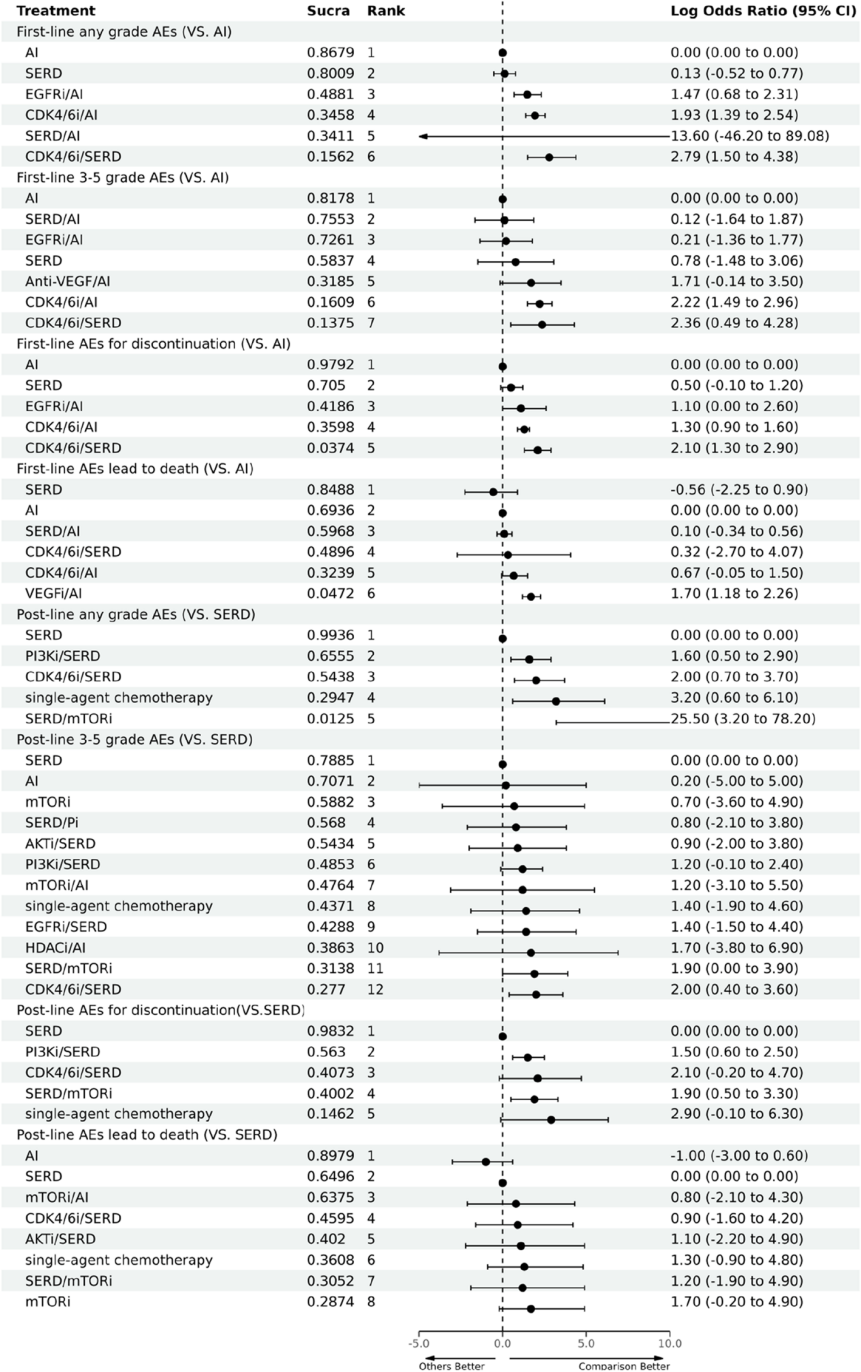
Safety outcomes for first-line and second/further-lines mechanisms (any grade AEs; grade 3–5 AEs; AEs leading to discontinuation; AEs leading to death). Abbreviations: AI, Aromatase inhibitor; AKTi, AKT inhibitor; Anti-VEGF, Anti-vascular endothelial growth factor; CDK4/6i, Cyclin-dependent kinase 4 and 6 inhibitors; EGFRi: Epidermal growth factor receptor inhibitor; HDACi, Histone deacetylase inhibitor; ICI, Immune checkpoint inhibitor; mTORi, Mammalian target of rapamycin inhibitor; Pi, Protease inhibitor; PI3Ki, Phosphatidylinositol 3-kinase inhibitor; SERD, Selective estrogen receptor degrader

Within the scope of first-line therapies, Letrozole consistently exhibits the lowest incidence rate for any grade AEs, grade 3–5 AEs, and AEs resulting in discontinuation. The only exception is the occurrence of AEs leading to death, where Fulvestrant has the lowest incidence rate. The highest incidence rates are observed with Palbociclib/Fulvestrant for any grade AEs, Sapitinib40mg/Anastrozole for grade 3–5 AEs, Ribociclib/Fulvestrant for AEs leading to discontinuation, and Bevacizumab/Letrozole for AEs leading to death. Additional detailed information is available in the Additional file 1: Table S10. Regarding the mechanisms of first-line treatment strategies, AI persistently displays the lowest incidence rates for any AEs, grade 3–5 AEs, AEs leading to treatment cessation, and AEs resulting in death. The highest incidence rates for any grade AEs, grade 3–5 AEs, and AEs resulting in discontinuation are associated with CDK4/6i/SERD. However, for AEs leading to death, the highest incidence rate was observed with the combination of anti-vascular endothelial growth factor and AI. Additional detailed information is available in Fig. 6.

In terms of safety outcomes for second/further-line treatments, Everolimus presents the lowest incidence rate for any grade AEs, Fulvestrant exhibits the best performance in terms of grade 3–5 AEs, Palbociclib/Fulvestrant is optimal in minimizing AEs resulting in treatment discontinuation, and Exemestane shows the lowest rate of AEs leading to death. Conversely, Fulvestrant/Sapanisertib 4mg/day manifests the worst performance in any grade AEs, Buparlisib/Fulvestrant ranks highest in grade 3–5 AEs, Fulvestrant/Sapanisertib 30mg/week leads in AEs causing discontinuation, and Fulvestrant/Everolimus has the highest incidence rate of AEs leading to death. Additional detailed information is available in the Additional file 1: Table S11. In the context of second/further-line mechanisms, SERD demonstrates the lowest incidence rates for any grade AEs, grade 3–5 AEs, and AEs leading to treatment discontinuation. AI is associated with the lowest rate of AEs leading to death. Conversely, a combination of SERD and mammalian target of rapamycin inhibitor (mTORi) demonstrated the highest incidence rate of any grade AEs. CDK4/6i/SERD accounted for the highest occurrence of grade 3–5 AEs. Single-agent chemotherapy was most associated with AEs leading to treatment cessation, while mTORi presented the highest incidence of AEs that resulted in death. More information is available in Fig. 6.

Additionally, it was observed that the incidence of both hematologic and non-hematologic AEs was relatively low with endocrine monotherapy. While most of CDK4/6 inhibitors were associated with an increased incidence of hematologic AEs such as neutropenia and leukopenia, our analysis indicates a more varied profile for non-hematologic AEs, such as Abemaciclib showing a notable increase in events like diarrhea (Additional file 1: Table S12).

### Subgroup analysis results

The subgroup analysis revealed that in the first-line treatment, Palbociclib/Letrozole yielded the greatest PFS benefit for patients aged over 65; conversely, Abemaciclib/Letrozole provided the most significant PFS benefit for patients under 65. For patients with visceral metastasis, the treatment yielding the best PFS benefit in the first-line setting was Abemaciclib/Letrozole, while in the second-line or further-line settings, the best PFS benefit was seen with Abemaciclib/Fulvestrant. In patients harboring mutations in Phosphatidylinositol-4,5-Bisphosphate 3-Kinase Catalytic Subunit Alpha (PIK3CA), both Capivasertib/Fulvestrant and Alpelisib/Fulvestrant regimens substantially improved the PFS. Similarly, for those with Estrogen Receptor 1 (ESR1) mutations, novel oral SERD such as Camizestrant and Elacestrant demonstrated superior PFS benefits compared to traditional SERD (Additional file 2: Figure S6). Furthermore, the incorporation of CDK4/6i into endocrine therapy appeared to yield a more substantial PFS benefit for Asian patients compared to White patients, both in the first-line and subsequent lines of treatment. Additionally, we compared the HRs for PFS and OS of FDA-approved drugs used in the first-line and second/further-line treatment of HR + /HER2 − advanced breast cancer (Additional file 2: Figure S7-Figure S8). The results indicated that among FDA-approved first-line therapies, Ribociclib/Fulvestrant demonstrates the greatest patient benefit in both PFS and OS. In second/further-line treatment, Abemaciclib/Fulvestrant demonstrates superior performance in terms of OS, while single-agent chemotherapy may hold certain advantages in PFS.

Moreover, for the RCTs related to second/further-line treatments, we gathered HRs for PFS and OS from individual second and third-line treatments (Additional file 1: Table S13). Moreover, we performed an analysis using the Cox-PH model for individual second-line PFS and OS, as well as for third-line PFS. More details can be found in Additional file 1: Table S14. Limited data results demonstrate that, within the scope of second-line and subsequent treatments, the integration of various CDK4/6i with Fulvestrant significantly enhances patients’ PFS and OS to diverse degrees.

### Convergence, heterogeneity, and transitivity assessment

The Gelman-Rubin method showed that the three Markov chains were stable and the inferential iterations were reproducible in all models. Overall, the results of the *Q* test, the *I*^2^ statistic, and the forest plots all indicated low heterogeneity across the specific arm. Further details are provided in Additional file 1: Table S15-Table S16. The transitivity assessment for patient baseline age, Eastern Cooperative Oncology Group score, and the proportion of the white race also demonstrated significant consistency and transitivity across the included studies (Additional file 2: Figure S9).

## Discussion

In this research, we identified 41 qualifying trials that constructed scant networks. Most of the treatments within these networks were not subjected to head-to-head trials, underscoring the value of our investigation. We compared the efficacy and safety of therapies and mechanisms in first-line and second/further-line treatment of HR + /HER2 − advanced breast cancer in postmenopausal women.

Our primary findings suggest that combining CDK4/6i with ET (AI or SERD) has solidified its position in first-line treatment due to superior efficacy. Although the incidence of AEs is higher when combining CDK4/6i with endocrine therapy than with endocrine monotherapy, common CDK4/6i-related AEs like neutropenia and leukopenia are well-managed in clinical practice [94], not undermining their first-line treatment status. Subgroup analysis of first-line treatments revealed that patients under 65 years old derived more benefit from CDK4/6i combined with endocrine therapy than those over 65. Patients with visceral metastasis seemed to benefit less than the overall target population. Across different studies, the improvement trend was consistent in terms of race, with the Asian population benefiting more than the white population.

For second/further-line treatment, based on FP results, the addition of ICI to CDK4/6i combined with endocrine therapy performs well though the Cox-PH results do not align, potentially due to the inherent uncertainty in FP model extrapolation leading to unstable results. Regarding safety, endocrine monotherapy performed well. Despite PFS benefits provided by several drugs such as Sapanisertib and Buparlisib, their severe toxicity has precluded further development of these treatment plans. Subgroup analysis reveals varying degrees of PFS improvement in PIK3CA mutation patients treated with Capivasertib and Alpelisib, respectively, in combination with Fulvestrant. For ESR1 mutation patients, significant PFS improvement is observed with Camizestrant and Elacestrant monotherapy, which have been recommended in multiple guidelines and could become the new cornerstone of endocrine therapy for HR + /HER2 − breast cancer patients. The benefits for patients with visceral metastasis are also less than the overall target population.

Clinical practice and the National Comprehensive Cancer Network (NCCN) guidelines [95] both highlight the efficacy of first-line therapies incorporating CDK4/6i. Among these, Abemaciclib/Letrozole offers the most significant enhancement in PFS for first-line patients, while Ribociclib/Fulvestrant optimizes OS. In subsequent line treatment based on CDK4/6i, Abemaciclib/Fulvestrant and Ribociclib/Fulvestrant lead in improving PFS and OS, respectively. When considering safety, Abemaciclib and Palbociclib, when added to first-line endocrine therapy, outshine other CDK4/6 inhibitors. In the subsequent line treatment, Palbociclib takes precedence. Moreover, among the recommended subsequent line treatments incorporating Everolimus with endocrine therapy, the combination of Everolimus and Exemestane exhibits marked superiority in both efficacy and safety compared to other endocrine therapy regimens.

The novel oral SERD Elacestrant has demonstrated promising results in treating breast cancer patients with resistance to endocrine therapy due to ESR1 gene mutations. Its outstanding trial outcomes have culminated in approvals from both the FDA and the European Medicines Agency [96, 97], as well as a recommendation in the most recent NCCN guidelines. Interestingly, in subgroup analysis, Camizestrant seems to surpass Elacestrant in terms of PFS. However, the evidence base for Camizestrant is limited, with only one phase II study included in the evaluation, indicating a need for further research. As for another important breast cancer mutant genotype, the PIK3CA mutation, Alpelisib/Fulvestrant is the recommended treatment according to NCCN guidelines. Alpelisib carries the distinction of being the first FDA-approved Phosphatidylinositol 3-kinase inhibitor. Significantly, Capivasertib/Fulvestrant has also shown encouraging PFS improvements and, intriguingly, Capivasertib has recently become the first FDA-approved AKT pathway inhibitor [98]. This highlights the potential of focusing on the targeted inhibition of the PI3K/AKT/mammalian target of rapamycin signaling pathway as a promising avenue for advancing future breast cancer therapies.

Numerous systematic reviews and NMA of HR + /HER2 − advanced breast cancer have been published in recent years. Laura A. Huppert [4] provided a comprehensive analysis of systemic treatments for both early and metastatic HR + /HER2 − breast cancer, summarizing critical clinical data and exploring its influence on clinical practice. In May 2023, ESMO introduced an online guideline for metastatic breast cancer [99], incorporating all eligible HR + /HER2 − advanced patients into the first-line treatment scope with CDK4/6i. Upon CDK4/6i progression, alternative treatments, including Everolimus/Exemestane, CDK4/6i combinations with endocrine therapy, single-agent Fulvestrant, alpelisib for patients with PIK3CA mutations, Elacestrant for those with ESR1 mutations, are considered. Concurrently, a multitude of meta-analyses assessing the safety and efficacy of HR + /HER2 − advanced breast cancer treatment regimens have been published. All showing that the addition of CDK4/6i to endocrine therapy improves patient prognosis [9, 10, 13, 100–103]. In addition, sacituzumab govitecan-hziy has been approved by the FDA for the treatment of HR + /HER2 − advanced breast cancer patients who have previously undergone endocrine-based treatments and at least two other therapeutic regimens [104]. Additionally, emerging targeted therapies such as ICI and Antibody‐drug Conjugates offer potential new choices for future breast cancer treatment strategies [105, 106].

Initially, we considered both PH and Non-PH model with time-varying HRs and used life years gain as a measure of efficacy. This approach allowed for a clearer comparison of the survival benefits offered by different interventions. Furthermore, we extrapolated survival curves for different interventions based on their mechanisms, to predict long-term outcomes. This significant aspect, overlooked in previous network meta-analysis studies, augments the comprehensiveness of our research. Finally, we also classified the mechanisms of the interventions and compared the efficacy and safety of the different mechanisms, providing a reference for clinical use and new drug development.

To our knowledge, this research is the first to compare the outcomes of currently available first-line and second/further-line treatments for patients with advanced HR + /HER2 − postmenopausal women with advanced breast cancer. As a timely network meta-analysis, we used the most updated data and addressed the study’s relevant significance by making recommendations on treatment choices based on our analysis and the clinical point of view. We used FP models to model the long-term treatment effects of treatment options. In RCTs, it is not possible to accurately estimate the benefit of OS due to the limited follow-up time. The approach used in our study can address this data gap. The results of our study will enable clinicians and patients to determine the survival benefits of emerging treatments and select the best treatment. The results of our subgroup analysis can also help physicians tailor precise treatment regimens for patients of different ages and with different tumor metastatic statuses. Our robust methodology and aggregation mean that our results are reliable and relevant to clinical practice.

However, our research also has certain limitations. First, the biases of different baselines in clinical trials cannot be ignored, and different baseline characteristics and clinical staging may lead to a lack of comparability of data. Second, indirect comparisons increase variance, which may lead to non-significant treatment effects or even eliminate differences between studies. In this network meta-analysis, we did not include clinical trials that were outmoded (such as trials with patients of unknown status such as HER2 and HR,). We separately extracted first-line and second/further-lines metrics (such as HRs of PFS and OS) from the trials. Furthermore, when a subset of postmenopausal patients was included in larger RCTs (peri-menopausal or premenopausal patients accounted for less than 20%), data for HR + /HER2 − patients were separately extracted. Sensitivity analysis was conducted excluding these RCTs to ensure result stability. Despite slight variations in the FP model results (Additional file 2: Figure S10), the overall mechanism ranking remained fundamentally consistent, thereby validating the reliability of the study outcomes. Additionally, in the execution of network meta-analysis, frequency models were utilized for time-to-event data outcomes, while Bayesian models were employed for binary outcomes [14, 107]. No significant differences were discerned in the statistical analysis results between these two approaches. However, this inevitably introduces some heterogeneity and bias into the results. Finally, we did not take into account the frontline treatment status of patients undergoing second/further-line therapy, as patients exhibit variability in their resistance or treatment uptake, which could lead to potential survival bias. Therefore, comparisons of treatment regimens after first-line progression may be subject to certain biases due to the inconsistent baseline medication used by frontline patients. RCTs that devise sequential treatment regimens, such as the SONIA study [108], are worth our attention.

## Conclusions

In conclusion, our NMA demonstrated that the combination of CDK4/6i and ET exhibits superior efficacy in first-line treatment, albeit at the expense of increased adverse events. Notably, enhanced benefits were observed in patients under 65 and within the Asian demographic. The combination of CDK4/6i and SERD displayed remarkable efficacy in second/further-line treatment, and the addition of ICI might enhance this efficacy, notwithstanding discrepancies in the Cox-PH model results. Furthermore, while there are PFS benefits associated with drugs such as Sapanisertib and Buparlisib, their development is hindered by toxicity. Noteworthy PFS improvements were observed in PIK3CA and ESR1 mutation patients treated with Capivasertib, Alpelisib, Camizestrant, and Elacestrant. Further research is necessary to determine the most effective treatment strategies in the HR + /HER2 − advanced breast cancer, and sequencing of these therapies is crucial. Additionally, more trials comparing these novel treatments are warranted to reduce uncertainty in these results.

### Supplementary Information

**Additional file 1:** **Table S1.** PRISMA checklist. **Table S2.** Search strategies. **Table S3.** Constant proportional risk test (probability) for Kaplan-Meier curves. **Table S4.** Baseline characteristics of included studies. **Table S5.** Summary of adverse events in RCTs across various dimensions. **Table S6.** List of drugs approved by the Food and Drug Administration. **Table S7.** Akaike Information Criterion value for survival curves from Fractional Polynomial models. **Table S8.** Cox-PH model analysis: Hazard ratios for PFS in first-line therapies. **Table S9.** Hazard ratios for PFS and OS in second/further-lines therapies: Cox-PH model analysis. **Table S10.** Comparison of adverse events for first-line therapies. **Table S11.** Comparison of adverse events for second/further-lines therapies. **Table S12.** Summary of hematologic and non-hematologic adverse events in RCTs. **Table S13.** Summary of Hazard ratios for post-line RCTs’ PFS and OS. **Table S14.** Hazard ratios for second-line OS and PFS, and third-line PFS from Cox-PH model analysis. **Table S15.** Convergence and heterogeneity assessment. **Table S16.** Heterogeneity assessment information.

**Additional file 2:** **Figure S1.** Methodology quality of the included studies. **Figure S2.** Funnel plots to detect the publication bias of included studies. **Figure S3.** Extrapolation results of therapies’ survival curves based on the Fractional Polynomial model. **Figure S4.** Hazard ratios for PFS and OS from Cox-PH model analysis of first-line mechanisms. **Figure S5.** Hazard ratios for PFS and OS from Cox-PH model analysis of second/further-lines mechanisms. **Figure S6.** Subgroup analysis of Cox-PH model results in first-line and second/further-lines therapies. **Figure S7.** Hazard ratios for PFS and OS from Cox-PH model analysis of FDA-approved first-line therapies. **Figure S8.** Hazard Ratios for PFS and OS from Cox-PH model analysis of FDA-approved second/further-lines therapies. **Figure S9.** Transitivity assessment. **Figure S10.** Sensitivity analysis of patients with full menopause.

## Data Availability

The data used in this study are derived from published studies, and the original datasets can be accessed by reviewing the relevant literature. Furthermore, the datasets supporting the outcomes of this research and the R software code can be obtained from the corresponding author via email.

## Abbreviations

AEs: Adverse events
AI: Aromatase inhibitor
CDK4/6i: Cyclin-dependent kinase 4 and 6 inhibitor
Cox-PH: Cox’s proportional hazards
ESR1: Estrogen receptor 1
ET: Endocrine therapy
FDA: Food and Drug Administration
FP: Fractional polynomial
HER2: Human epidermal growth factor receptor 2
HR: Hormone receptor
HRs: Hazard ratios
ICI: Immune checkpoint inhibitor
mTORi: Mammalian target of rapamycin inhibitor
NCCN: National Comprehensive Cancer Network
NMA: Network meta-analysis
OS: Overall survival
PFS: Progression-free survival
PH: Proportional hazards
PIK3CA: Phosphatidylinositol-4,5-Bisphosphate 3-Kinase Catalytic Subunit Alpha
RCTs: Randomized clinical trials
SERD: Selective estrogen receptor degrader

## References

[1] Sung H, Ferlay J, Siegel RL, Laversanne M, Soerjomataram I, Jemal A (2021). Global Cancer Statistics 2020: GLOBOCAN Estimates of Incidence and Mortality Worldwide for 36 Cancers in 185 Countries. CA Cancer J Clin.

[2] Arnold M, Morgan E, Rumgay H, Mafra A, Singh D, Laversanne M (2022). Current and future burden of breast cancer: Global statistics for 2020 and 2040. Breast.

[3] SEER Cancer Stat Facts. https://seer.cancer.gov/statfacts/. Accessed 17 Dec 2023.

[4] Huppert LA, Gumusay O, Idossa D, Rugo HS (2023). Systemic therapy for hormone receptor-positive/human epidermal growth factor receptor 2-negative early stage and metastatic breast cancer. CA Cancer J Clin.

[5] Giaquinto AN, Sung H, Miller KD, Kramer JL, Newman LA, Minihan A (2022). Breast Cancer Statistics, 2022. CA Cancer J Clin.

[6] Rossing M, Pedersen CB, Tvedskov T, Vejborg I, Talman ML, Olsen LR (2021). Clinical implications of intrinsic molecular subtypes of breast cancer for sentinel node status. Sci Rep.

[7] Mouabbi JA, Singareeka RA, Bassett RJ, Hassan A, Tripathy D, Layman RM (2023). Survival outcomes in patients with hormone receptor-positive metastatic breast cancer with low or no ERBB2 expression treated with targeted therapies plus endocrine therapy. JAMA Netw Open.

[8] Mouabbi JA, Osborne CK, Schiff R, Rimawi MF (2021). Management of hormone receptor-positive, human epidermal growth factor 2-negative metastatic breast cancer. Breast Cancer Res Treat.

[9] Rossi V, Berchialla P, Giannarelli D, Nistico C, Ferretti G, Gasparro S (2019). Should all patients with HR-positive HER2-negative metastatic breast cancer receive CDK 4/6 inhibitor as first-line based therapy? A network meta-analysis of data from the PALOMA 2, MONALEESA 2, MONALEESA 7, MONARCH 3, FALCON, SWOG and FACT trials. Cancers (Basel).

[10] Han Y, Wang J, Wang Z, Xu B (2020). Comparative efficacy and safety of CDK4/6 and PI3K/AKT/mTOR inhibitors in women with hormone receptor-positive, HER2-negative metastatic breast cancer: a systematic review and network meta-analysis. Curr Probl Cancer.

[11] Liu Y, Wu J, Ji Z, Chen L, Zou J, Zheng J (2023). Comparative efficacy and safety of different combinations of three CDK4/6 inhibitors with endocrine therapies in HR+/HER-2 - metastatic or advanced breast cancer patients: a network meta-analysis. BMC Cancer.

[12] Ji D, Luo Y, Wang J, Chen S, Lan B, Ma F (2023). CDK4/6 inhibitors, PI3K/mTOR inhibitors, and HDAC inhibitors as second-line treatments for hormone receptor-positive, HER2-negative advanced breast cancer: a network meta-analysis. BMC Cancer.

[13] Giuliano M, Schettini F, Rognoni C, Milani M, Jerusalem G, Bachelot T (2019). Endocrine treatment versus chemotherapy in postmenopausal women with hormone receptor-positive, HER2-negative, metastatic breast cancer: a systematic review and network meta-analysis. Lancet Oncol.

[14] Freeman SC, Cooper NJ, Sutton AJ, Crowther MJ, Carpenter JR, Hawkins N (2022). Challenges of modelling approaches for network meta-analysis of time-to-event outcomes in the presence of non-proportional hazards to aid decision making: Application to a melanoma network. Stat Methods Med Res.

[15] Austin PC, Fang J, Lee DS (2022). Using fractional polynomials and restricted cubic splines to model non-proportional hazards or time-varying covariate effects in the Cox regression model. Stat Med.

[16] Hutton B, Salanti G, Caldwell DM, Chaimani A, Schmid CH, Cameron C (2015). The PRISMA extension statement for reporting of systematic reviews incorporating network meta-analyses of health care interventions: checklist and explanations. Ann Intern Med.

[17] Guidance for Industry on Clinical Trial Endpoints for the Approval of Cancer Drugs and Biologics; Availability. https://www.federalregister.gov/documents/2007/05/16/E7-9345/guidance-for-industry-on-clinical-trial-endpoints-for-the-approval-of-cancer-drugs-and-biologics. Accessed 16 Dec 2023.

[18] Higgins JP, Altman DG, Gotzsche PC, Juni P, Moher D, Oxman AD (2011). The Cochrane Collaboration’s tool for assessing risk of bias in randomised trials. BMJ.

[19] PubChem. https://pubchem.ncbi.nlm.nih.gov/. Accessed 16 Dec 2023.

[20] Comprehensive Cancer Information. https://www.cancer.gov/. Accessed 16 Dec 2023.

[21] Tierney JF, Stewart LA, Ghersi D, Burdett S, Sydes MR (2007). Practical methods for incorporating summary time-to-event data into meta-analysis. Trials.

[22] Guyot P, Ades AE, Ouwens MJ, Welton NJ (2012). Enhanced secondary analysis of survival data: reconstructing the data from published Kaplan-Meier survival curves. BMC Med Res Methodol.

[23] CHTE2020 sources and synthesis of evidence. https://www.sheffield.ac.uk/nice-dsu/methods-development/chte2020-sources-and-synthesis-evidence. Accessed 16 Dec 2023.

[24] Patricia M. Grambsch TMT. Proportional hazards tests and diagnostics based on weighted residuals. Biometrika. 1994;81:515–26.

[25] Ng’Andu NH. An empirical comparison of statistical tests for assessing the proportional hazards assumption of Cox’s model. Stat Med. 1997;16:611–26. 10.1002/(sici)1097-0258(19970330)16:6<611::aid-sim437>3.0.co;2-t 9131751

[26] Brooks SP, Gelman A (1998). General methods for monitoring convergence of iterative simulations. J Comput Graph Stat.

[27] Cooper M, Smith S, Williams T, Aguiar-Ibanez R (2022). How accurate are the longer-term projections of overall survival for cancer immunotherapy for standard versus more flexible parametric extrapolation methods?. J Med Econ.

[28] Wiksten A, Hawkins N, Piepho HP, Gsteiger S (2020). Nonproportional hazards in network meta-analysis: efficient strategies for model building and analysis. Value Health.

[29] Beliveau A, Boyne DJ, Slater J, Brenner D, Arora P (2019). BUGSnet: an R package to facilitate the conduct and reporting of Bayesian network Meta-analyses. BMC Med Res Methodol.

[30] van Valkenhoef G, Dias S, Ades AE, Welton NJ (2016). Automated generation of node-splitting models for assessment of inconsistency in network meta-analysis. Res Synth Methods.

[31] Dias S, Welton NJ, Caldwell DM, Ades AE (2010). Checking consistency in mixed treatment comparison meta-analysis. Stat Med.

[32] Zhao M, Shao T, Ren Y, Zhou C, Tang W (2022). Identifying optimal PD-1/PD-L1 inhibitors in first-line treatment of patients with advanced squamous non-small cell lung cancer in China: Updated systematic review and network meta-analysis. Front Pharmacol.

[33] Turner NC, Ro J, Andre F, Loi S, Verma S, Iwata H (2015). Palbociclib in hormone-receptor-positive advanced breast cancer. N Engl J Med.

[34] Turner NC, Slamon DJ, Ro J, Bondarenko I, Im SA, Masuda N (2018). Overall survival with palbociclib and fulvestrant in advanced breast cancer. N Engl J Med.

[35] Loibl S, Turner NC, Ro J, Cristofanilli M, Iwata H, Im S-A (2017). Palbociclib combined with fulvestrant in premenopausal women with advanced breast cancer and prior progression on endocrine therapy: PALOMA-3 Results. Oncologist.

[36] Cristofanilli M, Rugo HS, Im S-A, Slamon DJ, Harbeck N, Bondarenko I (2022). Overall survival with palbociclib and fulvestrant in women with HR+/HER2- ABC: updated exploratory analyses of PALOMA-3, a double-blind, phase III randomized study. Clin Cancer Res.

[37] Finn RS, Martin M, Rugo HS, Jones S, Im SA, Gelmon K (2016). Palbociclib and letrozole in advanced breast cancer. N Engl J Med.

[38] Rugo HS, Finn RS, Diéras V, Ettl J, Lipatov O, Joy AA (2019). Palbociclib plus letrozole as first-line therapy in estrogen receptor-positive/human epidermal growth factor receptor 2-negative advanced breast cancer with extended follow-up. Breast Cancer Res Treat.

[39] Finn RS, Rugo HS, Dieras VC, Harbeck N, Im S-A, Gelmon KA, et al. Overall survival (OS) with first-line palbociclib plus letrozole (PAL+LET) versus placebo plus letrozole (PBO+LET) in women with estrogen receptor–positive/human epidermal growth factor receptor 2–negative advanced breast cancer (ER+/HER2− ABC): analyses from PALOMA-2. J Clin Oncol. 2022;40 17_suppl:LBA1003–LBA1003.

[40] Xu B, Hu X, Li W, Sun T, Shen K, Wang S (2022). Palbociclib plus letrozole versus placebo plus letrozole in Asian postmenopausal women with oestrogen receptor–positive/human epidermal growth factor receptor 2–negative advanced breast cancer: Primary results from PALOMA-4. Eur J Cancer.

[41] Finn RS, Crown JP, Lang I, Boer K, Bondarenko IM, Kulyk SO (2015). The cyclin-dependent kinase 4/6 inhibitor palbociclib in combination with letrozole versus letrozole alone as first-line treatment of oestrogen receptor-positive, HER2-negative, advanced breast cancer (PALOMA-1/TRIO-18): a randomised phase 2 study. Lancet Oncol.

[42] Finn RS, Boer K, Bondarenko I, Patel R, Pinter T, Schmidt M (2020). Overall survival results from the randomized phase 2 study of palbociclib in combination with letrozole versus letrozole alone for first-line treatment of ER+/HER2− advanced breast cancer (PALOMA-1, TRIO-18). Breast Cancer Res Treat.

[43] Hortobagyi GN, Stemmer SM, Burris HA, Yap YS, Sonke GS (2016). Ribociclib as first-line therapy for HR-positive, advanced breast cancer. N Engl J Med.

[44] Hortobagyi GN, Stemmer SM, Burris HA, Yap YS, Sonke GS, Paluch-Shimon S (2018). Updated results from MONALEESA-2, a phase III trial of first-line ribociclib plus letrozole versus placebo plus letrozole in hormone receptor-positive, HER2-negative advanced breast cancer. Ann Oncol.

[45] Hortobagyi GN, Stemmer SM, Burris HA, Yap Y-S, Sonke GS, Hart L (2022). Overall survival with ribociclib plus letrozole in advanced breast cancer. N Engl J Med.

[46] Slamon DJ, Neven P, Chia S, Fasching PA, De Laurentiis M, Im SA (2018). Phase III randomized study of ribociclib and fulvestrant in hormone receptor-positive, human epidermal growth factor receptor 2-negative advanced breast cancer: MONALEESA-3. J Clin Oncol.

[47] Slamon DJ, Neven P, Chia S, Jerusalem G, De Laurentiis M, Im S (2021). Ribociclib plus fulvestrant for postmenopausal women with hormone receptor-positive, human epidermal growth factor receptor 2-negative advanced breast cancer in the phase III randomized MONALEESA-3 trial: updated overall survival. Ann Oncol.

[48] Sledge GW, Toi M, Neven P, Sohn J, Inoue K, Pivot X (2020). The effect of abemaciclib plus fulvestrant on overall survival in hormone receptor–positive, ERBB2-negative breast cancer that progressed on endocrine therapy—MONARCH 2. JAMA Oncol.

[49] Neven P, Johnston SRD, Toi M, Sohn J, Inoue K, Pivot X (2021). MONARCH 2: subgroup analysis of patients receiving abemaciclib plus fulvestrant as first-line and second-line therapy for HR+, HER2−-advanced breast cancer. Clin Cancer Res.

[50] Johnston S, Martin M, Di Leo A, Im S-A, Awada A, Forrester T (2019). MONARCH 3 final PFS: a randomized study of abemaciclib as initial therapy for advanced breast cancer. NPJ Breast Cancer.

[51] Goetz MP, Toi M, Campone M, Sohn J, Paluch-Shimon S, Huober J (2017). MONARCH 3: abemaciclib as initial therapy for advanced breast cancer. J Clin Oncol.

[52] Goetz MP, Toi M, Huober J, Sohn J, Tredan O, Park IH (2022). LBA15 MONARCH 3: interim overall survival (OS) results of abemaciclib plus a nonsteroidal aromatase inhibitor (NSAI) in patients (pts) with HR+, HER2- advanced breast cancer (ABC). Ann Oncol.

[53] Llombart-Cussac A, Pérez-García JM, Bellet M, Dalenc F, Gil-Gil M, Ruíz-Borrego M (2021). Fulvestrant-palbociclib vs letrozole-palbociclib as initial therapy for endocrine-sensitive, hormone receptor-positive, ERBB2-negative advanced breast cancer. JAMA Oncol.

[54] Albanell J, Martínez MT, Ramos M, O’Connor M, de la Cruz-Merino L, Santaballa A (2022). Randomized phase II study of fulvestrant plus palbociclib or placebo in endocrine-sensitive, hormone receptor-positive/HER2–advanced breast cancer: GEICAM/2014–12 (FLIPPER). Eur J Cancer.

[55] Jiang Z, Hu X, Zhang Q, Sun T, Yin Y, Li H (2019). LBA25 - MONARCHplus: a phase III trial of abemaciclib plus nonsteroidal aromatase inhibitor (NSAI) or fulvestrant (F) for women with HR+/HER2- advanced breast cancer (ABC). Ann Oncol.

[56] Zhang QY, Sun T, Yin YM, Li HP, Yan M, Tong ZS (2020). MONARCH plus: abemaciclib plus endocrine therapy in women with HR+/HER2– advanced breast cancer: the multinational randomized phase III study. Ther Adv Med Oncol.

[57] Xu B, Zhang Q, Zhang P, Hu X, Li W, Tong Z (2021). Dalpiciclib or placebo plus fulvestrant in hormone receptor-positive and HER2-negative advanced breast cancer: a randomized, phase 3 trial. Nat Med.

[58] Zhang P, Zhang QY, Hu X, Li W, Tong Z, Sun T (2022). 229P Dalpiciclib plus fulvestrant in HR+/HER2− advanced breast cancer (ABC): updated analysis from the phase III DAWNA-1 trial. Ann Oncol.

[59] Xu B, Zhang QY, Zhang P, Tong Z, Sun T, Li W (2022). LBA16 Dalpiciclib plus letrozole or anastrozole as first-line treatment for HR+/HER2- advanced breast cancer (DAWNA-2): A phase III trial. Ann Oncol.

[60] Yardley DA, Noguchi S, Pritchard KI, Burris HA, Baselga J, Gnant M (2013). Everolimus plus exemestane in postmenopausal patients with HR+ breast cancer: BOLERO-2 final progression-free survival analysis. Adv Ther.

[61] Piccart M, Hortobagyi GN, Campone M, Pritchard KI, Lebrun F, Ito Y (2014). Everolimus plus exemestane for hormone-receptor-positive, human epidermal growth factor receptor-2-negative advanced breast cancer: overall survival results from BOLERO-2. Ann Oncol.

[62] Schmid P, Zaiss M, Harper-Wynne C, Ferreira M, Dubey S, Chan S (2019). Fulvestrant plus vistusertib vs fulvestrant plus everolimus vs fulvestrant alone for women with hormone receptor–positive metastatic breast cancer. JAMA Oncol.

[63] Kornblum N, Zhao F, Manola J, Klein P, Ramaswamy B, Brufsky A (2018). Randomized phase II trial of fulvestrant plus everolimus or placebo in postmenopausal women with hormone receptor-positive, human epidermal growth factor receptor 2-negative metastatic breast cancer resistant to aromatase inhibitor therapy: results of PrE0102. J Clin Oncol.

[64] Jerusalem G, de Boer RH, Hurvitz S, Yardley DA, Kovalenko E, Ejlertsen B (2018). Everolimus plus exemestane vs everolimus or capecitabine monotherapy for estrogen receptor–positive, her2-negative advanced breast cancer. JAMA Oncol.

[65] Mehta RS, Barlow WE, Albain KS, Vandenberg TA, Dakhil SR, Tirumali NR (2012). Combination anastrozole and fulvestrant in metastatic breast cancer. N Engl J Med.

[66] Mehta RS, Barlow WE, Albain KS, Vandenberg TA, Dakhil SR, Tirumali NR (2019). Overall survival with fulvestrant plus anastrozole in metastatic breast cancer. N Engl J Med.

[67] Robertson JFR, Bondarenko IM, Trishkina E, Dvorkin M, Panasci L, Manikhas A (2016). Fulvestrant 500 mg versus anastrozole 1 mg for hormone receptor-positive advanced breast cancer (FALCON): an international, randomised, double-blind, phase 3 trial. The Lancet.

[68] Di Leo A, Johnston S, Lee KS, Ciruelos E, Lønning PE, Janni W (2018). Buparlisib plus fulvestrant in postmenopausal women with hormone-receptor-positive, HER2-negative, advanced breast cancer progressing on or after mTOR inhibition (BELLE-3): a randomised, double-blind, placebo-controlled, phase 3 trial. Lancet Oncol.

[69] Andre F, Ciruelos E, Rubovszky G, Campone M, Loibl S, Rugo HS (2019). alpelisib for PIK3CA-mutated, hormone receptor-positive advanced breast cancer. N Engl J Med.

[70] André F, Ciruelos EM, Juric D, Loibl S, Campone M, Mayer IA (2021). Alpelisib plus fulvestrant for PIK3CA-mutated, hormone receptor-positive, human epidermal growth factor receptor-2–negative advanced breast cancer: final overall survival results from SOLAR-1. Ann Oncol.

[71] Howell SJ, Casbard A, Carucci M, Ingarfield K, Butler R, Morgan S (2022). Fulvestrant plus capivasertib versus placebo after relapse or progression on an aromatase inhibitor in metastatic, oestrogen receptor-positive, HER2-negative breast cancer (FAKTION): overall survival, updated progression-free survival, and expanded biomarker analysis from a randomised, phase 2 trial. Lancet Oncol.

[72] Jiang Z, Li W, Hu X, Zhang Q, Sun T, Cui S (2019). Tucidinostat plus exemestane for postmenopausal patients with advanced, hormone receptor-positive breast cancer (ACE): a randomised, double-blind, placebo-controlled, phase 3 trial. Lancet Oncol.

[73] Campone M, Im S-A, Iwata H, Clemons M, Ito Y, Awada A (2018). Buparlisib plus fulvestrant versus placebo plus fulvestrant for postmenopausal, hormone receptor-positive, human epidermal growth factor receptor 2-negative, advanced breast cancer: Overall survival results from BELLE-2. Eur J Cancer.

[74] Baselga J, Im S-A, Iwata H, Cortés J, De Laurentiis M, Jiang Z (2017). Buparlisib plus fulvestrant versus placebo plus fulvestrant in postmenopausal, hormone receptor-positive, HER2-negative, advanced breast cancer (BELLE-2): a randomised, double-blind, placebo-controlled, phase 3 trial. Lancet Oncol.

[75] Krop IE, Mayer IA, Ganju V, Dickler M, Johnston S, Morales S (2016). Pictilisib for oestrogen receptor-positive, aromatase inhibitor-resistant, advanced or metastatic breast cancer (FERGI): a randomised, double-blind, placebo-controlled, phase 2 trial. Lancet Oncol.

[76] Martín M, Loibl S, von Minckwitz G, Morales S, Martinez N, Guerrero A (2015). Phase III trial evaluating the addition of bevacizumab to endocrine therapy as first-line treatment for advanced breast cancer: the letrozole/fulvestrant and avastin (LEA) study. J Clin Oncol.

[77] García-Sáenz JÁ, Martínez-Jáñez N, Cubedo R, Jerez Y, Lahuerta A, González-Santiago S (2022). Sapanisertib plus fulvestrant in postmenopausal women with estrogen receptor–positive/HER2-negative advanced breast cancer after progression on aromatase inhibitor. Clin Cancer Res.

[78] Dent S, Cortés J, Im YH, Diéras V, Harbeck N, Krop IE (2021). Phase III randomized study of taselisib or placebo with fulvestrant in estrogen receptor-positive, PIK3CA-mutant, HER2-negative, advanced breast cancer: the SANDPIPER trial. Ann Oncol.

[79] Bidard FC, Kaklamani VG, Neven P, Streich G, Montero AJ, Forget F (2022). Elacestrant (oral selective estrogen receptor degrader) versus standard endocrine therapy for estrogen receptor-positive, human epidermal growth factor receptor 2-negative advanced breast cancer: results from the randomized phase III EMERALD trial. J Clin Oncol.

[80] Connolly RM, Zhao F, Miller KD, Lee MJ, Piekarz RL, Smith KL, et al. E2112: Randomized phase III trial of endocrine therapy plus entinostat or placebo in hormone receptor-positive advanced breast cancer. A trial of the ECOG-ACRIN Cancer Research Group. J Clin Oncol. 2021;39:3171–81. 10.1200/JCO.21.00944 PMC8478386 34357781

[81] Yardley DA, Ismail-Khan RR, Melichar B, Lichinitser M, Munster PN, Klein PM (2013). Randomized Phase II, double-blind, placebo-controlled study of exemestane with or without entinostat in postmenopausal women with locally recurrent or metastatic estrogen receptor-positive breast cancer progressing on treatment with a nonsteroidal aromatase inhibitor. J Clin Oncol.

[82] Johnston S, Basik M, Hegg R, Lausoontornsiri W, Grzeda L, Clemons M (2016). Inhibition of EGFR, HER2, and HER3 signaling with AZD8931 in combination with anastrozole as an anticancer approach: phase II randomized study in women with endocrine-therapy-naïve advanced breast cancer. Breast Cancer Res Treat.

[83] Iwata H, Masuda N, Ohno S, Rai Y, Sato Y, Ohsumi S (2013). A randomized, double-blind, controlled study of exemestane versus anastrozole for the first-line treatment of postmenopausal Japanese women with hormone-receptor-positive advanced breast cancer. Breast Cancer Res Treat.

[84] Dickler MN, Barry WT, Cirrincione CT, Ellis MJ, Moynahan ME, Innocenti F (2016). Phase III trial evaluating letrozole as first-line endocrine therapy with or without bevacizumab for the treatment of postmenopausal women with hormone receptor–positive advanced-stage breast cancer: CALGB 40503 (Alliance). J Clin Oncol.

[85] Chia S, Gradishar W, Mauriac L, Bines J, Amant F, Federico M (2008). Double-blind, randomized placebo controlled trial of fulvestrant compared with exemestane after prior nonsteroidal aromatase inhibitor therapy in postmenopausal women with hormone receptor–positive, advanced breast cancer: results from EFECT. J Clin Oncol.

[86] Burstein HJ, Cirrincione CT, Barry WT, Chew HK, Tolaney SM, Lake DE (2014). Endocrine therapy with or without inhibition of epidermal growth factor receptor and human epidermal growth factor receptor 2: a randomized, double-blind, placebo-controlled phase III trial of fulvestrant with or without lapatinib for postmenopausal women with hormone receptor–positive advanced breast cancer—CALGB 40302 (Alliance). J Clin Oncol.

[87] Adelson K, Ramaswamy B, Sparano JA, Christos PJ, Wright JJ, Raptis G (2016). Randomized phase II trial of fulvestrant alone or in combination with bortezomib in hormone receptor-positive metastatic breast cancer resistant to aromatase inhibitors: a New York Cancer Consortium trial. NPJ Breast Cancer.

[88] Kalinsky K, Accordino MK, Chiuzan C, Mundi PS, Trivedi MS, Novik Y, et al. A randomized, phase II trial of fulvestrant or exemestane with or without ribociclib after progression on anti-estrogen therapy plus cyclin-dependent kinase 4/6 inhibition (CDK 4/6i) in patients (pts) with unresectable or hormone receptor–positive (HR+), HER2-negative metastatic breast cancer (MBC): MAINTAIN trial. J Clin Oncol. 2022;40 17_suppl:LBA1004–LBA1004. 10.1200/JCO.22.02392 37207300

[89] Mayer EL, Wander SA, Regan MM, DeMichele AM, Forero A, Rimawi MF, et al. Abstract OT3–05–11: Palbociclib after CDK inhibitor and endocrine therapy (PACE): A randomized phase II study of fulvestrant versus palbociclib plus fulvestrant, with and without avelumab, for CDK inhibitor pre-treated HR+/HER2- metastatic breast cancer. Cancer Res. 2018;78 4_Supplement:OT3–05–11-OT3–05–11.

[90] Mayer EL, Ren Y, Wagle N, Mahtani R, Ma C, DeMichele A, et al. Abstract GS3–06: GS3–06 Palbociclib After CDK4/6i and Endocrine Therapy (PACE): A Randomized Phase II Study of Fulvestrant, Palbociclib, and Avelumab for Endocrine Pre-treated ER+/HER2- Metastatic Breast Cancer. Cancer Res. 2023;83 5_Supplement:GS3–06-GS3–06.

[91] Oliveira M, Pominchuck D, Nowecki Z, Hamilton E, Kulyaba Y, Andabekov T, et al. Abstract GS3–02: GS3–02 Camizestrant, a next generation oral SERD vs fulvestrant in post-menopausal women with advanced ER-positive HER2-negative breast cancer: Results of the randomized, multi-dose Phase 2 SERENA-2 trial. Cancer Res. 2023;83 5_Supplement:GS3–02-GS3–02. 10.1016/S1470-2045(24)00387-5 39481395

[92] Martin M, Zielinski C, Ruiz-Borrego M, Carrasco E, Turner N, Ciruelos EM (2021). Palbociclib in combination with endocrine therapy versus capecitabine in hormonal receptor-positive, human epidermal growth factor 2-negative, aromatase inhibitor-resistant metastatic breast cancer: a phase III randomised controlled trial—PEARL. Ann Oncol.

[93] Martín M, Zielinski C, Ruiz-Borrego M, Carrasco E, Ciruelos EM, Muñoz M (2022). Overall survival with palbociclib plus endocrine therapy versus capecitabine in postmenopausal patients with hormone receptor-positive, HER2-negative metastatic breast cancer in the PEARL study. Eur J Cancer.

[94] Stanciu IM, Parosanu AI, Nitipir C (2023). An overview of the safety profile and clinical impact of CDK4/6 inhibitors in breast cancer-A systematic review of randomized phase II and III clinical trials. Biomolecules.

[95] Gradishar WJ, Moran MS, Abraham J, Abramson V, Aft R, Agnese D, et al. NCCN Guidelines® Insights: Breast Cancer, Version 4.2023. J Natl Compr Cancer Netw JNCCN. 2023;21:594–608. 10.6004/jnccn.2023.0031 37308117

[96] FDA approves elacestrant for ER-positive, HER2-negative, ESR1-mutated advanced or metastatic breast cancer. 2023. https://www.fda.gov/drugs/resources-information-approved-drugs/fda-approves-elacestrant-er-positive-her2-negative-esr1-mutated-advanced-or-metastatic-breast-cancer. Accessed 16 Dec 2023.

[97] EMA Recommends Granting a Marketing Authorisation for Elacestrant. https://www.esmo.org/oncology-news/ema-recommends-granting-a-marketing-authorisation-for-elacestrant. Accessed 16 Dec 2023.

[98] FDA approves capivasertib with fulvestrant for breast cancer. 2023. https://www.fda.gov/drugs/resources-information-approved-drugs/fda-approves-capivasertib-fulvestrant-breast-cancer. Accessed 16 Dec 2023. 10.1002/cncr.35238 38396318

[99] ESMO Living Guidelines | ESMO. https://www.esmo.org/living-guidelines/esmo-metastatic-breast-cancer-living-guideline. Accessed 16 Dec 2023.

[100] Lee CH, Kang YN, Ho CL, Lin C, Chen PH, Wu YY (2020). Endocrine therapies in postmenopausal women with hormone receptor-positive, human epidermal growth factor receptor 2-negative, pretreated, advanced breast cancer: A network meta-analysis. Med Baltim.

[101] Munzone E, Pagan E, Bagnardi V, Montagna E, Cancello G, Dellapasqua S (2021). Systematic review and meta-analysis of post-progression outcomes in ER+/HER2- metastatic breast cancer after CDK4/6 inhibitors within randomized clinical trials. ESMO Open.

[102] Brandao M, Maurer C, Ziegelmann PK, Ponde NF, Ferreira A, Martel S. Endocrine therapy-based treatments in hormone receptor-positive/HER2-negative advanced breast cancer: systematic review and network meta-analysis. ESMO Open. 2020;5:e000842. 10.1136/esmoopen-2020-000842 PMC7451473 32847835

[103] Tian Q, Gao H, Zhou Y, Yang J (2021). Overall survival and progression-free survival with cyclin-dependent kinase 4/6 inhibitors plus endocrine therapy in breast cancer: an updated meta-analysis of randomized controlled trials. Eur Rev Med Pharmacol Sci.

[104] FDA approves sacituzumab govitecan-hziy for HR-positive breast cancer. 2023. https://www.fda.gov/drugs/resources-information-approved-drugs/fda-approves-sacituzumab-govitecan-hziy-hr-positive-breast-cancer. Accessed 16 Dec 2023.

[105] Xiao T, Ali S, Mata D, Lohmann AE, Blanchette PS (2023). Antibody-Drug Conjugates in Breast Cancer: Ascent to Destiny and Beyond-A 2023 Review. Curr Oncol.

[106] Ma J, Chan JJ, Toh CH, Yap YS (2023). Emerging systemic therapy options beyond CDK4/6 inhibitors for hormone receptor-positive HER2-negative advanced breast cancer. NPJ Breast Cancer.

[107] Sadeghirad B, Foroutan F, Zoratti MJ, Busse JW, Brignardello-Petersen R, Guyatt G (2023). Theory and practice of Bayesian and frequentist frameworks for network meta-analysis. BMJ Evid Based Med.

[108] van Ommen-Nijhof A, Konings IR, van Zeijl C, Uyl-de GC, van der Noort V, Jager A (2018). Selecting the optimal position of CDK4/6 inhibitors in hormone receptor-positive advanced breast cancer - the SONIA study: study protocol for a randomized controlled trial. BMC Cancer.

