# Supplementary material for: A network meta-analysis of efficacy and safety for first-line and second/further-line therapies in postmenopausal women with hormone receptor-positive, HER2-negative, advanced breast cancer
Source: BMC Med. 2024 Jan 12;22:13. doi: 10.1186/s12916-023-03238-2 (PMC10785354; doi:10.1186/s12916-023-03238-2)
Supplement: Supplementary file 1 — Additional file 1: Table S1. PRISMA checklist. Table S2. Search strategies. Table S3. Constant proportional risk test (probability) for Kaplan-Meier curves. Table S4. Baseline characteristics of included studies. Table S5. Summary of adverse events in RCTs across various dimensions. Table S6. List of drugs approved by the Food and Drug Administration. Table S7. Akaike Information Criterion value for survival curves from Fractional Polynomial models. Table S8. Cox-PH model analysis: Hazard ratios for PFS in first-line therapies. Table S9. Hazard ratios for PFS and OS in second/further-lines therapies: Cox-PH model analysis. Table S10. Comparison of adverse events for first-line therapies. Table S11. Comparison of adverse events for second/further-lines therapies. Table S12. Summary of hematologic and non-hematologic adverse events in RCTs. Table S13. Summary of Hazard ratios for post-line RCTs’ PFS and OS. Table S14. Hazard ratios for second-line OS and PFS, and third-line PFS from Cox-PH model analysis. Table S15. Convergence and heterogeneity assessment. Table S16. Heterogeneity assessment information. [file 12916_2023_3238_MOESM1_ESM.docx]

**Additional file 1**

[Table S1 PRISMA checklist 2](#_Toc27753)

[Table S2 Search strategies 6](#_Toc7361)

[Table S3 Constant proportional risk test (probability) for Kaplan-Meier curves 9](#_Toc11395)

[Table S4 Baseline characteristics of included studies 11](#_Toc7759)

[Table S5 Summary of adverse events in RCTs across various dimensions 22](#_Toc7014)

[Tbale S6 List of drugs approved by the Food and Drug Administration 26](#_Toc19360)

[Table S7 Akaike Information Criterion value for survival curves from Fractional Polynomial models 28](#_Toc10148)

[Table S8 Cox-PH model analysis: Hazard ratios for PFS in first-line therapies 30](#_Toc25585)

[Table S9 Hazard ratios for PFS and OS in second/further-lines therapies: Cox-PH model analysis 30](#_Toc15027)

[Table S10 Comparison of adverse events for first-line therapies 33](#_Toc23902)

[Table S11 Comparison of adverse events for second/further-lines therapies 36](#_Toc25269)

[Table S12 Summary of hematologic and non-hematologic adverse events in RCTs 39](#_Toc9241)

[Table S13 Summary of Hazard ratios for post-line RCTs’ PFS and OS 48](#_Toc24418)

[Table S14 Hazard ratios for second-line OS and PFS, and third-line PFS from Cox-PH model analysis 49](#_Toc5147)

[Table S15 Convergence and heterogeneity assessment 51](#_Toc21352)

[Table S16 Heterogeneity assessment information 52](#_Toc2438)

# Table S1 PRISMA checklist

| **Section and Topic** | **Item #** | **Checklist item** | **Location where item is reported** |
| --- | --- | --- | --- |
| **TITLE** | | |  |
| Title | 1 | Identify the report as a systematic review. | P1 |
| **ABSTRACT** | | |  |
| Abstract | 2 | See the PRISMA 2020 for Abstracts checklist. | P1-P2 |
| **INTRODUCTION** | | |  |
| Rationale | 3 | Describe the rationale for the review in the context of existing knowledge. | P3-P4 |
| Objectives | 4 | Provide an explicit statement of the objective(s) or question(s) the review addresses. | P3-P4 |
| **METHODS** | | |  |
| Eligibility criteria | 5 | Specify the inclusion and exclusion criteria for the review and how studies were grouped for the syntheses. | P5-P7 |
| Information sources | 6 | Specify all databases, registers, websites, organisations, reference lists and other sources searched or consulted to identify studies. Specify the date when each source was last searched or consulted. | P4-P5 |
| Search strategy | 7 | Present the full search strategies for all databases, registers and websites, including any filters and limits used. | P5 |
| Selection process | 8 | Specify the methods used to decide whether a study met the inclusion criteria of the review, including how many reviewers screened each record and each report retrieved, whether they worked independently, and if applicable, details of automation tools used in the process. | P6-P7 |
| Data collection process | 9 | Specify the methods used to collect data from reports, including how many reviewers collected data from each report, whether they worked independently, any processes for obtaining or confirming data from study investigators, and if applicable, details of automation tools used in the process. | P6-P8 |
| Data items | 10a | List and define all outcomes for which data were sought. Specify whether all results that were compatible with each outcome domain in each study were sought (e.g. for all measures, time points, analyses), and if not, the methods used to decide which results to collect. | P5-P7 |
|  | 10b | List and define all other variables for which data were sought (e.g. participant and intervention characteristics, funding sources). Describe any assumptions made about any missing or unclear information. | P5-P7 |
| Study risk of bias assessment | 11 | Specify the methods used to assess risk of bias in the included studies, including details of the tool(s) used, how many reviewers assessed each study and whether they worked independently, and if applicable, details of automation tools used in the process. | P6-P7 |
| Effect measures | 12 | Specify for each outcome the effect measure(s) (e.g. risk ratio, mean difference) used in the synthesis or presentation of results. | P7 |
| Synthesis methods | 13a | Describe the processes used to decide which studies were eligible for each synthesis (e.g. tabulating the study intervention characteristics and comparing against the planned groups for each synthesis (item #5)). | P5-P6 |
|  | 13b | Describe any methods required to prepare the data for presentation or synthesis, such as handling of missing summary statistics, or data conversions. | P7-P8 |
|  | 13c | Describe any methods used to tabulate or visually display results of individual studies and syntheses. | P8 |
|  | 13d | Describe any methods used to synthesize results and provide a rationale for the choice(s). If meta-analysis was performed, describe the model(s), method(s) to identify the presence and extent of statistical heterogeneity, and software package(s) used. | P7-P8 |
|  | 13e | Describe any methods used to explore possible causes of heterogeneity among study results (e.g. subgroup analysis, meta-regression). | P8 |
|  | 13f | Describe any sensitivity analyses conducted to assess robustness of the synthesized results. | P8 |
| Reporting bias assessment | 14 | Describe any methods used to assess risk of bias due to missing results in a synthesis (arising from reporting biases). | NA |
| Certainty assessment | 15 | Describe any methods used to assess certainty (or confidence) in the body of evidence for an outcome. | NA |
| **RESULTS** | | |  |
| Study selection | 16a | Describe the results of the search and selection process, from the number of records identified in the search to the number of studies included in the review, ideally using a flow diagram. | P8-P9 |
|  | 16b | Cite studies that might appear to meet the inclusion criteria, but which were excluded, and explain why they were excluded. | P8-P9 |
| Study characteristics | 17 | Cite each included study and present its characteristics. | P9 |
| Risk of bias in studies | 18 | Present assessments of risk of bias for each included study. | P9 |
| Results of individual studies | 19 | For all outcomes, present, for each study: (a) summary statistics for each group (where appropriate) and (b) an effect estimate and its precision (e.g. confidence/credible interval), ideally using structured tables or plots. | P9-P13 |
| Results of syntheses | 20a | For each synthesis, briefly summarise the characteristics and risk of bias among contributing studies. | P9 |
|  | 20b | Present results of all statistical syntheses conducted. If meta-analysis was done, present for each the summary estimate and its precision (e.g. confidence/credible interval) and measures of statistical heterogeneity. If comparing groups, describe the direction of the effect. | P9-P13 |
|  | 20c | Present results of all investigations of possible causes of heterogeneity among study results. | P14 |
|  | 20d | Present results of all sensitivity analyses conducted to assess the robustness of the synthesized results. | P18 |
| Reporting biases | 21 | Present assessments of risk of bias due to missing results (arising from reporting biases) for each synthesis assessed. | P14 |
| Certainty of evidence | 22 | Present assessments of certainty (or confidence) in the body of evidence for each outcome assessed. | P9-P13 |
| **DISCUSSION** | | |  |
| Discussion | 23a | Provide a general interpretation of the results in the context of other evidence. | P14-P19 |
|  | 23b | Discuss any limitations of the evidence included in the review. | P14-P19 |
|  | 23c | Discuss any limitations of the review processes used. | P14-P19 |
|  | 23d | Discuss implications of the results for practice, policy, and future research. | P14-P19 |
| **OTHER INFORMATION** | | |  |
| Registration and protocol | 24a | Provide registration information for the review, including register name and registration number, or state that the review was not registered. | P4 |
|  | 24b | Indicate where the review protocol can be accessed, or state that a protocol was not prepared. | NA |
|  | 24c | Describe and explain any amendments to information provided at registration or in the protocol. | NA |
| Support | 25 | Describe sources of financial or non-financial support for the review, and the role of the funders or sponsors in the review. | P20 |
| Competing interests | 26 | Declare any competing interests of review authors. | P20 |
| Availability of data, code and other materials | 27 | Report which of the following are publicly available and where they can be found: template data collection forms; data extracted from included studies; data used for all analyses; analytic code; any other materials used in the review. | Supplement |

# Table S2 Search strategies

A: PubMed, n=898

| **Search ID** | **Search Details** |
| --- | --- |
| #1 | ((((((((((((((((((((((((((((((((((((("Breast Neoplasms"[Mesh]) OR (Breast Neoplasm[Title/Abstract])) OR (Neoplasm, Breast[Title/Abstract])) OR (Breast Tumors[Title/Abstract])) OR (Breast Tumor[Title/Abstract])) OR (Tumor, Breast[Title/Abstract])) OR (Tumors, Breast[Title/Abstract])) OR (Neoplasms, Breast[Title/Abstract])) OR (Breast Cancer[Title/Abstract])) OR (Cancer, Breast[Title/Abstract])) OR (Mammary Cancer[Title/Abstract])) OR (Cancer, Mammary[Title/Abstract])) OR (Cancers, Mammary[Title/Abstract])) OR (Mammary Cancers[Title/Abstract])) OR (Malignant Neoplasm of Breast[Title/Abstract])) OR (Breast Malignant Neoplasm[Title/Abstract])) OR (Breast Malignant Neoplasms[Title/Abstract])) OR (Malignant Tumor of Breast[Title/Abstract])) OR (Breast Malignant Tumor[Title/Abstract])) OR (Breast Malignant Tumors[Title/Abstract])) OR (Cancer of Breast[Title/Abstract])) OR (Cancer of the Breast[Title/Abstract])) OR (Mammary Carcinoma, Human[Title/Abstract])) OR (Carcinoma, Human Mammary[Title/Abstract])) OR (Carcinomas, Human Mammary[Title/Abstract])) OR (Human Mammary Carcinomas[Title/Abstract])) OR (Mammary Carcinomas, Human[Title/Abstract])) OR (Human Mammary Carcinoma[Title/Abstract])) OR (Mammary Neoplasms, Human[Title/Abstract])) OR (Human Mammary Neoplasm[Title/Abstract])) OR (Human Mammary Neoplasms[Title/Abstract])) OR (Neoplasm, Human Mammary[Title/Abstract])) OR (Neoplasms, Human Mammary[Title/Abstract])) OR (Mammary Neoplasm, Human[Title/Abstract])) OR (Breast Carcinoma[Title/Abstract])) OR (Breast Carcinomas[Title/Abstract])) OR (Carcinoma, Breast[Title/Abstract])) OR (Carcinomas, Breast[Title/Abstract]) |
| #2 | ("metastasis"[Title/Abstract] OR "metastatic"[Title/Abstract] OR "advanced"[Title/Abstract] OR "secondary"[Title/Abstract] OR "recurrent"[Title/Abstract] OR "inoperable"[Title/Abstract] OR "unresectable"[Title/Abstract] OR "disseminated"[Title/Abstract] OR "incurable"[Title/Abstract]) |
| #3 | #1 AND #2 |
| #4 | ((((((((((("ERBB2 protein, human" [Supplementary Concept]) OR (v-erb-b2 erythroblastic leukemia viral oncogene homolog 2 protein, human[Title/Abstract])) OR (HER2 protein, human[Title/Abstract])) OR (Neu protein, human[Title/Abstract])) OR (EGFR2 protein, human[Title/Abstract])) OR (HER-2 protein, human[Title/Abstract])) OR (HER2delta16 protein, human[Title/Abstract])) OR (epidermal growth factor receptor 2, human[Title/Abstract])) OR (human epidermal growth factor receptor 2[Title/Abstract])) OR (HER-2[Title/Abstract])) OR (HER2[Title/Abstract])) OR (ERBB2[Title/Abstract]) |
| #5 | (((negative[Title/Abstract]) OR (low expressing[Title/Abstract])) OR (low expressed[Title/Abstract])) OR (-[Title/Abstract]) |
| #6 | (((((hormone receptor[Title/Abstract]) OR (estrogen receptor[Title/Abstract])) OR (Progesterone receptor[Title/Abstract])) OR (HR[Title/Abstract])) OR (ER[Title/Abstract])) OR (PR[Title/Abstract]) |
| #7 | ((((positive[Title/Abstract]) OR (enriched[Title/Abstract])) OR (overexpressing[Title/Abstract])) OR (overexpressed[Title/Abstract]))) OR (+[Title/Abstract]) |
| #8 | (#4 AND #5) AND (#6 AND #7) |
| #9 | ((Meta-Analysis[Publication Type]) OR (Review[Publication Type])) OR (systematic review[Publication Type]) |
| #10 | ("randomized controlled trial"[Publication Type] OR "Controlled Clinical Trial"[Publication Type] OR "Clinical Trial"[Publication Type] OR "Clinical Trials as Topic"[MeSH Terms] OR "clinical trials, phase iii as topic"[MeSH Terms] OR "clinical trials, phase iv as topic"[MeSH Terms] OR "Controlled Clinical Trials as Topic"[MeSH Terms] OR "Clinical Trial"[Publication Type] OR "Controlled Clinical Trial"[Publication Type] OR "clinical trial, phase iii"[Publication Type] OR "clinical trial, phase iv"[Publication Type] OR "Multicenter Study"[Publication Type] OR "Multicenter Studies as Topic"[MeSH Terms] OR "Random Allocation"[MeSH Terms] OR "Double-Blind Method"[MeSH Terms] OR "Single-Blind Method"[MeSH Terms] OR "Cross-Over Studies"[MeSH Terms] OR "Placebos"[MeSH Terms] OR ("Controlled"[Title/Abstract] AND ("Trial"[Title/Abstract] OR "trials"[Title/Abstract] OR "study"[Title/Abstract] OR "studies"[Title/Abstract])) OR ("blind"[Title/Abstract] OR "blinding"[Title/Abstract] OR "blinded"[Title/Abstract] OR "mask"[Title/Abstract] OR "masking"[Title/Abstract] OR "masked"[Title/Abstract] OR "placebo"[Title/Abstract] OR "Placebos"[Title/Abstract] OR "rct"[Title/Abstract] OR "random"[Title/Abstract] OR "randomised"[Title/Abstract]OR"Randomized"[Title/Abstract]OR "randomly"[Title/Abstract]OR"randomisation"[Title/Abstract]OR "randomization"[Title/Abstract]) OR "factorial"[Title/Abstract] OR ("divided"[Title/Abstract] AND ("group"[Title/Abstract] OR "groups"[Title/Abstract])) OR "crossover"[Title/Abstract] OR "cross over"[Title/Abstract] OR ("multicentre"[Title/Abstract] OR "multicentred"[Title/Abstract] OR "multicentric"[Title/Abstract]) OR ("versus"[Title] OR "vs"[Title]) OR "treatment arm"[Title/Abstract] OR ("phase III"[Title/Abstract] OR "phase three"[Title/Abstract] OR "phase 3"[Title/Abstract]) OR "latin square"[Title/Abstract]) NOT (("Animals"[MeSH Terms] OR "mouse"[Title] OR "mice"[Title] OR "pig"[Title] OR "pigs"[Title] OR "rat"[Title] OR "rats"[Title] OR "rabbit*"[Title]) NOT (("Animals"[MeSH Terms] OR "mouse"[Title] OR "mice"[Title] OR "pig"[Title] OR "pigs"[Title] OR "rat"[Title] OR "rats"[Title] OR "rabbit*"[Title] OR "cadaver"[Title] OR "cadavers"[Title]) AND "Humans"[MeSH Terms]) |
| #11 | ("2007/11/30"[Date - Publication] : "2022/11/30"[Date - Publication]) |
| #12 | #3 AND #8 AND #10 AND #11 |
| #13 | #12 NOT #9 |

B: Embase, n=393

| **Search ID** | **Search Details** |
| --- | --- |
| #1 | ('breast':ab,ti OR 'mammary':ab,ti) AND ('cancer':ab,ti OR 'neoplasm':ab,ti OR 'oncology':ab,ti OR 'tumor':ab,ti OR 'malignancy':ab,ti OR 'carcinoma':ab,ti OR 'adenocarcinoma':ab,ti OR 'sarcoma':ab,ti) |
| #2 | ('metastasis':ab,ti OR 'metastatic':ab,ti OR 'advanced':ab,ti OR 'secondary':ab,ti OR 'recurrent':ab,ti OR 'inoperable':ab,ti OR 'unresectable':ab,ti OR 'disseminated':ab,ti OR 'incurable':ab,ti) |
| #3 | #1 AND #2 |
| #4 | ('erbb2 protein, human':ab,ti OR 'v-erb-b2 erythroblastic leukemia viral oncogene homolog 2 protein, human':ab,ti OR 'her2 protein, human':ab,ti OR 'human epidermal growth factor receptor 2':ab,ti OR 'epidermal growth factor receptor 2, human':ab,ti OR 'her-2':ab,ti OR 'her2':ab,ti) |
| #5 | ('negative':ab,ti OR 'low expressing':ab,ti OR 'low expressed':ab,ti OR '-':ab,ti) |
| #6 | ('hormone receptor':ab,ti OR 'estrogen receptor':ab,ti OR 'progesterone receptor':ab,ti OR 'hr':ab,ti OR 'er':ab,ti OR 'pr':ab,ti) |
| #7 | ('positive':ab,ti OR 'enriched':ab,ti OR 'overexpressing':ab,ti OR 'overexpressed':ab,ti OR '+':ab,ti) |
| #8 | (#4 AND #5) AND (#6 AND #7) |
| #9 | ('randomized':ab,ti OR 'randomised':ab,ti OR 'randomly':ab,ti) |
| #10 | ('trial':ab,ti OR 'study':ab,ti) |
| #11 | #9 AND #10 |
| #12 | #3 AND #8 AND #11 |

# Table S3 Constant proportional risk test (probability) for Kaplan-Meier curves

A: First-line

| First-line PFS | | | First-line OS | | |
| --- | --- | --- | --- | --- | --- |
| Number | Clinical trial | Probability | Number | Clinical trial | Probability |
| 1 | PALOMA-2 | 0.3375 | 1 | PALOMA-2 | 0.8669 |
| 2 | PALOMA-4 | 0.1612 | 2 | PALOMA-1 | 0.8624 |
| 3 | PALOMA-1 | 0.0584 | 3 | MONALEESA-2 | 0.2091 |
| 4 | MONALEESA-2 | 0.6543 | 4 | Alliance CALGB40503 | 0.8191 |
| 5 | MONALEESA-3 | 0.3684 | 5 | MONARCH-3 | 0.1649 |
| 6 | MONARCH-2 | 0.1751 | 6 | FALCON | 0.2033 |
| 7 | MONARCH-3 | 0.4889 | 7 | SWOG S0226 | 0.6456 |
| 8 | PARSIFAL | 0.5332 | 8 | LEA | 0.2900 |
| 9 | FLIPPER | 0.3232 | 9 | PARSIFAL | 0.5794 |
| 10 | MONARCH plus A | 0.7264 | 10 | MONALEESA-3 | 0.4108 |
| 11 | DAWNA-2 | 0.6010 | 11 | MONARCH-2 | 0.9233 |
| 12 | SWOG S0226 | 0.0583 |  |  |  |
| 13 | FALCON | 0.4929 |  |  |  |
| 14 | LEA | 0.0082 |  |  |  |
| 15 | MINT | 0.4781 |  |  |  |
| 16 | Hiroji Iwata | 0.8753 |  |  |  |
| 17 | Alliance CALGB40503 | 0.091 |  |  |  |

B: Second/further-lines

| Second/further-lines PFS | | | Second/further-lines OS | | |
| --- | --- | --- | --- | --- | --- |
| Number | Clinical trial | Probability | Number | Clinical trial | Probability |
| 1 | PALOMA-3 | 0.0210 | 1 | PALOMA-3 | 0.9750 |
| 2 | MONALEESA-3 | 0.7411 | 2 | MONALEESA-3 | 0.9394 |
| 3 | MONARCH-2 | 0.7622 | 3 | MONARCH-2 | 0.0064 |
| 4 | MONARCH plus B | 0.0013 | 4 | PrE0102 | 0.3976 |
| 5 | DAWNA-1 | 0.0006 | 5 | SOLAR-1 | 0.2435 |
| 6 | MANTA | 0.0081 | 6 | FAKTION | 0.6071 |
| 7 | PrE0102 | 0.0081 | 7 | BELLE-2 | 0.8175 |
| 8 | PACE | 0.9852 | 8 | EMERALD | 0.1097 |
| 9 | BELLE-3 | 0.1206 | 9 | Alliance CALGB 40302 | 0.5836 |
| 10 | SOLAR-1 | 0.7253 | 10 | PACE | 0.3173 |
| 11 | FAKTION | 0.3694 | 11 | BOLERO-2 | 0.1896 |
| 12 | BELLE-2 | 0.0002 | 12 | BOLERO-6 | 0.8069 |
| 13 | FERGI | 0.7395 | 13 | E2112 | 0.0475 |
| 14 | NCT02756364 | 0.0027 | 14 | ENCORE301 | 0.9851 |
| 15 | SANDPIPER | 0.0003 | 15 | PEARL | 0.5292 |
| 16 | EMERALD | 0.6302 |  |  |  |
| 17 | MAINTAIN(1) | 0.7800 |  |  |  |
| 18 | EFECT | 0.6097 |  |  |  |
| 19 | Alliance CALGB 40302 | 0.1012 |  |  |  |
| 20 | NCT01142401 | 0.4337 |  |  |  |
| 21 | SERENA-2 | 0.5702 |  |  |  |
| 22 | BOLERO-2 | 0.0002 |  |  |  |
| 23 | ACE | 0.0166 |  |  |  |
| 24 | E2112 | 0.0135 |  |  |  |
| 25 | ENCORE301 | 0.1969 |  |  |  |
| 26 | MAINTAIN(2) | 0.6504 |  |  |  |
| 27 | BOLERO-6 | 0.9735 |  |  |  |
| 28 | PEARL | 0.0477 |  |  |  |

# Table S4 Baseline characteristics of included studies

| Trial information | Target population | Therapy line | Sample size | Median age | Intervention arm | Control arm | Region | HR(95% CI) | | Median follow-up | ORR |
| --- | --- | --- | --- | --- | --- | --- | --- | --- | --- | --- | --- |
|  |  |  |  |  |  |  |  | PFS | OS |  |  |
| PALOMA-3[33-36] | Postmenopausal (79.3%) and premenopausal or perimenopausal women (20.7%) with hormone-receptor-positive, HER2-negative advanced breast cancer, relapsed or progressed post-endocrine therapy. | 2nd line | 347 VS 174 | 56.9 VS 56.8 | Palbociclib/Fulvestrant (CDK4/6i/SERD) | Fulvestrant (SERD) | global | 0.42(0.32-0.56) | 0.806(0.65-0.99) | 73.3m | 10.37% VS 6.32% |
| PALOMA-2[37-39] | Women with ER-positive, HER2-negative advanced breast cancer, treatment-naïve for advanced disease. Postmenopausal status confirmed by bilateral oophorectomy, amenorrhea for ≥12 months, or hormone levels within postmenopausal ranges. | 1st line | 444 VS 222 | 61.7 VS 60.6 | Palbociclib/Letrozole (CDK4/6i/AI) | Letrozole (AI) | global | 0.563(0.461-0.687) | 0.95(0.78-1.78) | 38m | 42.11% VS 34.68% |
| PALOMA-4[40] | Postmenopausal Asian women diagnosed with ER+ advanced breast cancer, not indicated for chemotherapy, without prior metastatic systemic treatment. | 1st line | 169 VS 171 | 53.8 VS 53.7 | Palbociclib/Letrozole (CDK4/6i/AI) | Letrozole (AI) | asia | 0.68(0.53-0.87) | NA | 52.8m | 37.28% VS 31.58% |
| PALOMA-1/TRIO-18[41,42] | Postmenopausal women with ER-positive, HER2-negative advanced breast cancer, treatment-naïve for advanced disease. | 1st line | 84 VS 81 | 63.0 VS 64.0 | Palbociclib/Letrozole (CDK4/6i/AI) | Letrozole (AI) | global | 0.488(0.319-0.748) | 0.897(0.623-1.294) | 64.7m | 43.37% VS 35.06% |
| MONALEESA-2[43-45] | Postmenopausal women with locally confirmed HR-positive, HER2-negative recurrent or metastatic breast cancer, treatment-naive for advanced disease. | 1st line | 334 VS 334 | 61.4 VS 61.9 | Ribociclib/Letrozole (CDK4/6i/AI) | Letrozole (AI) | global | 0.568(0.457-0.704) | 0.76(0.63-0.93) | 80m | 40.42% VS 27.24% |
| MONALEESA-3[46,47] | Postmenopausal women and men with histologically or cytologically confirmed HR-positive/HER2-negative advanced breast cancer, untreated previously. | 1st line | 237 VS 128 | 63.4 VS 62.8 | Ribociclib/Fulvestrant (CDK4/6i/SERD) | Fulvestrant (SERD) | global | 0.55(0.42-0.72) | 0.67(0.5-0.9) | 70.8m | 32.40% VS 21.50% |
| MONALEESA-3[46,47] | Postmenopausal women and men with confirmed HR-positive/HER2-negative advanced breast cancer, histologically or cytologically. | 2nd line | 237 VS 110 | 63.4 VS 62.8 | Ribociclib/Fulvestrant (CDK4/6i/SERD) | Fulvestrant (SERD) | global | 0.57(0.44-0.74) | 0.78(0.587-1.037) | 70.8m | 32.40% VS 21.50% |
| MONARCH-2[48,49] | Patients with HR-positive/HER2-negative advanced breast cancer that progressed during/after neoadjuvant or adjuvant ET within 12 months, or during ET for ABC. Postmenopausal (83.2%), premenopausal or perimenopausal (16.1%). | 2nd line | 446 VS 223 | 59.3 VS 61.1 | Abemaciclib/Fulvestrant (CDK4/6i/SERD) | Fulvestrant (SERD) | global | 0.478(0.357-0.639) | 0.656(0.461-0.935) | 53.5m | 35.20% VS 16.14% |
| MONARCH-2[48,49] | The same with MONARCH-2, there is a separate first-line survival curve. | 1st line | 265 VS 133 | 60.2 VS 61.3 | Abemaciclib/Fulvestrant (CDK4/6i/SERD) | Fulvestrant (SERD) | global | 0.573(0.451-0.727) | 0.851(0.638-1.135) | 47.7m | 35.20% VS 16.14% |
| MONARCH-3[50-52] | Postmenopausal women with HR-positive, HER2-negative advanced breast cancer who had no prior systemic therapy in the advanced setting. | 1st line | 328 VS 165 | 63.1 VS 62.9 | Abemaciclib+Letrozole (CDK4/6i/AI) | Letrozole (AI) | global | 0.518(0.415-0.648) | 0.754(0.584-0.974) | 47.7m | 60.98% VS 45.45% |
| PARSIFAL[53] | Patients with hormone receptor-positive, ERBB2-negative advanced breast cancer, treatment-naive. Postmenopausal (91.8%). | 1st line | 243 VS 243 | 64.0 VS 62.0 | Palbociclib/Fulvestrant (CDK4/6i/SERD) | Palbociclib/Letrozole (CDK4/6i/AI) | global | 1.13(0.89-1.45) | 1.00(0.68-1.48) | 70.2m | 46.50% VS 57.28% |
| FLIPPER[54] | Postmenopausal women with HR-positive, HER2-negative ABC with de novo metastatic disease. | 1st line | 94 VS 95 | 64.0 VS 64.0 | Palbociclib/Fulvestrant (CDK4/6i/SERD) | Fulvestrant (SERD) | global | 0.48(0.37-0.64) | NA | 36m | 68.25% VS 42.19% |
| MONARCH plus A[55,56] | Postmenopausal women with HR-positive, HER2-negative ABC with no prior systemic therapy in an advanced setting. (cohort A) | 1st line | 207 VS 99 | 54 VS 54 | Abemaciclib/Letrozole (CDK4/6i/AI) | Letrozole (AI) | asia | 0.499(0.346-0.719) | NA | 28.6m | 56.04% VS 30.30% |
| MONARCH plus B[55,56] | Postmenopausal women with HR-positive, HER2-negative ABC progression on prior ET. (cohort B) | 2nd line | 104 VS 53 | 60 VS 60 | Abemaciclib/Fulvestrant (CDK4/6i/SERD) | Fulvestrant (SERD) | asia | 0.376(0.240-0.588) | NA | 16m | 38.46% VS 7.55% |
| DAWNA-1[57,58] | Women aged 18 - 75, any menopausal status, with confirmed HR-positive, HER2-negative advanced or metastatic breast cancer, not suitable for curative surgery or radiotherapy, and not candidates for chemotherapy. Note: Only postmenopausal Kaplan-Meier curves were used. | 2nd line | 241 VS 120 | 50.7 VS 52.4 | Dalpiciclib/Fulvestrant (CDK4/6i/SERD) | Fulvestrant (SERD) | asia | 0.44(0.31-0.62) | NA | 16m | 30.29% VS 15.83% |
| DAWNA-2[59] | Patients aged 18–75, any menopausal status, ECOG performance status 0–1, with pathologically confirmed HR-positive, HER2-negative untreated advanced breast cancer. Note: A separate survival curve exists for postmenopausal patients. | 1st line | 303 VS 153 | 51 VS 50 | Dalpiciclib/Letrozole (CDK4/6i/AI) | Letrozole (AI) | asia | 0.51(0.38-0.69) | NA | 25.2m | 57.43% VS 47.71% |
| BOLERO-2[60,61] | Postmenopausal women with HR+/HER2-advanced BC with recurrence/progression after NSAIs. | 2nd line | 485 VS 239 | 62.5 VS 61.2 | Everolimus/Exemestane (mTORi/AI) | Exemestane (AI) | global | 0.38(0.31-0.48) | 0.89(0.73-1.1) | 21.7m | 12.58% VS 2.09% |
| MANTA(1)[62] | Postmenopausal women with ER-positive locally advanced or metastatic breast cancer who relapsed during or within 12 months post-adjuvant AI therapy, or progressed on AI treatment. | 2nd line | 65 VS 67 | 63 VS 63 | Fulvestrant/Everolimus (SERD/mTORi) | Fulvestrant (SERD) | global | 0.63(0.42-0.92) | 0.56(0.28-1.09) | 23.7m | 40.62% VS 25.76% |
| MANTA(2)[62] |  | 2nd line | 103 VS 67 | 63 VS 63 | Fulvestrant/daily Vistusertib (SERD/mTORi) | Fulvestrant (SERD) | global | 0.88(0.63-1.24) | NA | 23.7m | 31.68% VS 25.76% |
| MANTA(3)[62] |  | 2nd line | 98 VS 67 | 64 VS 63 | Fulvestrant/intermittent Vistusertib (SERD/mTORi) | Fulvestrant (SERD) | global | 0.79(0.55-1.12) | NA | 23.7m | 28.42% VS 25.76% |
| PrE0102  [63] | Postmenopausal women with ER-positive, human epidermal growth factor receptor 2–negative, AI-resistant metastatic breast cancer. | 2nd line | 66 VS 65 | 64 VS 59 | Fulvestrant/Everolimus (SERD/mTORi) | Fulvestrant (SERD) | global | 0.63(0.42-0.92) | 1.31(0.72-2.38) | 18m | 18.18% VS 12.31% |
| BOLERO-6(1)[64] | Postmenopausal women with ER-positive, HER2-negative metastatic/recurrent breast cancer or inoperable locally advanced breast cancer, with relapse or progression on letrozole or anastrozole treatment. | 2nd line | 104 VS 103 | 61 VS 61 | Everolimus/Exemestane (mTORi/AI) | Everolimus (mTORi) | global | 0.74(0.57-0.97) | 1.27(0.95-1.70) | NA | 20.19% VS 11.65% |
| BOLERO-6(2)[64] |  | 2nd line | 104 VS 102 | 61 VS 60 | Everolimus/Exemestane (mTORi/AI) | Capecitabine (single-agent chemotherapy) | global | 1.26(0.96-1.66) | 1.33(0.99-1.79) | NA | 20.19% VS 22.55% |
| SWOG S0226  [65,66] | Postmenopausal women with HR-positive, previously untreated metastatic breast cancer, either estrogen receptor-positive, progesterone-receptor-positive, or both, and predominantly HER2-negative (90.5%). | 1st line | 345 VS 349 | 65 VS 65 | Anastrozole (AI) | Fulvestrant/Anastrozole (SERD/AI) | global | 0.80(0.68-0.94) | 0.82(0.69-0.98) | 37.6m | 22.03% VS 26.93% |
| FALCON[67] | Postmenopausal women with histologically confirmed hormone receptor-positive (estrogen receptor-positive, progesterone receptor-positive, or both) and HER2-negative locally advanced or metastatic breast cancer. | 1st line | 230 VS 232 | 63.8 VS 63.3 | Fulvestrant (SERD) | Anastrozole (AI) | global | 0.797(0.637-0.999) | 0.7(0.5-0.98) | 37.9m | 46.11% VS 44.90% |
| BELLE-3[68] | Postmenopausal women (≥18 years) with confirmed HR-positive, HER2-negative advanced or metastatic breast cancer, previously treated with aromatase inhibitors and resistant to endocrine therapy and mTOR inhibitors. | 2nd line | 289 VS 143 | 60.5 VS 61.5 | Buparlisib/Fulvestrant (PI3Ki/SERD) | Fulvestrant (SERD) | global | 0.67(0.53-0.84) | NA | NA | 7.61% VS 2.10% |
| SOLAR-1  [69,70] | Men and postmenopausal women (99% postmenopausal) with locally confirmed HR-positive, HER2-negative advanced breast cancer, eligible for further endocrine therapy post-relapse or progression. | 2nd line | 284 VS 288 | 62.6 VS 63.3 | Alpelisib/Fulvestrant (PI3Ki/SERD) | Fulvestrant (SERD) | global | 0.85(0.58-1.25) | 0.86(0.64-1.15) | 16.3m | 26.41% VS 12.89% |
| FAKTION[71] | Postmenopausal women (≥18 years) with local investigator-confirmed ER-positive, HER2-negative metastatic breast cancer or inoperable locally advanced breast cancer. | 2nd line | 69 VS 71 | 60 VS 62 | Capivasertib+Fulvestrant (AKTi/SERD) | Fulvestrant (SERD) | europe | 0.56(0.38-0.81) | 0.66(0.45-0.97) | 20m | 28.99% VS 8.45% |
| ACE[72] | Postmenopausal women with histologically or cytologically confirmed hormone receptor-positive, HER2-negative, inoperable breast cancer with relapse or progression post at least one endocrine therapy. | 2nd line | 244 VS 121 | 55 VS 55 | Tucidinostat/Exemestane (HDACi/AI) | Exemestane (AI) | asia | 0.75(0.58-0.98) | NA | 60.9m | 16.39% VS 7.44% |
| BELLE-2[73,74] | Postmenopausal women aged 18 years or older with a histologically or cytologically confirmed diagnosis of hormone receptor-positive and HER2-negative breast cancer. | 2nd line | 576 VS 571 | 62.2 VS 60.6 | Buparlisib/Fulvestrant (PI3Ki/SERD) | Fulvestrant (SERD) | global | 0.78(0.67-0.89) | 0.87(0.74-1.02) | 13.9m | 11.81% VS 7.71% |
| FERGI  [75] | Postmenopausal women aged 18 years or older with oestrogen receptor-positive, HER2-negative locally advanced or metastatic breast cancer appropriate for endocrine therapy. | 2nd line | 89 VS 79 | 60 VS 63 | Pictilisib/Fulvestrant (PI3Ki/SERD) | Fulvestrant (SERD) | global | 0.74(0.52-1.06) | NA | 13.73m | 7.87% VS 6.33% |
| LEA[76] | First-line therapy for postmenopausal patients with HER2-negative and hormone receptor-positive advanced breast cancer. | 1st line | 184 VS 190 | 66 VS 64 | Bevacizumab/Letrozole (Anti-VEGF/AI) | Letrozole (AI) | europe | 0.83(0.65-1.06) | 0.87(0.58-1.32) | 17.5m | 21.74% VS 40.53% |
| NCT02756364(1)  [77] | Postmenopausal women with histologically confirmed ER+ (≥1% positive stained cells)/HER2- metastatic or advanced breast cancer, not candidates for curative resection or radiotherapy. | 2nd line | 47 VS 46 | 59.0 VS 60.0 | Fulvestrant/Sapanisertib4mg/d (SERD/mTORi) | Fulvestrant (SERD) | global | 0.77(0.47-1.26) | 0.71(0.36-1.4) | NA | 21.28% VS 10.87% |
| NCT02756364(2)  [77] |  | 2nd line | 47 VS 46 | 57.0 VS 60.0 | Fulvestrant/Sapanisertib30mg/w (SERD/mTORi) | Fulvestrant (SERD) | global | 0.88(0.53-1.45) | 0.89(0.47-1.68) | NA | 12.77% VS 10.87% |
| SANDPIPER[78] | Female, postmenopausal with histologically or cytologically confirmed invasive, ER-positive, HER2-negative metastatic or inoperable locally advanced breast cancer. | 2nd line | 340 VS 176 | 60.1 VS 60.7 | Taselisib+Fulvestrant (PI3Ki/SERD) | Fulvestrant (SERD) | global | 0.7(0.56-0.89) | 0.85(0.58-1.25) | NA | 19.47% VS 14.08% |
| EMERALD[79] | Postmenopausal women or men aged ≥18 years with histologically or cytologically proven ER-positive/HER2-negative breast adenocarcinoma, presenting with locoregional recurrence. | 2nd line | 239 VS 238 | 63 VS 64 | Elacestrant (SERD) | Fulvestrant (SERD) | global | 0.7(0.55-0.88) | 0.75(0.54-1.04) | NA | NA |
| E2112  [80] | Men or postmenopausal women (94.1%) with advanced HR-positive, HER2-negative breast cancer, with disease progression post-nonsteroidal AI treatment. | 2nd line | 305 VS 303 | 63 VS 63 | Entinostat/Exemestane (HDACi/AI) | Exemestane (AI) | global | 0.87(0.67-1.13) | 0.99(0.82-1.21) | 22m | 5.79% VS 5.65% |
| ENCORE301  [81] | Postmenopausal women with locally determined ER-positive breast cancer, relapsing or progressing on nonsteroidal aromatase inhibitor therapy. HER2-negative status in 89% of cases. | 2nd line | 64 VS 66 | 63 VS 62 | Entinostat/Exemestane (HDACi/AI) | Exemestane (AI) | global | 0.73(0.50-1.07) | 0.59(0.36-0.97) | 16m | 6.25% VS 4.55% |
| MINT(1)  [82] | Postmenopausal female patients with histologically or cytologically confirmed breast cancer, presenting with locally advanced or metastatic disease, ER and/or PR positive, HER2 negative. | 1st line | 120 VS 121 | 60 VS 61 | Sapitinib40mg/Anastrozole (EGFRi/AI) | Anastrozole (AI) | global | 1.37(0.91-2.06) | NA | NA | 30% VS 24.79% |
| MINT(2)  [82] |  | 1st line | 118 VS 121 | 62 VS 61 | Sapitinib20mg/Anastrozole (EGFRi/AI) | Anastrozole (AI) | global | 1.16(0.77-1.75) | NA |  | 24.58% VS 24.79% |
| Hiroji Iwata2013[83] | Postmenopausal patients with metastatic, progressive, or locally recurrent, inoperable, hormone-receptor-positive breast cancer, confirmed histologically or cytologically at primary diagnosis or upon metastasis detection. HER2 score <3 in 93.6% of cases. | 1st line | 149 vs 149 | 63.4 vs 64 | Exemestane (AI) | Anastrozole (AI) | Japan | 1.007(0.771-1.317) | 1.062(0.733-1.539) | NA | 43.94% VS 39.06% |
| Alliance CALGB40503[84] | Women aged 18 years or older, postmenopausal, with locally advanced, unresectable, or metastatic hormone receptor positive breast cancer. HER2 negative status in 89% of cases. | 1st line | 173 vs 170 | 56 vs 59 | Bevacizumab/Letrozole (Anti-VEGF/AI) | Letrozole (AI) | global | 0.75(0.59-0.96) | 0.87(0.65-1.18) | 66.4w | 68.79% VS 48.82% |
| EFECT  [85] | Postmenopausal women with incurable locally advanced or metastatic breast cancer that relapsed during treatment with a nonsteroidal aromatase inhibitor (NSAI), or within 6 months of discontinuing adjuvant NSAI therapy, or whose advanced disease progressed while on NSAI treatment. | 2nd line | 351 vs 342 | 63 vs 63 | Fulvestrant (SERD) | Exemestane (AI) | global | 0.963(0.819-1.133) | NA | NA | 7.41% VS 6.73% |
| Alliance CALGB 40302  [86] | Postmenopausal women with stage III or IV breast cancer deemed unamenable to curative therapy, predominantly HER2-negative (81%), with a separate Kaplan-Meier (KM) survival curve for human epidermal growth factor receptor 2–negative cases. | 2nd line | 146 vs 145 | NA | Lapatinib/Fulvestrant (EGFRi/SERD) | Fulvestrant (SERD) | global | 1.00(0.76-1.30) | 0.91(0.68-1.21) | NA | 19.80% VS 8.82% |
| NCT01142401[87] | Postmenopausal women with histologically or cytologically confirmed unresectable locally advanced or metastatic, ER-positive, HER2/neu-negative breast cancer. Patients required to have AI-resistant disease, defined as relapse while on adjuvant AI therapy and/or disease progression after one or more AIs for metastatic disease. | 2nd line | 59 VS 57 | 57 VS 59 | Fulvestrant/Bortezomib (SERD/Pi) | Fulvestrant (AI) | United States | 0.73(0.49-1.09) | NA | 2.8y | NA |
| MAINTAIN(1)[88] | Postmenopausal patients with HR+/HER2- MBC whose cancer progressed during ET and CDK4/6i, participants switched ET. | 2nd line | 49 VS 50 | NA | Ribociclib/Fulvestrant (CDK4/6i/SERD) | Fulvestrant (SERD) | global | 0.60(0.39-0.94) | NA | NA | NA |
| MAINTAIN(2)[88] |  | 2nd line | 11 VS 9 | NA | Ribociclib/Exemestane (CDK4/6i/AI) | Exemestane (AI) | global | 0.41(0.14-1.24) | NA | NA | NA |
| PACE(1)  [89,90] | Participants with histologically confirmed hormone receptor-positive (HR+), HER2-negative metastatic or locally recurrent unresectable invasive breast cancer. Overall, 80.9% were postmenopausal. | 2nd line | 111 VS 55 | NA | Palbociclib/Fulvestrant (CDK4/6i/SERD) | Fulvestrant (SERD) | global | 1.11(0.74-1.66) | 1.02(0.67-1.56) | NA | 13.70% VS 10.81% |
| PACE(2)  [89,90] |  | 2nd line | 54 VS 55 | NA | Palbociclib/Fulvestrant/Avelumab  (CDK4/6i/SERD/ICI) | Fulvestrant (SERD) | global | 0.75(0.47-1.20) | 0.68(0.40-1.15) | NA | 17.95% VS 10.81% |
| SERENA-2(1)  [91] | Postmenopausal women with ER+/HER2- advanced breast cancer (ABC) who are candidates to receive fulvestrant monotherapy in the advanced setting. | 2nd line | 74 VS 73 | NA | Camizestrant75mg (SERD) | Fulvestrant (SERD) | global | 0.58(0.41-0.81) | NA | NA | NA |
| SERENA-2(2)  [91] |  | 2nd line | 73 VS 73 | NA | Camizestrant150mg (SERD) | Fulvestrant (SERD) | global | 0.67(0.48-0.92) | NA | NA | NA |
| PEARL  [92,93] | Postmenopausal women with hormone receptor-positive/HER2-negative MBC resistant to previous AIs. | 2nd line | 149 VS 156 | 62 VS 60 | Palbociclib+Fulvestrant (CDK4/6i/SERD) | Capecitabine (single-agent chemotherapy) | global | 1.13(0.85-1.50) | 1.05(0.78-1.42) | NA | 22.62% VS 33.33% |

Abbreviations: AI, Aromatase inhibitor; AKTi, AKT inhibitors; Anti-VEGF, anti-vascular endothelial growth factor; CDK4/6i, Cyclin-dependent kinase 4 and 6 inhibitors; EGFRi: Epidermal Growth Factor Receptor inhibitor; HDACi, Histone deacetylase inhibitor; ICI, Immune checkpoint inhibitor; mTORi, Mammalian target of rapamycin inhibitor; Pi, Protease inhibitor; PI3Ki, Phosphatidylinositol 3‐kinase inhibitor; SERD, Selective Estrogen Receptor Degrader.

Note: References refer to the citation numbers in the main text.

# Table S5 Summary of adverse events in RCTs across various dimensions

| Trial information | Intervention arm | Control arm | AEs/Total | Grade 3-5 AEs rate(%) | AEs leading to discontinuation | AEs leading to death |
| --- | --- | --- | --- | --- | --- | --- |
| PALOMA-3[33-36] | Palbociclib/Fulvestrant (CDK4/6i/SERD) | Fulvestrant (SERD) | 97.68% VS 88.95% | 69.27% VS 18.02% | NA | NA |
| PALOMA-2[37-39] | Palbociclib/Letrozole (CDK4/6i/AI) | Letrozole (AI) | 98.87% VS 95.49% | 75.68% VS 24.32% | 12.16% VS 5.86% | 2.25% VS 1.80% |
| PALOMA-4[40] | Palbociclib/Letrozole (CDK4/6i/AI) | Letrozole (AI) | 99.41% VS 92.26% | NA | 7.69% VS 3.0% | NA |
| PALOMA-1/TRIO-18[41,42] | Palbociclib/Letrozole (CDK4/6i/AI) | Letrozole (AI) | 98.80% VS 84.41% | 75.90% VS 20.78% | 13.25% VS 2.60% | NA |
| MONALEESA-2[43-45] | Ribociclib/Letrozole (CDK4/6i/AI) | Letrozole (AI) | 98.50% VS 96.97% | 81.14% VS 32.73% | 8.10% VS 2.40% | NA |
| MONALEESA-3[46,47] | Ribociclib/Fulvestrant (CDK4/6i/SERD) | Fulvestrant (SERD) | 69.57% VS 2.07% | 53.41% VS 4.15% | NA | NA |
| MONALEESA-3[46,47] | Ribociclib/Fulvestrant (CDK4/6i/SERD) | Fulvestrant (SERD) | 69.57% VS 2.07% | 53.41% VS 4.15% | NA | NA |
| MONARCH-2[48,49] | Abemaciclib/Fulvestrant (CDK4/6i/SERD) | Fulvestrant (SERD) | 97.63% VS 90.70% | 69.82% VS 29.07% | 13.50% VS 2.30% | NA |
| MONARCH-2[48,49] | Abemaciclib/Fulvestrant (CDK4/6i/SERD) | Fulvestrant (SERD) | 99.24% VS 90.98% | 67.42% VS 26.31% | 20.00% VS 4.50% | NA |
| MONARCH-3[50-52] | Abemaciclib/Letrozole (CDK4/6i/AI) | Letrozole (AI) | 98.78% VS 94.41% | 55.35% VS 24.84% | 16.51% VS 3.31% | 3.36% VS 1.24% |
| PARSIFAL[53] | Palbociclib/Fulvestrant (CDK4/6i/SERD) | Palbociclib/Letrozole (CDK4/6i/AI) | 99.58% VS 99.17% | 80.91% VS 78.51% | 5.40% VS 2.10% | NA |
| FLIPPER[54] | Palbociclib/Fulvestrant (CDK4/6i/SERD) | Fulvestrant (SERD) | 100% VS 98.95% | 88.30% VS 38.95% | 4.26% VS 3.20% | 2.13% VS 1.05% |
| MONARCH plus A[55,56] | Abemaciclib/Letrozole (CDK4/6i/AI) | Letrozole (AI) | 99.51% VS 88.89% | 59.02% VS 23.23% | 10.70% VS 1.01% | NA |
| MONARCH plus B[55,56] | Abemaciclib/Fulvestrant (CDK4/6i/SERD) | Fulvestrant (SERD) | 99.04% VS 79.24% | 51.92% VS 15.09% | NA | NA |
| DAWNA-1[57,58] | Dalpiciclib/Fulvestrant (CDK4/6i/SERD) | Fulvestrant (SERD) | NA | 83.33% VS 13.33% | 2.50% VS 3.30% | 0.80% VS 3.33% |
| DAWNA-2[59] | Dalpiciclib/Letrozole (CDK4/6i/AI) | Letrozole (AI) | NA | NA | NA | NA |
| BOLERO-2[60,61] | Everolimus/Exemestane (mTORi/AI) | Exemestane (AI) | NA | 32.78% VS 15.55% | 29.04% VS 5.04% | 1.66% VS 0.42% |
| MANTA(1)[62] | Fulvestrant/Everolimus (SERD/mTORi) | Fulvestrant (SERD) | NA | 30.1% VS 0% | 7.81% VS 1.49% | NA |
| MANTA(2)[62] | Fulvestrant/daily Vistusertib (SERD/mTORi) | Fulvestrant (SERD) | NA | 54.5% VS 0% | 7.92% VS 1.49% | NA |
| MANTA(3)[62] | Fulvestrant/intermittent Vistusertib (SERD/mTORi) | Fulvestrant (SERD) | NA | 36.9% VS 0% | 4.21% VS 1.49% | NA |
| PrE0102  [63] | Fulvestrant/Everolimus (SERD/mTORi) | Fulvestrant (SERD) | NA | 45.31% VS 9.23% | 20.31% VS 7.69% | 6.25% VS 3.08% |
| BOLERO-6(1)[64] | Everolimus/Exemestane (mTORi/AI) | Everolimus (mTORi) | 100% VS 98.06% | 70.19% VS 59.22% | 17.31% VS 19.42% | 3.85% VS 5.83% |
| BOLERO-6(2)[64] | Everolimus/Exemestane (mTORi/AI) | Capecitabine (single-agent chemotherapy) | 100% VS 100% | 70.19% VS 73.53% | 17.31% VS 20.59% | 3.65% VS 4.90% |
| SWOG S0226  [65,66] | Anastrozole (AI) | Fulvestrant/Anastrozole (SERD/AI) | 100% VS 100% | 12.65% VS 13.87% | NA | NA |
| FALCON[67] | Fulvestrant (SERD) | Anastrozole (AI) | 23.76% VS 21.36% | NA | 2.97% VS 4.85% | 7.02% VS 4.74% |
| BELLE-3[68] | Buparlisib/Fulvestrant (PI3Ki/SERD) | Fulvestrant (SERD) | NA | 74.30% VS 15.00% | 7.68% VS 2.14% | NA |
| SOLAR-1  [69,70] | Alpelisib/Fulvestrant (PI3Ki/SERD) | Fulvestrant (SERD) | 99.29% VS 93.03% | 78.17% VS 37.28% | 25.00% VS 4.18% | NA |
| FAKTION[71] | Capivasertib/Fulvestrant (AKTi/SERD) | Fulvestrant (SERD) | NA | 65.22% VS 50.00% | 2.90% VS 1.43% | NA |
| ACE[72] | Tucidinostat/Exemestane (HDACi/AI) | Exemestane (AI) | 97.95% VS 89.26% | 16.80% VS 16.53% | 9.02% VS 4.13% | NA |
| BELLE-2[73,74] | Buparlisib/Fulvestrant (PI3Ki/SERD) | Fulvestrant (SERD) | 93.30% VS 93.33% | 78.01% VS 33.68% | 41.00% VS 7.00% | NA |
| FERGI  [75] | Pictilisib/Fulvestrant (PI3Ki/SERD) | Fulvestrant (SERD) | 31.46% VS 12.66% | 60.67% VS 27.85% | 22.47% VS 5.06% | NA |
| LEA[76] | Bevacizumab/Letrozole (Anti-VEGF/AI) | Letrozole (AI) | NA | NA | NA | NA |
| NCT02756364(1)  [77] | Fulvestrant/Sapanisertib4mg/d (SERD/mTORi) | Fulvestrant (SERD) | 100% VS 89.13% | 74.47% VS 30.43% | 31.20% VS 4.30% | NA |
| NCT02756364(2)  [77] | Fulvestrant/Sapanisertib30mg/w (SERD/mTORi) | Fulvestrant (SERD) | 100% VS 89.13% | 65.96% VS 30.43% | 36.17% VS 4.30% | NA |
| SANDPIPER[78] | Taselisib/Fulvestrant (PI3Ki/SERD) | Fulvestrant (SERD) | 95.43% VS 89.67% | 49.52% VS 16.43% | 16.83% VS 2.35% | NA |
| EMERALD[79] | Elacestrant (SERD) | Fulvestrant (SERD) | 91.98% VS 89.44% | 27.00% VS 20.50% | 3.38% VS 0.62% | NA |
| E2112  [80] | Entinostat/Exemestane (HDACi/AI) | Exemestane (AI) | NA | 51.00% VS 15.60% | 16.55% VS 7.85% | NA |
| ENCORE301  [81] | Entinostat/Exemestane (HDACi/AI) | Exemestane (AI) | 95.24% VS 84.85% | 49.21% VS 25.76% | 11.00% VS 2.00% | NA |
| MINT(1)  [82] | Sapitinib40mg/Anastrozole (EGFRi/AI) | Anastrozole (AI) | 96.67% VS 84.30% | 36.67% VS 14.88% | 8.33% VS 2.48% | NA |
| MINT(2)  [82] | Sapitinib20mg/Anastrozole (EGFRi/AI) | Anastrozole (AI) | 94.91% VS 84.30% | 18.64% VS 14.88% | 5.08% VS 2.48% | NA |
| Hiroji Iwata2013[83] | Exemestane (AI) | Anastrozole (AI) | 91.28% VS 87.92% | 18.79% VS 18.12% | 6.71% VS 6.04% | 2.01% VS 0.67% |
| Alliance CALGB40503[84] | Bevacizumab/Letrozole (Anti-VEGF/AI) | Letrozole (AI) | NA | 45.66% VS 13.53% | NA | NA |
| EFECT  [85] | Fulvestrant (SERD) | Exemestane (AI) | 55.27% VS 65.29% | NA | 19.08% VS 20.88% | NA |
| Alliance CALGB 40302  [86] | Lapatinib/Fulvestrant (EGFRi/SERD) | Fulvestrant (SERD) | NA | 19.18% VS 5.52% | NA | NA |
| NCT01142401[87] | Fulvestrant/Bortezomib (SERD/Pi) | Fulvestrant (AI) | NA | 28.07% VS 15.25% | NA | NA |
| MAINTAIN(1)[88] | Ribociclib/Fulvestrant (CDK4/6i/SERD) | Fulvestrant (SERD) | NA | NA | NA | NA |
| MAINTAIN(2)[88] | Ribociclib/Exemestane (CDK4/6i/AI) | Exemestane (AI) | NA | NA | NA | NA |
| PACE(1)  [89,90] | Palbociclib/Fulvestrant (CDK4/6i/SERD) | Fulvestrant (SERD) | NA | NA | NA | NA |
| PACE(2)  [89,90] | Palbociclib/Fulvestrant/Avelumab  (CDK4/6i/SERD/ICI) | Fulvestrant (SERD) | NA | NA | NA | NA |
| SERENA-2(1)  [91] | Camizestrant75mg (SERD) | Fulvestrant (SERD) | 77.03% VS 68.50% | NA | 14.86% VS 4.11% | NA |
| SERENA-2(2)  [91] | Camizestrant150mg (SERD) | Fulvestrant (SERD) | 90.41% VS 68.50% | NA | 21.92% VS 4.11% | NA |
| PEARL  [92,93] | Palbociclib/Fulvestrant (CDK4/6i/SERD) | Capecitabine (single-agent chemotherapy) | 85.91% VS 95.16% | 61.70% VS 46.71% | 8.05% VS 16.95% | 0.67% VS 1.38% |

Abbreviations: AI, Aromatase inhibitor; AKTi, AKT inhibitors; Anti-VEGF, Anti-vascular endothelial growth factor; CDK4/6i, Cyclin-dependent kinase 4 and 6 inhibitor; EGFRi: Epidermal Growth Factor Receptor inhibitor; HDACi, Histone deacetylase inhibitor; ICI, Immune checkpoint inhibitor; mTORi, Mammalian target of rapamycin inhibitor; Pi, Protease inhibitor; PI3Ki, Phosphatidylinositol 3‐kinase inhibitor; SERD, Selective Estrogen Receptor Degrader.

Note: References refer to the citation numbers in the main text.

# Tbale S6 List of drugs approved by the Food and Drug Administration

| Drug | Approval details |
| --- | --- |
| Palbociclib | - Palbociclib, in combination with an aromatase inhibitor or fulvestrant, is indicated for the treatment of hormone receptor (HR)-positive, human epidermal growth factor receptor 2 (HER2)-negative advanced or metastatic breast cancer in postmenopausal women. - As initial endocrine-based therapy, palbociclib is used in combination with letrozole for postmenopausal women with estrogen receptor (ER)-positive, HER2-negative advanced breast cancer. - Additionally, for women with HR-positive, HER2-negative advanced or metastatic breast cancer who have experienced disease progression following endocrine therapy, palbociclib is combined with fulvestrant. |
| Abemaciclib | Breast cancer that is hormone receptor positive and HER2 negative (HER2-). It is used: In adults whose cancer is advanced or has metastasized.   - With fulvestrant in adults whose cancer got worse after treatment with hormone therapy. - Alone in adults whose cancer got worse after treatment with hormone therapy and chemotherapy given for metastatic cancer. - With an aromatase inhibitor given as first-line hormone therapy in men and postmenopausal women |
| Ribociclib | - Combination with an Aromatase Inhibitor: This approach is for women with metastatic HR+ HER2- breast cancer who have not previously received hormone therapy. Aromatase inhibitors are commonly used in postmenopausal women to lower estrogen levels and slow the growth of hormone receptor-positive tumors. - Combination with Fulvestrant: This treatment is for postmenopausal women with metastatic HR+ HER2- breast cancer who either: Have not been treated with hormone therapy before, or have experienced disease progression during or after hormone therapy. |
| Alpelisib | - Breast cancer that is hormone receptor positive and HER2 negative and has a mutation in the PIK3CA gene. It is used with fulvestrant to treat postmenopausal women, and men, whose breast cancer is advanced or metastatic and has gotten worse during or after treatment with hormone therapy. |
| Everolimus | - Breast cancer. It is used in combination with exemestane in postmenopausal women with advanced hormone receptor–positive (HR+) breast cancer that is also HER2 negative (HER2-) and has not gotten better after treatment with letrozole or anastrozole |
| Anastrozole | - First-line Therapy for Locally Advanced or Metastatic HR+: When breast cancer is hormone receptor-positive (HR+) and has either locally advanced or metastasized, the specified treatment is used as the initial therapy. - First-line Therapy for Hormone Receptor Status Unknown: If the breast cancer is locally advanced or metastatic and the hormone receptor status is unknown—that is, it is not confirmed whether the cancer is hormone receptor-positive (HR+) or hormone receptor-negative (HR-)—the treatment is still employed as the first-line therapy. |
| Fulvestrant | - Monotherapy: Used alone for HR-positive, HER2-negative advanced breast cancer in postmenopausal women who have not previously been treated with hormone therapy. - Subsequent Monotherapy: Used alone in cases where HR-positive advanced cancer has worsened after hormone therapy. - Combination with Ribociclib Succinate: Used in combination for HR-positive, HER2-negative advanced or metastatic cancer as either first-line hormone therapy or after progression during hormone therapy. - Combination with Palbociclib or Abemaciclib: Administered in combination for HR-positive, HER2-negative advanced or metastatic cancer that has progressed following hormone therapy. |
| Letrozole | - First-line Treatment for HR+ Cancer: For locally advanced or metastatic breast cancer that is hormone receptor-positive (HR+), the recommended approach is to use the specified treatment as the first therapeutic option. - First-line Treatment for Unknown HR Status: If the breast cancer is locally advanced or metastatic but the hormone receptor status (HR+ or HR-) is not determined, the treatment is still used as the initial therapy. - Second-line Treatment After Antiestrogen Therapy: For cases where the breast cancer has progressed following antiestrogen therapy, the treatment is employed as a subsequent line of therapy. |
| Exemestane | - Advanced Breast Cancer: It is approved for the treatment of advanced-stage breast cancer, which typically refers to cancer that has spread beyond the breast and nearby lymph nodes to other parts of the body. - Early-Stage, Estrogen Receptor-Positive Breast Cancer: Exemestane is also approved for treating early-stage breast cancer that is estrogen receptor-positive (ER+), after the cancer has been treated with surgery. It may be used to reduce the risk of the cancer coming back. |
| Elacestrant | - Breast cancer that is estrogen receptor (ER) positive and HER2 negative, has a mutation (change) in the ESR1 gene, and has spread. It is used in postmenopausal women and in men whose cancer has gotten worse after at least one type of hormone therapy. |

# Table S7 Akaike Information Criterion value for survival curves from Fractional Polynomial models

| P1 value | First line PFS therapies | Second/further-lines PFS therapies | Second/further-lines OS therapies | First line PFS mechanisms | First line OS mechanisms | Second/further-lines PFS mechanisms | Second/further-lines OS mechanisms |
| --- | --- | --- | --- | --- | --- | --- | --- |
| -2 | 6026.30 | **7634.11** | 5110.56 | **5678.50** | 4242.12 | **6922.62** | 4941.55 |
| -1 | **6106.33** | 8012.07 | **5058.24** | 5751.88 | 4174.33 | 7267.66 | **4889.17** |
| -0.5 | 6144.88 | 8162.32 | 5067.25 | 5789.30 | **4154.74** | 7406.88 | 4894.63 |
| 0 | 6167.78 | 8237.94 | 5123.49 | 5813.52 | 4182.07 | 7477.57 | 4944.51 |
| 0.5 | 6174.39 | 8242.03 | 5205.60 | 5823.12 | 4246.42 | 7481.56 | 5019.13 |
| 1 | 6173.81 | 8216.66 | 5281.13 | 5825.49 | 4312.91 | 7458.09 | 5088.12 |
| 2 | 6171.10 | 8193.09 | 5378.68 | 5826.25 | 4404.11 | 7436.98 | 5177.66 |
| 3 | 6171.47 | 8209.93 | 5427.22 | 5827.70 | 4450.90 | 7453.21 | 5222.62 |

Note: The bold figures represent the AIC values of the selected models.

#

# Table S8 Cox-PH model analysis: Hazard ratios for PFS in first-line therapies

| Treatment | Hazard ratio (95% CI) | P-score | Rank | Treatment | Hazard ratio (95% CI) | P-score | Rank |
| --- | --- | --- | --- | --- | --- | --- | --- |
| Abema/LET | 0.50 (0.42 ~ 0.60) | 0.9288 | 1 | LET | - | 0.4037 | 9 |
| Dalpiciclib/LET | 0.52 (0.36 ~ 0.75) | 0.8796 | 2 | FUL | 1.21 (0.82 ~ 1.79) | 0.3248 | 10 |
| Ribo/LET | 0.57 (0.46 ~ 0.70) | 0.8219 | 3 | FUL/ANA | 1.23 (0.76 ~ 2.01) | 0.3241 | 11 |
| Palbo/LET | 0.59 (0.51 ~ 0.68) | 0.7921 | 4 | EXE | 1.53 (0.91 ~ 2.59) | 0.1674 | 12 |
| Palbo/FUL | 0.67 (0.50 ~ 0.89) | 0.6827 | 5 | ANA | 1.52 (0.97 ~ 2.39) | 0.1638 | 13 |
| Abema/FUL | 0.70 (0.44 ~ 1.10) | 0.6623 | 6 | Sapitinib40mg/ANA | 1.77 (0.96 ~ 3.25) | 0.0999 | 14 |
| Ribo/FUL | 0.67 (0.50 ~ 0.89) | 0.6605 | 7 | Sapitinib20mg/ANA | 2.09 (1.13 ~ 3.83) | 0.0353 | 15 |
| BEV/LET | 0.79 (0.66 ~ 0.94) | 0.5531 | 8 |  |  |  |  |

Abbreviations: Abema, Abemaciclib; ANA, Anastrozole; BEV, Bevacizumab; EXE, Exemestane; FUL, Fulvestrant; LET: Letrozole; Palbo, Palbociclib; Ribo, Ribociclib.

# Table S9 Hazard ratios for PFS and OS in second/further-lines therapies: Cox-PH model analysis

A: PFS

| Treatment | Hazard ratio (95% CI) | P-score | Rank | Treatment | Hazard ratio (95% CI) | P-score | Rank |
| --- | --- | --- | --- | --- | --- | --- | --- |
| Eribulin or Gemcitabine or Capecitabine | 0.31 (0.21 ~ 0.46) | 0.9790 | 1 | Tucidinostat/EXE | 0.74 (0.53 ~ 1.03) | 0.4119 | 15 |
| EVE/EXE | 0.39 (0.30 ~ 0.52) | 0.9177 | 2 | Pictilisib/FUL | 0.74 (0.52 ~ 1.06) | 0.4069 | 16 |
| Dalpiciclib/FUL | 0.44 (0.31 ~ 0.62) | 0.8591 | 3 | Taselisib/FUL | 0.74 (0.59 ~ 0.93) | 0.4047 | 17 |
| Abema/FUL | 0.48 (0.38 ~ 0.61) | 0.8204 | 4 | Palbo/FUL/Avelumab | 0.75 (0.47 ~ 1.20) | 0.3965 | 18 |
| Ribo/EXE | 0.43 (0.14 ~ 1.28) | 0.7564 | 5 | Buparlisib/FUL | 0.75 (0.66 ~ 0.84) | 0.3905 | 19 |
| Palbo/FUL | 0.52 (0.41 ~ 0.66) | 0.7515 | 6 | FUL/Sapanisertib4mg/d | 0.77 (0.47 ~ 1.26) | 0.3726 | 20 |
| EVE | 0.53 (0.36 ~ 0.78) | 0.7186 | 7 | ALP/FUL | 0.85 (0.58 ~ 1.25) | 0.2710 | 21 |
| CAP/FUL | 0.56 (0.38 ~ 0.82) | 0.6805 | 8 | FUL/Sapanisertib30mg/w | 0.88 (0.53 ~ 1.46) | 0.2545 | 22 |
| Ribo/FUL | 0.57 (0.45 ~ 0.73) | 0.6700 | 9 | ENT/EXE | 0.89 (0.71 ~ 1.11) | 0.2200 | 23 |
| Camizestrant75mg | 0.58 (0.41 ~ 0.82) | 0.6527 | 10 | FUL/daily Vistusertib | 0.90 (0.68 ~ 1.20) | 0.2034 | 24 |
| FUL/EVE | 0.59 (0.43 ~ 0.80) | 0.6465 | 11 | Lapatinib/FUL | 1.00 (0.76 ~ 1.31) | 0.1176 | 25 |
| Camizestrant150mg | 0.67 (0.48 ~ 0.93) | 0.5106 | 12 | FUL | - | 0.0991 | 26 |
| Elacestrant | 0.68 (0.52 ~ 0.89) | 0.4965 | 13 | EXE | 1.04 (0.88 ~ 1.22) | 0.0702 | 27 |
| FUL/Bortezomib | 0.73 (0.49 ~ 1.09) | 0.4215 | 14 |  |  |  |  |

Abbreviations: Abema, Abemaciclib; ALP, Alpelisib; CAP, Capivasertib; ENT, Entinostat; EXE, Exemestane; EVE, Everolimus; FUL, Fulvestrant; Palbo, Palbociclib; Ribo, Ribociclib.

B: OS

| Treatment | Hazard ratio (95% CI) | P-score | Rank | Treatment | Hazard ratio (95% CI) | P-score | Rank |
| --- | --- | --- | --- | --- | --- | --- | --- |
| Abema/FUL | 0.66 (0.46 ~ 0.93) | 0.8372 | 1 | ALP/FUL | 0.86 (0.64 ~ 1.15) | 0.5199 | 9 |
| CAP/FUL | 0.66 (0.45 ~ 0.97) | 0.8244 | 2 | Buparlisib/FUL | 0.87 (0.74 ~ 1.02) | 0.5043 | 10 |
| Palbo/FUL/Avelumab | 0.68 (0.40 ~ 1.15) | 0.7633 | 3 | Lapatinib/FUL | 0.91 (0.68 ~ 1.21) | 0.4380 | 11 |
| Elacestrant | 0.75 (0.54 ~ 1.04) | 0.7017 | 4 | FUL | - | 0.2683 | 12 |
| Ribo/FUL | 0.78 (0.59 ~ 1.04) | 0.6587 | 5 | EVE/EXE | 1.08 (0.67 ~ 1.77) | 0.2612 | 13 |
| Eribulin or Gemcitabine or Capecitabine | 0.82 (0.57 ~ 1.17) | 0.6159 | 6 | ENT/EXE | 1.13 (0.64 ~ 1.97) | 0.2396 | 14 |
| EVE | 0.85 (0.48 ~ 1.53) | 0.5575 | 7 | FUL/EVE | 1.31 (0.72 ~ 2.38) | 0.1471 | 15 |
| Palbo/FUL | 0.86 (0.70 ~ 1.05) | 0.5339 | 8 | EXE | 1.22 (0.72 ~ 2.07) | 0.1291 | 16 |

Abbreviations: Abema, Abemaciclib; ALP, Alpelisib;CAP, Capivasertib; ENT, Entinostat; EVE, Everolimus; EXE, Exemestane; FUL, Fulvestrant; Palbo, Palbociclib; Ribo, Ribociclib

# Table S10 Comparison of adverse events for first-line therapies

A: any-grade adverse events for first-line therapies

| Treatment | Log odds ratio (95% CI) | Rank | Treatment | Log odds ratio (95% CI) | Rank |
| --- | --- | --- | --- | --- | --- |
| ANA | -21.48 (-32.84 ~ -2.77) | 1 | LET | - | 7 |
| FUL | -21.32 (-32.62 ~ -2.63) | 2 | FUL/ANA | 8.30 (-53.54 ~ 88.04) | 8 |
| EXE | -21.14 (-32.48 ~ -2.36) | 3 | Ribo/LET | 0.92 (-0.39 ~ 2.54) | 9 |
| Sapitinib20mg/ANA | -20.20 (-31.56 ~ -1.34) | 4 | Palbo/LET | 2.03 (1.26 ~ 2.96) | 10 |
| Sapitinib40mg/ANA | -19.70 (-31.04 ~ -0.82) | 5 | Abema/LET | 2.23 (1.30 ~ 3.37) | 11 |
| Abema/FUL | -18.67 (-29.91 ~ 0.44) | 6 | Palbo/FUL | 2.90 (0.21 ~ 6.51) | 12 |

Abbreviations: Abema, Abemaciclib; ANA, Anastrozole; EXE, Exemestane; FUL, Fulvestrant; LET, Letrozole; Palbo, Palbociclib; Ribo, Ribociclib.

B: 3-5 Grade adverse events for first-line therapies

| Treatment | Log odds ratio (95% CI) | Rank | Treatment | Log odds ratio (95% CI) | Rank |
| --- | --- | --- | --- | --- | --- |
| LET | - | 1 | FUL/ANA | 8.47 (-53.45 ~ 52.83) | 8 |
| FUL | 1.44 (-1.54 ~ 4.30) | 2 | EXE | 8.52 (-53.49 ~ 52.86) | 9 |
| Abema/LET | 1.44 (0.12 ~ 2.81) | 3 | Palbo/LET | 2.67 (1.56 ~ 3.78) | 10 |
| BEV/LET | 1.71 (-0.27 ~ 3.67) | 4 | Palbo/FUL | 2.82 (0.60 ~ 4.99) | 11 |
| Sapitinib20mg/ANA | 8.09 (-53.98 ~ 52.30) | 5 | Abema/FUL | 3.20 (-0.39 ~ 6.61) | 12 |
| Ribo/LET | 2.40 (0.49 ~ 4.32) | 6 | Sapitinib40mg/ANA | 9.01 (-53.05 ~ 53.31) | 13 |
| ANA | 8.34 (-53.60 ~ 52.64) | 7 |  |  |  |

Abbreviations: Abema, Abemaciclib; ANA, Anastrozole; BEV, Bevacizumab; EXE, Exemestane; FUL, Fulvestrant; LET, Letrozole; Palbo, Palbociclib; Ribo, Ribociclib.

C: Adverse events leading to discontinuation for first-line therapies

| Therapy | Log odds ratio (95% CI) | Rank |
| --- | --- | --- |
| LET | - | 1 |
| Palbo/LET | 1.0 (0.5 ~ 1.5) | 2 |
| ANA | 1.2 (-1.1 ~ 3.3) | 3 |
| EXE | 1.3 (-1.2 ~ 3.6) | 4 |
| Ribo/LET | 1.3 (0.5 ~ 2.1) | 5 |
| FUL | 1.6 (-0.5 ~ 3.6) | 6 |
| Sapitinib20mg/ANA | 2.0 (-0.7 ~ 4.5) | 7 |
| Abema/LET | 2.0 (1.2 ~ 2.9) | 8 |
| Palbo/FUL | 2.0 (0.9 ~ 3.2) | 9 |
| Sapitinib40mg/ANA | 2.5 (-0.1 ~ 5.0) | 10 |
| Ribo/FUL | 3.3 (1.1 ~ 5.5) | 11 |

Abbreviations: Abema, Abemaciclib; ANA, Anastrozole; EXE, Exemestane; FUL, Fulvestrant; LET, Letrozole; Palbo, Palbociclib; Ribo, Ribociclib.

D: Adverse events leading to death for first-line therapies

| Therapy | Log odds ratio (95% CI) | Rank |
| --- | --- | --- |
| FUL | -0.54 (-2.19 ~ 0.92) | 1 |
| LET or ANA | - | 2 |
| FUL/ANA | 0.11 (-0.34 ~ 0.56) | 3 |
| Palbo/LET | 0.27 (-0.86 ~ 1.64) | 4 |
| Palbo/FUL | 0.36 (-2.70 ~ 3.99) | 5 |
| Ribo/LET | 0.93 (-0.38 ~ 2.46) | 6 |
| Abema/LET | 1.14 (-0.25 ~ 3.16) | 7 |
| EXE | 1.33 (-0.90 ~ 4.57) | 8 |
| BEV/LET | 1.68 (1.17 ~ 2.25) | 9 |

Abbreviations: Abema, Abemaciclib; ANA, Anastrozole; BEV, Bevacizumab; EXE, Exemestane; FUL, Fulvestrant; LET, Letrozole; Palbo, Palbociclib; Ribo, Ribociclib.

# Table S11 Comparison of adverse events for second/further-lines therapies

A: any-grade adverse events for second/further-lines therapies

| Treatment | Log odds ratio (95% CI) | Rank | Treatment | Log odds ratio (95% CI) | Rank |
| --- | --- | --- | --- | --- | --- |
| EVE | -22.0 (-53.7 ~ 0.7) | 1 | ENT/EXE | 2.2 (1.2 ~ 3.4) | 10 |
| FUL | 0.3 (-0.4 ~ 1.0) | 2 | Tucidinostat/EXE | 2.4 (1.5 ~ 3.6) | 11 |
| Elacestrant | 0.4 (-0.3 ~ 1.2) | 3 | Abema/FUL | 2.5 (1.2 ~ 4.5) | 12 |
| EXE | 0.9 (0.2 ~ 1.5) | 4 | Buparlisib/FUL | 19.0 (-30.3 ~ 93.1) | 13 |
| Camizestrant75mg | 1.2 (0.4 ~ 2.0) | 5 | ALP/FUL | 2.9 (1.8 ~ 4.0) | 14 |
| Taselisib/FUL | 1.5 (0.6 ~ 2.5) | 6 | EVE/EXE | 30.7 (3.3 ~ 97.9) | 15 |
| Pictilisib/FUL | 1.7 (0.9 ~ 2.6) | 7 | Eribulin or Gemcitabine or Capecitabine | 31.0 (3.3 ~ 98.8) | 16 |
| Camizestrant150mg | 1.8 (0.5 ~ 3.4) | 8 | FUL/Sapanisertib30mg/w | 2.2 (1.2 ~ 3.4) | 17 |
| Palbo/FUL | 2.2 (1.1 ~ 3.5) | 9 | FUL/Sapanisertib4mg/d | 2.4 (1.5 ~ 3.6) | 18 |

Abbreviations: Abema, Abemaciclib; ALP, Alpelisib; CAP, Capivasertib; ENT, Entinostat; EVE, Everolimus; EXE, Exemestane; FUL, Fulvestrant; Palbo, Palbociclib; Ribo, Ribociclib

B: 3-5 Grade adverse events for second/further-lines therapies

| Treatment | Log odds ratio (95% CI) | Rank | Treatment | Log odds ratio (95% CI) | Rank |
| --- | --- | --- | --- | --- | --- |
| FUL | - | 1 | EVE/EXE | 1.5 (-2.1 ~ 4.8) | 12 |
| ALP/FUL | 0.2 (-4.0 ~ 1.0) | 2 | Camizestrant150mg | 1.5 (-0.6 ~ 3.6) | 13 |
| EXE | 0.4 (-3.8 ~ 4.1) | 3 | Taselisib/FUL | 1.6 (-0.4 ~ 3.6) | 14 |
| Camizestrant75mg | 0.5 (-1.6 ~ 2.5) | 4 | Eribulin or Gemcitabine or Capecitabine | 1.7 (-1.2 ~ 4.3) | 15 |
| FUL/Bortezomib | 0.8 (-1.3 ~ 2.9) | 5 | Abema/FUL | 1.8 (0.4 ~ 3.2) | 16 |
| CAP/FUL | 0.9 (-1.2 ~ 3.0) | 6 | ENT/EXE | 1.9 (-2.6 ~ 5.7) | 17 |
| Elacestrant | 0.9 (-1.2 ~ 3.1) | 7 | FUL/Sapanisertib4mg/d | 1.9 (-0.2 ~ 4.1) | 18 |
| EVE | 1.0 (-2.6 ~ 4.3) | 8 | Tucidinostat/EXE | 2.2 (-2.5 ~ 6.4) | 19 |
| Pictilisib/FUL | 1.4 (-0.6 ~ 3.4) | 9 | FUL/EVE | 2.2 (0.1 ~ 4.3) | 20 |
| Lapatinib/FUL | 1.5 (-0.6 ~ 3.6) | 10 | Palbo/FUL | 2.3 (0.3 ~ 4.2) | 21 |
| FUL/Sapanisertib30mg/w | 1.5 (-0.5 ~ 3.7) | 11 | Buparlisib/FUL | 2.3 (1.0 ~ 3.8) | 22 |

Abbreviations: Abema, Abemaciclib; ALP, Alpelisib; CAP, Capivasertib; ENT, Entinostat; EVE, Everolimus; EXE, Exemestane; FUL, Fulvestrant; Palbo, Palbociclib; Ribo, Ribociclib

C: Adverse events leading to discontinuation for second/further-lines therapies

| Treatment | Log odds ratio (95% CI) | Rank | Treatment | Log odds ratio (95% CI) | Rank |
| --- | --- | --- | --- | --- | --- |
| Palbo/FUL | -0.5 (-1.7 ~ 0.6) | 1 | FUL/EVE | 1.3 (0.4 ~ 2.4) | 12 |
| ANA | -0.1 (-0.8 ~ 0.5) | 2 | Camizestrant75mg | 1.5 (0.2 ~ 3.1) | 13 |
| FUL | - | 3 | FUL/daily Vistusertib | 1.4 (0.1 ~ 2.9) | 14 |
| EVE/EXE | 0.1 (-0.4 ~ 0.6) | 4 | Pictilisib/FUL | 1.8 (0.7 ~ 3.1) | 15 |
| EXE | 0.1 (-0.3 ~ 0.5) | 5 | Elacestrant | 2.1 (0.2 ~ 5.0) | 16 |
| EVE | 0.3 (-0.6 ~ 1.1) | 6 | Camizestrant150mg | 2.0 (0.8 ~ 3.6) | 17 |
| Eribulin or Gemcitabine or Capecitabine | 0.3 (-0.5 ~ 1.2) | 7 | Abema/FUL | 2.0 (0.7 ~ 4.0) | 18 |
| Buparlisib/FUL | 0.4 (0.1 ~ 0.6) | 8 | ALP/FUL | 2.1 (1.4 ~ 2.7) | 19 |
| FUL/intermittent Vistusertib | 0.7 (-0.9 ~ 2.3) | 9 | Taselisib/FUL | 2.2 (1.4 ~ 3.3) | 20 |
| Tucidinostat/EXE | 1.0 (-0.0 ~ 2.2) | 10 | FUL/Sapanisertib4mg/d | 2.5 (1.1 ~ 4.5) | 21 |
| ENT/EXE | 1.0 (0.4 ~ 1.7) | 11 | FUL/Sapanisertib30mg/w | 2.7 (1.3 ~ 4.7) | 22 |

Abbreviations: Abema, Abemaciclib; ALP, Alpelisib; ANA, Anastrozole; CAP, Capivasertib; ENT, Entinostat; EVE, Everolimus; EXE, Exemestane; FUL, Fulvestrant; Palbo, Palbociclib; Ribo, Ribociclib

D: Adverse events leading to death for second/further-lines therapies

| Therapy | Log odds ratio (95% CI) | Rank |
| --- | --- | --- |
| EXE | -1.0 (-3.4 ~ 1.0) | 1 |
| Palbo/FUL | -0.1 (-5.2 ~ 4.5) | 2 |
| FUL | - | 3 |
| EVE/EXE | 0.7 (-2.8 ~ 4.4) | 4 |
| CAP/FUL | 0.9 (-1.9 ~ 4.4) | 5 |
| Eribulin or Gemcitabine or Capecitabine | 1.0 (-3.1 ~ 5.0) | 6 |
| EVE | 1.2 (-2.8 ~ 5.2) | 7 |
| FUL/EVE | 1.4 (-1.1 ~ 5.1) | 8 |

Abbreviations: CAP, Capivasertib; EVE, Everolimus; EXE, Exemestane; FUL, Fulvestrant; Palbo, Palbociclib.

# Table S12 Summary of hematologic and non-hematologic adverse events in RCTs

| Trial information | Intervention arm | Control arm | Any hematologic toxicities | Grade 3-5 hematologic toxicities | Any nonhematologic toxicities | Grade 3-5 nonhematologic toxicities |
| --- | --- | --- | --- | --- | --- | --- |
| PALOMA-3[33-36] | Palbociclib/Fulvestrant (CDK4/6i/SERD) | Fulvestrant (SERD) | Neutropenia 79.6% vs2.9%  Leukopenia 47.8% vs4.4%  Anemia 29.6% vs12.5% | Neutropenia 62.0% vs0.6%  Leukopenia 25.2% vs0.6%  Anemia 2.6% vs1.7% | Infections 40.1% vs29.4%  Nausea 30.3% vs25.0%  Fatigue 40.1% vs27.9% | Infections 5.0% vs3.0%  Back pain 1.5% vs0.7%  Fatigue 2.9% vs1.5% |
| PALOMA-2[37-39] | Palbociclib/Letrozole (CDK4/6i/AI) | Letrozole (AI) | Neutropenia 79.5% vs6.3%  Leukopenia 39.0% vs2.3%  Anemia 24.1% vs9.0% | Neutropenia 66.4% vs1.4%  Leukopenia 24.8% vs0.6%  Anemia 5.4% vs1.8% | Fatigue 37.4% vs27.5%  Nausea 35.1% vs26.1%  Arthralgia 33.3% vs33.8% | Fatigue 1.8% vs0.5%  Arthralgia 0.7% vs0.5%  Nausea 0.2% vs1.8% |
| PALOMA-4[40] | Palbociclib/Letrozole (CDK4/6i/AI) | Letrozole (AI) | Neutropenia 98.2% vs17.0%  Leukopenia 85.7% vs12.9%  Thrombocytopaenia 48.8% vs4.1% | Neutropenia 84.5% vs1.2%  Leukopenia 36.3% vs0.6%  Thrombocytopaenia 6.6% vs0.6% | Aspartate aminotransferase increased 34.5% vs28.1%  Alanine aminotransferase increased 33.3% vs32.7%  Infections 31.0% vs31.0% | Aspartate aminotransferase increased 3.6% vs1.8%  Alanine aminotransferase increased 4.8% vs0.6%  Infections 3.0% vs4.1% |
| PALOMA-1/TRIO-18[41.42] | Palbociclib/Letrozole (CDK4/6i/AI) | Letrozole (AI) | Neutropenia 75% vs 5%  Leukopenia 43% vs 4%  Anemia 35% vs 5% | Neutropenia 59% vs 1%  Leukopenia 18% vs 0%  Anemia 6% vs 1% | Fatigue 41% vs 23%  Nausea 30% vs 14%  Arthralgia 27% vs 18% | Fatigue 7% vs 1%  Nausea 2% vs 1%  Arthralgia 2% vs 3% |
| MONALEESA-2[43-45] | Ribociclib/Letrozole (CDK4/6i/AI) | Letrozole (AI) | Neutropenia 76.9% vs 5.8%  Leukopenia 32.9% vs 4.5%  Anemia 21.3% vs 5.8% | Neutropenia 62.0% vs 1.2%  Leukopenia 21.3% vs 0.9%  Anemia 2.4% vs 1.2% | Nausea 53.3% vs 30.6%  Fatigue 41.3% vs 32.4%  Diarrhea 38.3% vs 24.5% | Abnormal LFTs 10.2% vs 2.4%  Vomiting 3.6% vs 0.9%  Fatigue 3.0% vs 0.9% |
| MONALEESA-3[46,47] | Ribociclib/Fulvestrant (CDK4/6i/SERD) | Fulvestrant (SERD) | Neutropenia 71.6% vs 2.9%  Leukopenia 30.6% vs 1.7%  Anemia 19.0% vs 7.1% | Neutropenia 53.4% vs 0%  Leukopenia 14.1% vs 0%  Anemia 3.1% vs 2.1% | Fatigue 31.5% vs 33.2%  Diarrhea 29.0% vs 20.3%  Vomiting 26.7% vs 12.9% | Fatigue 1.7% vs 0.4%  Vomiting 1.4% vs 0%  Diarrhea 0.6% vs 0.8% |
| MONARCH-2(2nd)[48,49] | Abemaciclib/Fulvestrant (CDK4/6i/SERD) | Fulvestrant (SERD) | Neutropenia 46.0% vs 4.0%  Leukopenia 28.3% vs 1.8%  Anemia 29.0% vs 3.6% | Neutropenia 26.5% vs 1.7%  Leukopenia 8.8% vs 0%  Anemia 7.2% vs 0.9% | Diarrhea 86.4% vs 24.7%  Nausea 45.1% vs 22.9%  Fatigue 39.9% vs 26.9% | Diarrhea 13.4% vs 0.4%  Nausea 2.7% vs 0.9%  Fatigue 2.7% vs 0.4% |
| MONARCH-3[50-52] | Abemaciclib/Letrozole (CDK4/6i/AI) | Letrozole (AI) | Neutropenia 43.7% vs 1.9%  Anemia 31.5% vs 8.1%  Leukopenia 22.0% vs 2.5% | Neutropenia 23.8% vs 1.2%  Anemia 22.0% vs 3.1%  Leukopenia 8.6% vs 0.6% | Diarrhea 82.3% vs 32.3%  Fatigue 41.3% vs 33.5%  Nausea 41.3% vs 20.5% | Diarrhea 9.5% vs 1.2%  ALT increased 6.4% vs 1.9%  AST increased 3.7% vs 1.2% |
| PARSIFAL[53] | Palbociclib/Fulvestrant (CDK4/6i/SERD) | Palbociclib/Letrozole (CDK4/6i/AI) | Neutropenia 82.2% vs 85.5%  Leukopenia 24.9% vs 25.2%  Anemia 22.8% vs 28.1% | Neutropenia 66.0% vs 68.2%  Leukopenia 7.0% vs 5.8%  Anemia 2.5% vs 2.5% | Asthenia 37.3% vs 36.0%  Diarrhea 27.0% vs 24.8%  Arthralgia 25.7% vs 33.1% | Asthenia 2.9% vs 2.1%  Back pain 2.9% vs 0.4%  Fatigue 1.7% vs 1.7% |
| FLIPPER[54] | Palbociclib/Fulvestrant (CDK4/6i/SERD) | Fulvestrant (SERD) | Neutropenia 96.8% vs 23.2%  white blood cell decreased 93.6% vs 25.3%  Anemia 85.1% vs 34.7% | Neutropenia 68.1% vs 0%  white blood cell decreased 26.6% vs 0%  Lymphocytopenia 14.9% vs 2.1% | Fatigue 64.9% vs 44.2%  Hypertension 45.7% vs 43.2%  Arthralgia 33.0% vs 28.4% | Hypertension 19.1% vs 13.7%  Respiratory infection 10.6% vs 0%  Weight gain 7.4% vs 4.2% |
| MONARCH plus A[55,56] | Abemaciclib/Letrozole (CDK4/6i/AI) | Letrozole (AI) | Neutropenia 80.0% vs 20.2%  Leukopenia 76.1% vs 27.3%  Anemia 62.0% vs 20.2% | Neutropenia 26.4% vs 6.1%  Leukopenia 13.2% vs 2.0%  Anemia 11.2% vs 3.0% | Diarrhea 80.0% vs 1.0%  ALT increased 34.6% vs 23.2%  AST increased 34.6% vs 21.2% | ALT increased 5.9% vs 1.0%  AST increased 4.4% vs 2.0%  Diarrhea 3.9% vs 16.2% |
| MONARCH plus B[55,56] | Abemaciclib/Fulvestrant (CDK4/6i/SERD) | Fulvestrant (SERD) | Leukopenia 82.7% vs 22.6%  Neutropenia 80.8% vs 18.9%  Anemia 70.2% vs 15.1% | Neutropenia 29.8% vs 3.8%  Leukopenia 22.1% vs 3.8%  Anemia 10.6% vs 1.9% | Diarrhea 78.8% vs 9.4%  ALT increased 34.6% vs 22.6%  AST increased 30.8% vs 26.4% | ALT increased 5.8% vs 0.0%  AST increased 2.9% vs 0.0%  Pain 1.9% vs 1.9% |
| DAWNA-1[57,58] | Dalpiciclib/Fulvestrant (CDK4/6i/SERD) | Fulvestrant (SERD) | Neutropenia 97.9% vs 12.5%  Leukopenia 97.1% vs 27.3%  Anemia 61.3% vs 11.7% | Neutropenia 84.2% vs 0.0%  Leukopenia 62.1% vs 0.0%  Thrombocytopenia 5.8% vs 0.8% | AST increased 20.0% vs 25.8%  Nausea 18.8% vs 14.2%  ALT increased 15.0% vs 26.7% | AST increased 0.4% vs 1.6%  Nausea 0.4% vs 0.8%  ALT increased 0.4% vs 1.6% |
| DAWNA-2[59] | Dalpiciclib/Letrozole (CDK4/6i/AI) | Letrozole (AI) | NA | NA | NA | NA |
| BOLERO-2[60,61] | Everolimus/Exemestane (mTORi/AI) | Exemestane (AI) | Anemia 21% vs 5% | Anemia 8% vs 2% | Stomatitis 59% vs 12%  Rash 39% vs 7%  Fatigue 37% vs 27% | Stomatitis 8% vs 1%  Fatigue 5% vs 1%  Dyspnea 6% vs 2% |
| MANTA(1)[62] | Fulvestrant/Everolimus (SERD/mTORi) | Fulvestrant (SERD) | NA | NA | Asthenia 53.3% vs 16.1%  Nausea 26.7% vs 12.5%  Headache 18.3% vs 12.5% | Stomatitis 11.7% vs 0%  Infection 6.7% vs 0.0%  Rash 5.0% vs 0.0% |
| MANTA(2)[62] | Fulvestrant/daily Vistusertib (SERD/mTORi) | Fulvestrant (SERD) | NA | NA | Rash 54.3% vs 0.0%  Stomatitis 40.2% vs 0.0%  Asthenia 34.8% vs 16.1% | Rash 20.7% vs 0.0%  Stomatitis 13.0% vs 0.0%  Infection 5.4% vs 0.0% |
| MANTA(3)[62] | Fulvestrant/intermittent Vistusertib (SERD/mTORi) | Fulvestrant (SERD) | NA | NA | Nausea 68.5% vs 12.5%  Asthenia 45.7% vs 16.1%  Vomiting 40.2% vs 0.0% | Asthenia 5.4% vs 0.0%  Diarrhoea 5.4% vs 0.0%  Vomiting 5.4% vs 0.0% |
| PrE0102  [63] | Fulvestrant/Everolimus (SERD/mTORi) | Fulvestrant (SERD) | NA | NA | Oral mucositis 53% vs 12%  Fatigue 42% vs 22%  Rash 38% vs 5% | Oral mucositis 11% vs 0%  Fatigue 6% vs 5%  Rash 2% vs 0% |
| BOLERO-6(1)[64] | Everolimus/Exemestane (mTORi/AI) | Everolimus (mTORi) | Anemia 32% vs 25%  Neutropenia 4% vs 4% | Anemia 13% vs 10%  Neutropenia 0% vs 2% | Stomatitis 49% vs 46%  Fatigue 38% vs 31%  Diarrhea 35% vs33% | Stomatitis 9% vs 5%  AST increased 7% vs 8%  Pneumonia 7% vs 3% |
| BOLERO-6(2)[64] | Everolimus/Exemestane (mTORi/AI) | Capecitabine (single-agent chemotherapy) | Anemia 32% vs 22%  Neutropenia 4% vs 15% | Anemia 13% vs 7%  Neutropenia 0% vs 6% | Stomatitis 49% vs 25%  Fatigue 38% vs 35%  Diarrhea 35% vs54% | Stomatitis 9% vs 7%  Fatigue 8% vs 8%  Diarrhea 5% vs 8% |
| SWOG S0226  [65,66] | Anastrozole (AI) | Fulvestrant/Anastrozole (SERD/AI) | NA | NA | NA | NA |
| FALCON[67] | Fulvestrant (SERD) | Anastrozole (AI) | Anaemia 4% vs 9% | NA | Arthralgia 17% vs 10%  Hot flush 11% vs 10%  Nausea 11% vs 10% | NA |
| BELLE-3[68] | Buparlisib/Fulvestrant (PI3Ki/SERD) | Fulvestrant (SERD) | NA | NA | Increased ALT 39% vs 7%  Increased AST 37% vs 10%  Hyperglycaemia 37% vs 3% | Increased ALT 22% vs 3%  Increased AST 18% vs 3%  Hyperglycaemia 13% vs 0% |
| SOLAR-1  [69,70] | Alpelisib/Fulvestrant (PI3Ki/SERD) | Fulvestrant (SERD) | NA | NA | Hyperglycemia 63.7% vs 9.8%  Diarrhea 57.7% vs 15.7%  Nausea 44.7% vs22.3% | Hyperglycemia 36.6% vs 0.6%  Diarrhea 6.7% vs 0.3%  Nausea 2.5% vs0.3% |
| FAKTION[71] | Capivasertib/Fulvestrant (AKTi/SERD) | Fulvestrant (SERD) | Anaemia 4% vs3%  Neutrophil count decreased 11% vs 12% | Anaemia 0% vs3% | Diarrhoea 81% vs 35%  Rash 52% vs 18%  Hyperglycaemia 42% vs 14% | Hypertension 32% vs 24%  Rash 20% vs 0%  Diarrhoea 14% vs 4% |
| ACE[72] | Tucidinostat/Exemestane (HDACi/AI) | Exemestane (AI) | Neutropenia 82% vs 26%  Leucopenia 80% vs 25%  Thrombocytopenia 75% vs 14% | Neutropenia 9% vs 2%  Leucopenia 1% vs 2%  Thrombocytopenia 27% vs 3% | Hyperglycaemia 25% vs 14%  Increased aspartate  Aminotransferase 20% vs 20%  Nausea 26% vs 6% | Hyperglycaemia 5% vs 1%  Diarrhoea 2% vs 0%  Nausea 1% vs 0% |
| BELLE-2[73,74] | Buparlisib/Fulvestrant (PI3Ki/SERD) | Fulvestrant (SERD) | NA | NA | Hyperglycemia 43% vs 8%  Elevated ALT 40% vs 7%  Nausea 40% vs 24% | Elevated ALT 26% vs 1%  Elevated AST 18% vs 3%  Hyperglycemia 15% vs 1% |
| FERGI  [75] | Pictilisib/Fulvestrant (PI3Ki/SERD) | Fulvestrant (SERD) | NA | Anaemia 1% vs 4%  Neutropenia 3% vs 0% | NA | Rash 9% vs 0%  Diarrhoea 8% vs 0%  Fatigue 8% vs 0% |
| LEA[76] | Bevacizumab/Letrozole (Anti-VEGF/AI) | Letrozole (AI) | Anemia 100% vs 98%  Leukopenia 26% vs 12%  Neutropenia 12% vs 12% | Anemia 1% vs 1%  Leukopenia 2% vs 0%  Neutropenia 1% vs 0% | Hypertension 76% vs 20%  Pain 76% vs 51%  Elevated liver enzyme 60.5% vs 39.6% | Hypertension 15% vs 3%  Pain 5% vs 2%  Elevated liver enzyme 3.7% vs 1% |
| NCT02756364(1)  [77] | Fulvestrant/Sapanisertib4mg/d (SERD/mTORi) | Fulvestrant (SERD) | NA | NA | Hyperglycemia 61.7% vs 0%  Nausea 48.9% vs 4.3%  Diarrhea 48.9% vs 0% | Rash 14.9% vs 0%  Diarrhea 10.6% vs 0%  Stomatitis 8.5% vs 0% |
| NCT02756364(2)  [77] | Fulvestrant/Sapanisertib30mg/w (SERD/mTORi) | Fulvestrant (SERD) | NA | NA | Nausea 91.5% vs 4.3%  Vomiting 80.9% vs 4.3%  Stomatitis 36.2% vs 2.2% | Vomiting 14.9% vs 0%  Fatigue 10.6% vs 0%  Nausea 8.5% vs 0% |
| SANDPIPER[78] | Taselisib/Fulvestrant (PI3Ki/SERD) | Fulvestrant (SERD) | NA | Neutropenia 1.7% vs 0.9% | Diarrhea 60.1% vs 19.7%  Hyperglycemia 40.4% vs 9.4%  Nausea 34.1% vs 24.4% | Diarrhea 11.5% vs 0.9%  Hyperglycemia 10.8% vs 0.5%  Stomatitis 3.6% vs 0% |
| EMERALD[79] | Elacestrant (SERD) | Fulvestrant (SERD) | NA | NA | Nausea 35.0% vs 16.1%  Fatigue 19.0% vs 21.7%  Vomiting 19.0% vs 7.5% | Nausea 2.5% vs 0%  Back pain 2.5% vs 0.6%  AST increased 1.7% vs 1.2% |
| E2112  [80] | Entinostat/Exemestane (HDACi/AI) | Exemestane (AI) | Platelet count decreased 63% vs 8%  White blood cell decreased 55% vs 14%  Neutrophil count decreased 51% vs 5% | Neutrophil count decreased 20% vs 1%  Anemia 8% vs 2%  White blood cell decreased 6% vs 1% | Fatigue 57% vs 38%  Nausea 41% vs 27%  Diarrhea 36% vs 17% | Fatigue 4% vs 1%  Diarrhea 4% vs 1%  Nausea 2% vs 1% |
| ENCORE301  [81] | Entinostat/Exemestane (HDACi/AI) | Exemestane (AI) | Neutropenia 30% vs 0%  Anemia 19% vs 12%  Thrombocytopenia 19% vs 6% | Neutropenia 15% vs 0%  Anemia 2% vs 4%  Thrombocytopenia 2% vs 2% | Fatigue 48% vs 26%  Nausea 40% vs 15%  Peripheral edema 21% vs 5% | Fatigue 13% vs 3%  Nausea 5% vs 2%  Vomiting 5% vs 0% |
| MINT(1)  [82] | Sapitinib40mg/Anastrozole (EGFRi/AI) | Anastrozole (AI) | NA | NA | Diarrhea 51% vs 12%  Rash 48% vs 12%  Dry skin 25% vs 2% | NA |
| MINT(2)  [82] | Sapitinib20mg/Anastrozole (EGFRi/AI) | Anastrozole (AI) | NA | NA | Diarrhea 40% vs 12%  Rash 32% vs 12%  Dry skin 19% vs 2% | NA |
| Hiroji Iwata2013[83] | Exemestane (AI) | Anastrozole (AI) | NA | NA | Hot flush 22% vs15%  Arthralgia 17% vs17%  Musculoskeletal stiffness 11% vs 7% | NA |
| Alliance CALGB40503[84] | Bevacizumab/Letrozole (Anti-VEGF/AI) | Letrozole (AI) | NA | NA | NA | Hypertension 24% vs 2%  Proteinuria 11% vs0%  Joint pain 10% vs 0% |
| EFECT  [85] | Fulvestrant (SERD) | Exemestane (AI) | NA | NA | Hot flashes 8.8% vs11.5%  Nausea 6.8% vs 7.9%  Fatigue 6.3% vs 10.0% | NA |
| Alliance CALGB 40302  [86] | Lapatinib/Fulvestrant (EGFRi/SERD) | Fulvestrant (SERD) | NA | NA | NA | Diarrhea 8% vs 0%  Fatigue 3% vs 0%  Aspartate aminotransferase increased 3% vs 1% |
| NCT01142401[87] | Fulvestrant/Bortezomib (SERD/Pi) | Fulvestrant (AI) | Anemia 43% vs 33%  Neutropenia 30% vs 8%  Thromobycopenia 61% vs 41% | NA | Nausea 63% vs 29%  Fatigue 57% vs 55%  Hyperglycemia 51% vs 44% | NA |
| MAINTAIN(1)[88] | Ribociclib/Fulvestrant (CDK4/6i/SERD) | Fulvestrant (SERD) | NA | NA | NA | NA |
| MAINTAIN(2)[88] | Ribociclib/Exemestane (CDK4/6i/AI) | Exemestane (AI) | NA | NA | NA | NA |
| PACE(1)  [89,90] | Palbociclib/Fulvestrant (CDK4/6i/SERD) | Fulvestrant (SERD) | NA | NA | NA | NA |
| PACE(2)  [89,90] | Palbociclib/Fulvestrant/Avelumab  (CDK4/6i/SERD/ICI) | Fulvestrant (SERD) | NA | NA | NA | NA |
| SERENA-2(1)  [91] | Camizestrant75mg (SERD) | Fulvestrant (SERD) | NA | NA | NA | NA |
| SERENA-2(2)  [91] | Camizestrant150mg (SERD) | Fulvestrant (SERD) | NA | NA | NA | NA |
| PEARL  [92,93] | Palbociclib/Fulvestrant (CDK4/6i/SERD) | Capecitabine (single-agent chemotherapy) | NA | Neutrophil count decreased 61.1% vs 6.6%  White blood cell decreased 34.9% vs 2.8%  Anaemia 4.0% vs 5.2% | NA | Hand and foot syndrome 0% vs 24.2%  Diarrhoea 1.3% vs 7.6%  Fatigue 0.7% vs 5.5% |

Abbreviations: AI, Aromatase inhibitor; AKTi, AKT inhibitors; Anti-VEGF, Anti-vascular endothelial growth factor; CDK4/6i, Cyclin-dependent kinase 4 and 6 inhibitors; EGFRi: Epidermal Growth Factor Receptor inhibitor; HDACi, Histone deacetylase inhibitor; ICI, Immune checkpoint inhibitor; mTORi, Mammalian target of rapamycin inhibitor; Pi, Protease inhibitor; PI3Ki, Phosphatidylinositol 3‐kinase inhibitor; SERD, Selective Estrogen Receptor Degrader.

Note: References refer to the citation numbers in the main text.

# Table S13 Summary of Hazard ratios for post-line RCTs’ PFS and OS

| Trial information | Intervention arm | Control arm | Second line PFS HR (95% CI) | Second line OS HR  (95% CI) | Third line PFS HR  (95% CI) | Third line OS HR  (95% CI) |
| --- | --- | --- | --- | --- | --- | --- |
| PALOMA-3 [33-36] | Palbociclib/Fulvestrant | Fulvestrant | 0.47 (0.29-0.76) | 0.84 (0.61-1.14) | 0.30 (0.17-0.53) | 0.80 (0.52-1.23) |
| MONALEESA-3 [46, 47] | Ribociclib/Fulvestrant | Fulvestrant | 0.57 (0.43-0.74) | 0.78 (0.59-1.04) | NA | NA |
| MONARCH-2 [48, 49] | Abemaciclib/Fulvestrant | Fulvestrant | 0.48 (0.36-0.64) | 0.66 (0.46-0.94) | NA | NA |
| MONARCH plus B [55, 56] | Abemaciclib/Fulvestrant | Fulvestrant | 0.48 (0.32-0.71) | NA | NA | NA |
| DAWNA-1 [57, 58] | Dalpiciclib/Fulvestrant | Fulvestrant | 0.49 (0.34-0.71) | NA | 0.38 (0.21-0.66) | NA |
| BOLERO-2 [60, 61] | Everolimus/Exemestane | Exemestane | 0.58 (0.29-0.89) | NA | 0.63 (0.41-0.85) | NA |
| PrE0102 [63] | Fulvestrant/Everolimus | Fulvestrant | 0.61 (0.40-0.92) | 1.31 (0.72-2.38) | NA | NA |
| BOLERO-6(1) [64] | Everolimus/Exemestane | Everolimus | 0.74 (0.57-0.97) | 1.27 (0.95-1.70) | NA | NA |
| BOLERO-6(2) [64] | Everolimus/Exemestane | Capecitabine | 1.26 (0.96-1.66) | 1.33 (0.99-1.79) | NA | NA |
| BELLE-3 [68] | Buparlisib/Fulvestrant | Fulvestrant | 0.53 (0.33-0.87) | NA | 0.72 (0.52-0.99) | NA |
| ACE [72] | Tucidinostat/Exemestane | Exemestane | 0.80 (0.58-1.09) | NA | 0.68 (0.43-1.08) | NA |
| FERGI [75] | Pictilisib/Fulvestrant | Fulvestrant | 0.87 (0.53-1.44) | NA | 0.62 (0.36-1.04) | NA |
| EMERALD [79] | Elacestrant | Fulvestrant | 0.71 (0.52-0.96) | NA | 0.60 (0.42-0.84) | NA |
| E2112 [80] | Entinostat/Exemestane | Exemestane | 0.87(0.67-1.13) | 0.99 (0.82-1.21) | NA | NA |
| EFECT [85] | Fulvestrant | Exemestane | 0.91 (0.69,1.13) | NA | NA | NA |
| PEARL [92, 93] | Palbociclib/Fulvestrant | Capecitabine | 1.14 (0.77-1.67) | 1.05 (0.69-1.60) | NA | NA |

Note: References refer to the citation numbers in the main text.

# Table S14 Hazard ratios for second-line OS and PFS, and third-line PFS from Cox-PH model analysis

A: Second-line PFS

| Treatment | Hazard ratio (95% CI) | P-score | Rank | Treatment | Hazard ratio (95% CI) | P-score | Rank |
| --- | --- | --- | --- | --- | --- | --- | --- |
| Eribulin or Gemcitabine or Capecitabine | 0.45 (0.29 ~ 0.71) | 0.8611 | 1 | Elacestrant | 0.71 (0.52 ~ 0.96) | 0.4382 | 9 |
| Abema/FUL | 0.48 (0.38 ~ 0.61) | 0.8212 | 2 | EVE | 0.79 (0.46 ~ 1.33) | 0.3365 | 10 |
| Dalpiciclib/FUL | 0.49 (0.34 ~ 0.71) | 0.7854 | 3 | Tucidinostat/EXE | 0.87 (0.58 ~ 1.29) | 0.2699 | 11 |
| Palbo/FUL | 0.50 (0.33 ~ 0.75) | 0.7723 | 4 | Pictilisib/FUL | 0.87 (0.53 ~ 1.43) | 0.2682 | 12 |
| Buparlisib/FUL | 0.53 (0.33 ~ 0.86) | 0.7053 | 5 | ENT/EXE | 0.94 (0.66 ~ 1.34) | 0.1972 | 13 |
| Ribo/FUL | 0.56 (0.43 ~ 0.74) | 0.6524 | 6 | FUL | - | 0.1333 | 14 |
| EVE/EXE | 0.58 (0.37 ~ 0.92) | 0.6201 | 7 | EXE | 1.08 (0.85 ~ 1.37) | 0.0608 | 15 |
| FUL/EVE | 0.61 (0.40 ~ 0.93) | 0.5781 | 8 |  |  |  |  |

Abbreviations: Abema, Abemaciclib; ENT, Entinostat; EVE, Everolimus; EXE, Exemestane; FUL, Fulvestrant; Palbo, Palbociclib; Ribo, Ribociclib.

B: Second-line OS

| Treatment | Hazard ratio (95% CI) | P-score | Rank | Treatment | Hazard ratio (95% CI) | P-score | Rank |
| --- | --- | --- | --- | --- | --- | --- | --- |
| Abema/FUL | 0.66 (0.46 ~ 0.93) | 0.8493 | 1 | Palbo/FUL | 0.83 (0.61 ~ 1.14) | 0.5760 | 5 |
| Ribo/FUL | 0.78 (0.59 ~ 1.04) | 0.6651 | 2 | FUL | - | 0.2942 | 6 |
| Eribulin or Gemcitabine or Capecitabine | 0.80 (0.47 ~ 1.34) | 0.6559 | 3 | EVE/EXE | 1.06 (0.58 ~ 1.93) | 0.2370 | 7 |
| EVE | 0.83 (0.43 ~ 1.63) | 0.5864 | 4 | FUL/EVE | 1.31 (0.72 ~ 2.38) | 0.1361 | 8 |

Abbreviations: Abema, Abemaciclib; EVE, Everolimus; EXE, Exemestane; FUL, Fulvestrant; Palbo, Palbociclib; Ribo, Ribociclib.

C: Third-line PFS

| Treatment | Hazard ratio (95% CI) | P-score | Rank |
| --- | --- | --- | --- |
| Palbo/FUL | 0.30 (0.17 ~ 0.53) | 0.79275 | 1 |
| Dalpiciclib/FUL | 0.38 (0.19 ~ 0.76) | 0.7967 | 2 |
| Elacestrant | 0.60 (0.42 ~ 0.84) | 0.4953 | 3 |
| Pictilisib/FUL | 0.62 (0.36 ~ 1.05) | 0.4534 | 4 |
| Buparlisib/FUL | 0.72 (0.52 ~ 0.99) | 0.3139 | 5 |
| FUL | - | 0.0133 | 6 |

Abbreviations: FUL, Fulvestrant; Palbo, Palbociclib.

# Table S15 Convergence and heterogeneity assessment

| Network | Cochran’s Q | P-value | Model category | Network | I^2^ | Model category |
| --- | --- | --- | --- | --- | --- | --- |
| first line therapies' PFS | 2.684 | 0.612 | fixed | Any-grade adverse events for first-line mechanisms | 0% | fixed |
| first-line therapies' OS | 0.088 | 0.957 | fixed | Grade 3-5 adverse events for first-line mechanisms | 5% | random |
| first-line mechanisms' PFS | 7.921 | 0.720 | fixed | Adverse events leading to in discontinuation first-line mechanisms | 7% | fixed |
| first-line mechanisms' OS | 7.304 | 0.294 | fixed | Adverse events leading to in death first-line mechanisms | 2% | fixed |
| second line therapies' PFS | 0.086 | 0.769 | fixed | Any-grade adverse events for second-line mechanisms | 25% | random |
| second line therapies' OS | 0 | NA | fixed | Grade 3-5 adverse events for second-line mechanisms | 2% | random |
| second line mechanisms' PFS | 2.483 | 0.115 | fixed | Adverse events leading to in discontinuation second-line mechanisms | 0 | random |
| second line mechanisms' OS | 8.008 | 0.433 | fixed | Adverse events leading to death in second-line mechanisms | 14% | fixed |
| PFS in first-line treatment with visceral metastasis | 2.272 | 0.686 | fixed | Any-grade adverse events for first-line therapies | 11.40% | random |
| PFS of first-line patients over 65 years | 6.805 | 0.178 | fixed | Grade 3-5 adverse events for first-line therapies | 0 | random |
| PFS of first-line patients under 65 years | 4.85 | 0.183 | fixed | Adverse events leading to discontinuation in first-line therapies | 0 | fixed |
| PFS of asian patients in first-line treatment | 1.126 | 0.289 | fixed | Adverse events leading to death in first-line therapies | 11.00% | fixed |
| PFS in second-line treatment with visceral metastasis | 2.907 | 0.192 | fixed | Any-grade adverse events for second-line therapies | 0% | fixed |
| PFS of asian patients in second-line | 0 | NA | fixed | Grade 3-5 adverse events for second-line therapies | 18% | random |
| PFS of second-line ESR1 mutation status patients | 0 | NA | fixed | Adverse events leading to discontinuation in second-line therapies | 6% | fixed |
| PFS of second-line PIK3CA mutation status patients | 4.033 | 0.145 | fixed | Adverse events leading to death in second-line therapies | 13% | random |

# Table S16 Heterogeneity assessment information

| Term | Leverage residual map |
| --- | --- |
| Any-grade adverse events for first-line mechanisms | 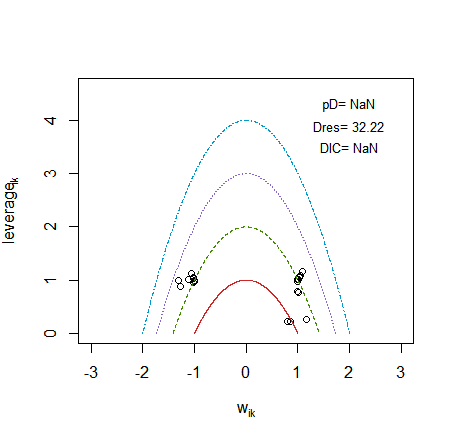 |
| Grade 3-5 adverse events for first-line mechanisms | 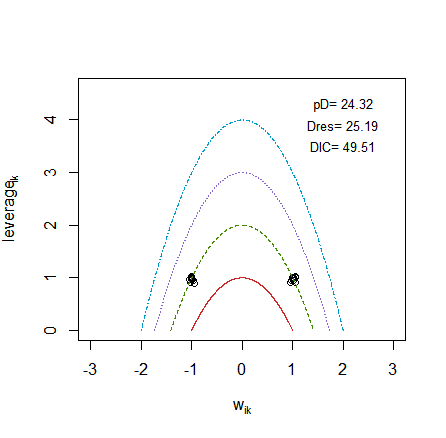 |
| Adverse events leading to in discontinuation first-line mechanisms | 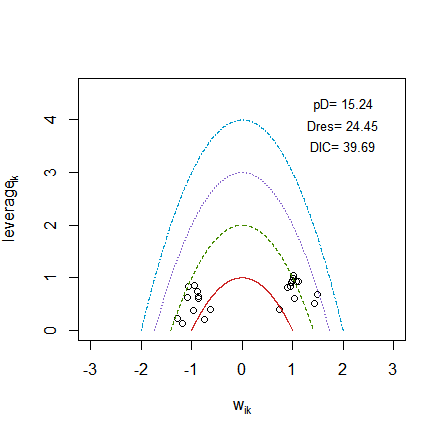 |
| Adverse events leading to in death first-line mechanisms | 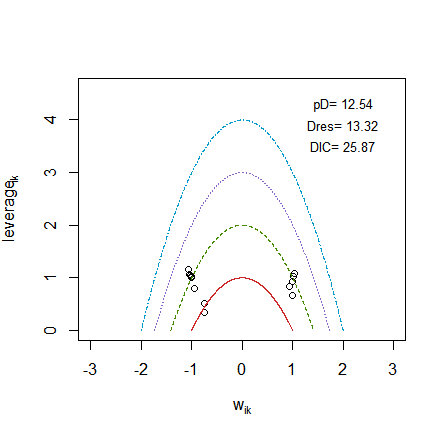 |
| Any-grade adverse events for second-line mechanisms | 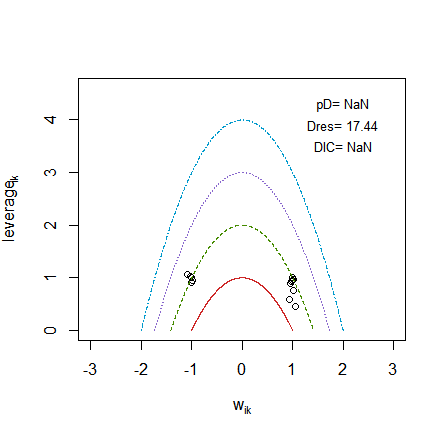 |
| Grade 3-5 adverse events for second-line mechanisms | 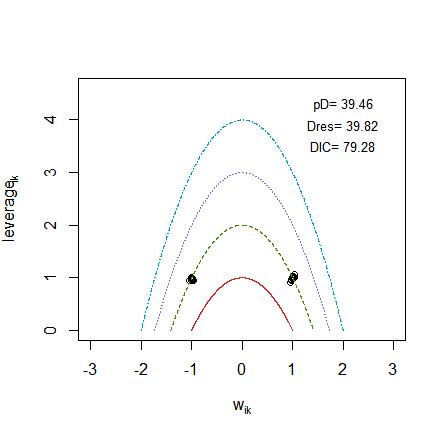 |
| Adverse events leading to in discontinuation second-line mechanisms | 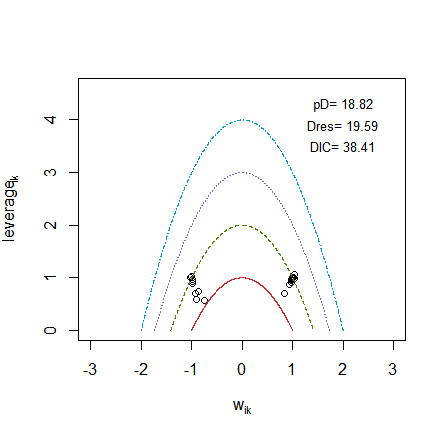 |
| Adverse events leading to death in second-line mechanisms | 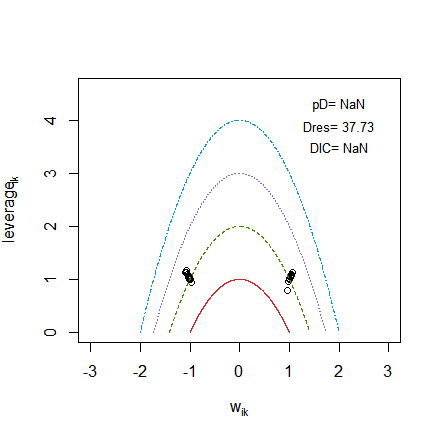 |
| Any-grade adverse events for first-line therapies | 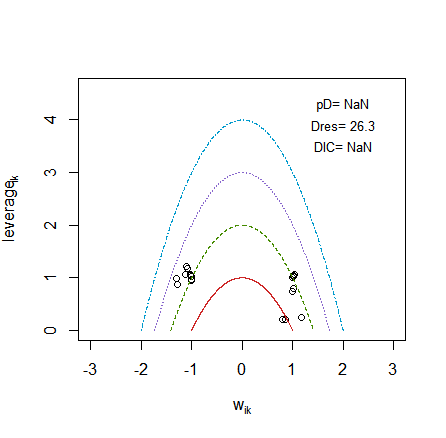 |
| Grade 3-5 adverse events for first-line therapies | 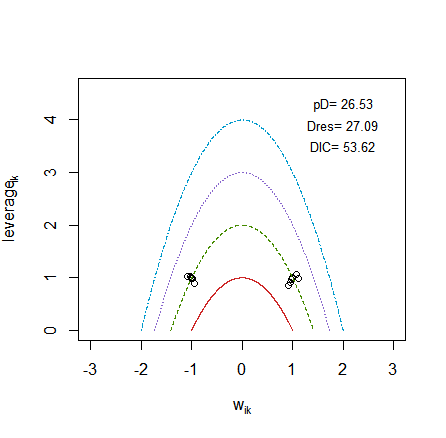 |
| Adverse events leading to discontinuation in first-line therapies | 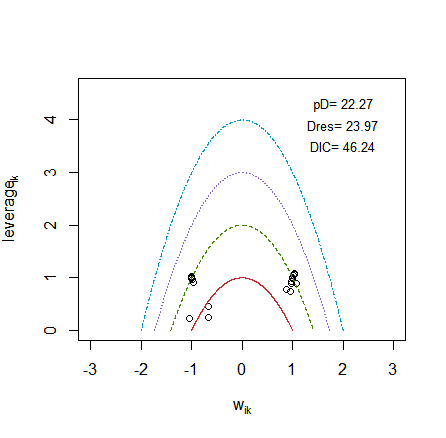 |
| Adverse events leading to death in first-line therapies | 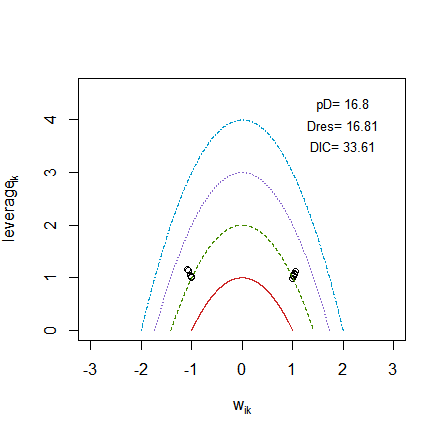 |
| Any-grade adverse events for second-line therapies | 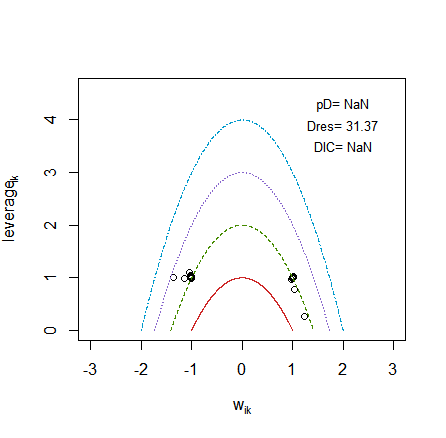 |
| Grade 3-5 adverse events for second-line therapies | 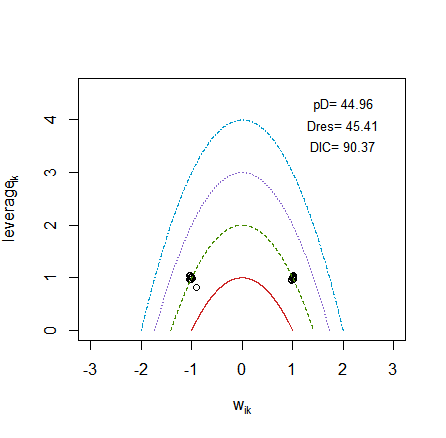 |
| Adverse events leading to discontinuation in second-line therapies | 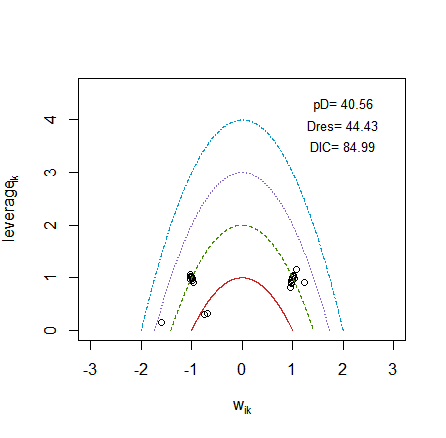 |
| Adverse events leading to death in second-line therapies | 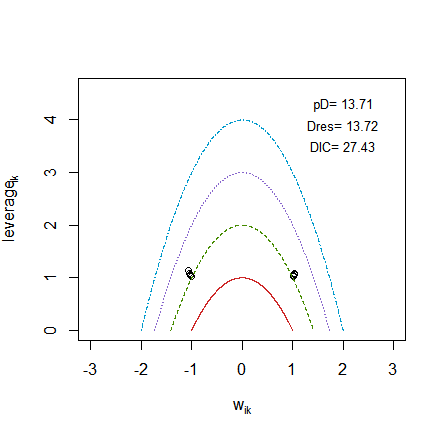 |
