# Supplementary material for: A network meta-analysis of efficacy and safety for first-line and second/further-line therapies in postmenopausal women with hormone receptor-positive, HER2-negative, advanced breast cancer
Source: BMC Med. 2024 Jan 12;22:13. doi: 10.1186/s12916-023-03238-2 (PMC10785354; doi:10.1186/s12916-023-03238-2)
Supplement: Supplementary file 2 — Additional file 2: Figure S1. Methodology quality of the included studies. Figure S2. Funnel plots to detect the publication bias of included studies. Figure S3. Extrapolation results of therapies’ survival curves based on the Fractional Polynomial model. Figure S4. Hazard ratios for PFS and OS from Cox-PH model analysis of first-line mechanisms. Figure S5. Hazard ratios for PFS and OS from Cox-PH model analysis of second/further-lines mechanisms. Figure S6. Subgroup analysis of Cox-PH model results in first-line and second/further-lines therapies. Figure S7. Hazard ratios for PFS and OS from Cox-PH model analysis of FDA-approved first-line therapies. Figure S8. Hazard Ratios for PFS and OS from Cox-PH model analysis of FDA-approved second/further-lines therapies. Figure S9. Transitivity assessment. Figure S10. Sensitivity analysis of patients with full menopause. [file 12916_2023_3238_MOESM2_ESM.docx]

**Additional file 2**

[Figure S1 Methodology quality of the included studies 2](#_Toc14021)

[Figure S2 Funnel plots to detect the publication bias of included studies 3](#_Toc1313)

[Figure S3 Extrapolation results of therapies’ survival curves based on the Fractional Polynomial model 5](#_Toc14574)

[Figure S4 Hazard ratios for PFS and OS from Cox-PH model analysis of first-line mechanisms 8](#_Toc5268)

[Figure S5 Hazard ratios for PFS and OS from Cox-PH model analysis of second/further-lines mechanisms 9](#_Toc24942)

[Figure S6 Subgroup analysis of Cox-PH model results in first-line and second/further-lines therapies 10](#_Toc19632)

[Figure S7 Hazard ratios for PFS and OS from Cox-PH model analysis of FDA-approved first-line therapies 11](#_Toc489)

[Figure S8 Hazard Ratios for PFS and OS from Cox-PH model analysis of FDA-approved second/further-lines therapies 13](#_Toc30705)

[Figure S9 Transitivity assessment 15](#_Toc9391)

[Figure S10 Sensitivity analysis of patients with full menopause 18](#_Toc16932)

# Figure S1 Methodology quality of the included studies


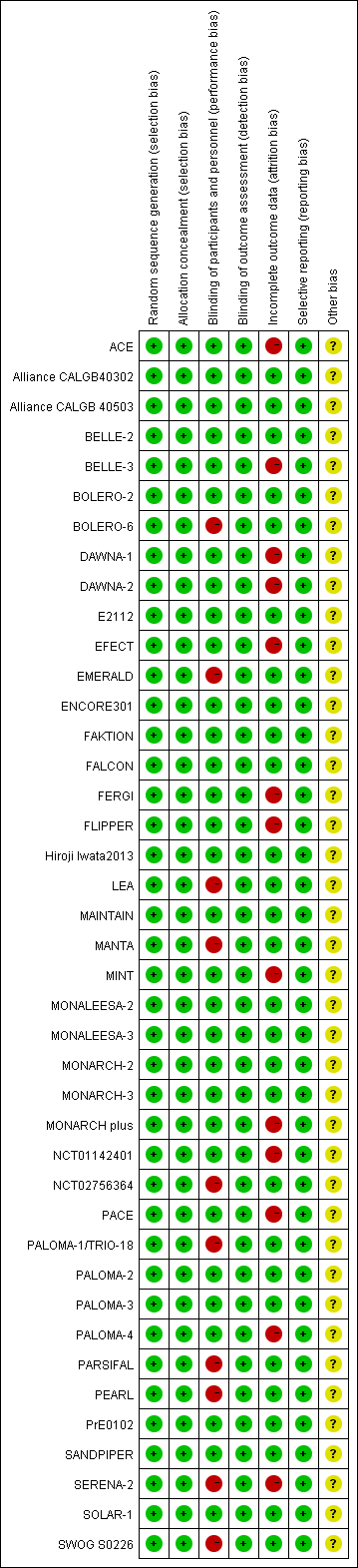


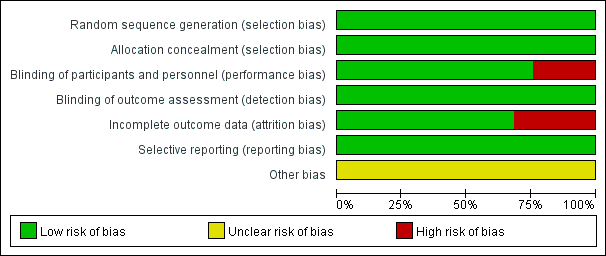


# Figure S2 Funnel plots to detect the publication bias of included studies

| A |
| --- |
| 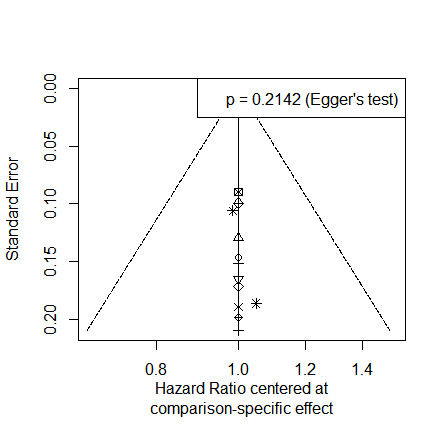 |
| B |
| 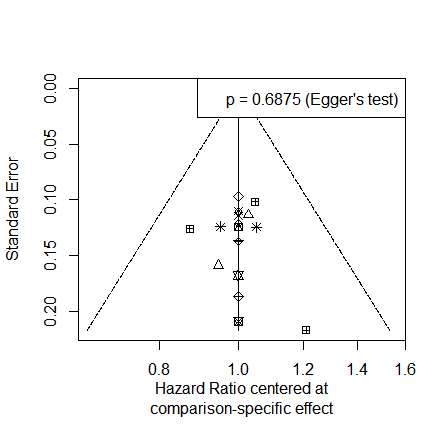 |
| C |
| 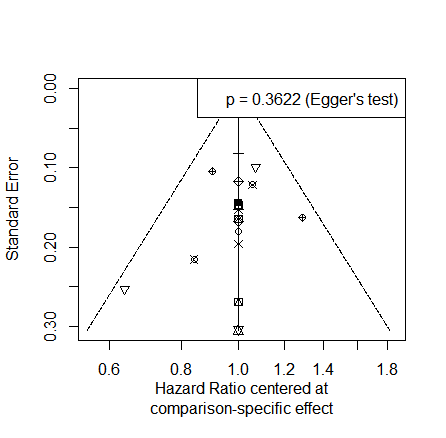 |
| D |
| 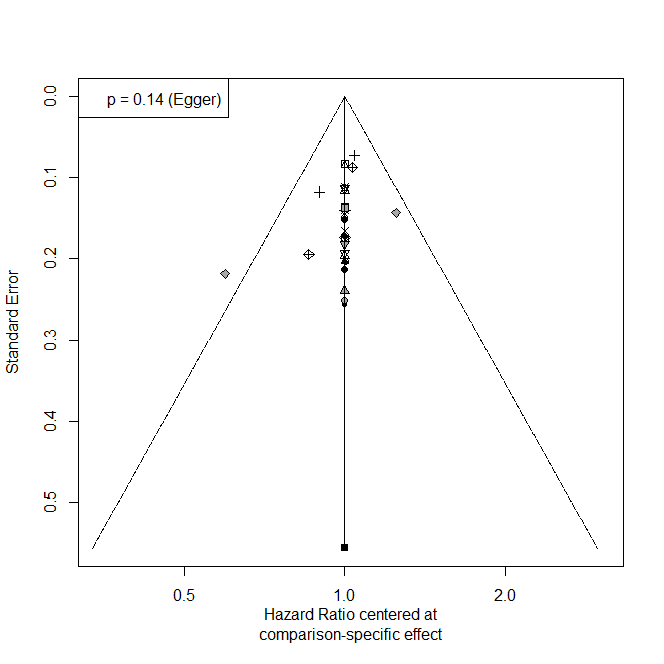 |

Note: A: Funnel plots in network for first-line OS; B: Funnel plots in network for first-line PFS; C: Funnel plots in network for second/further-lines OS; D: Funnel plots in network for second/further-lines PFS

# Figure S3 Extrapolation results of therapies’ survival curves based on the Fractional Polynomial model

| A |
| --- |
| 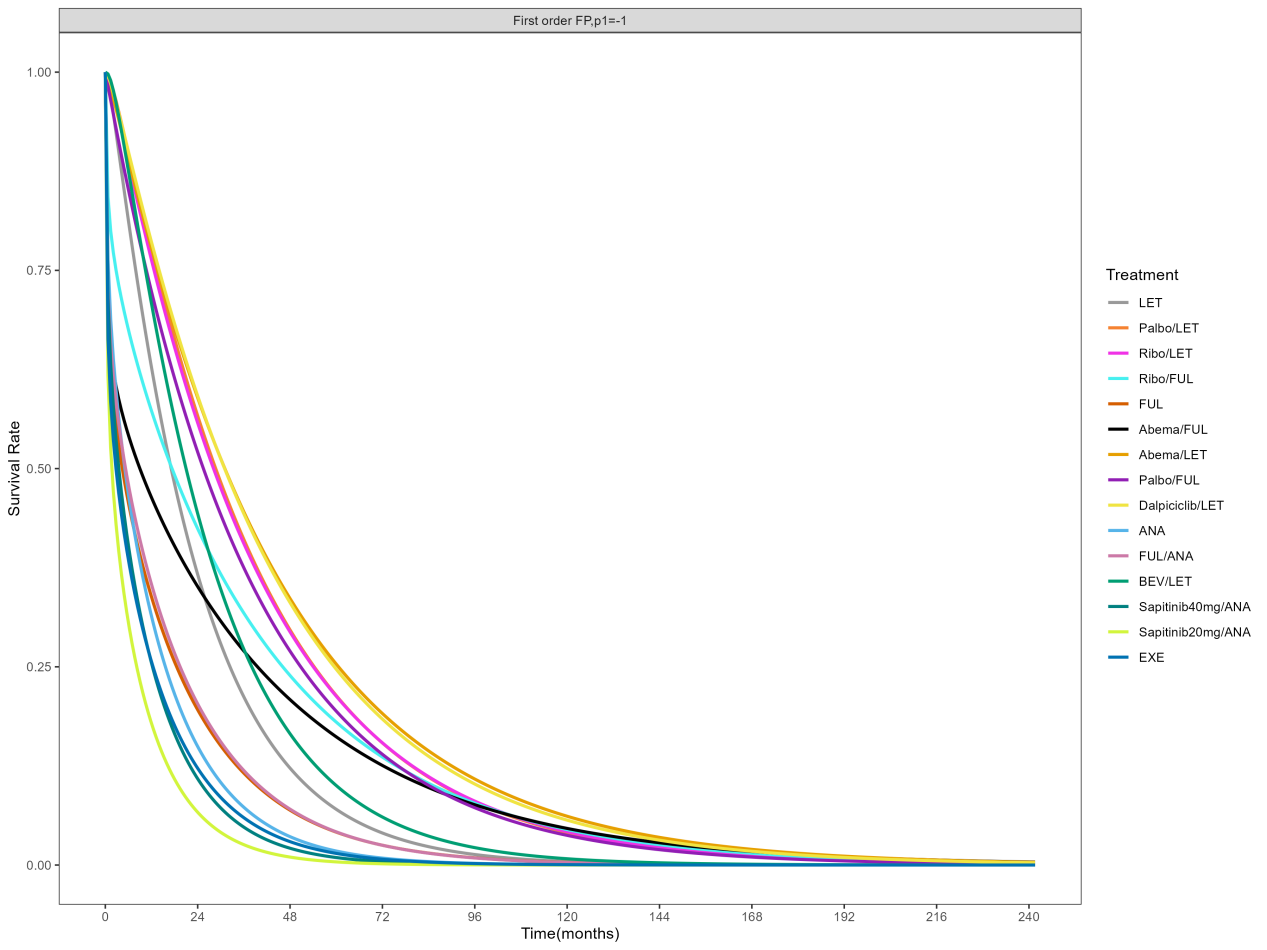 |
| B |
| 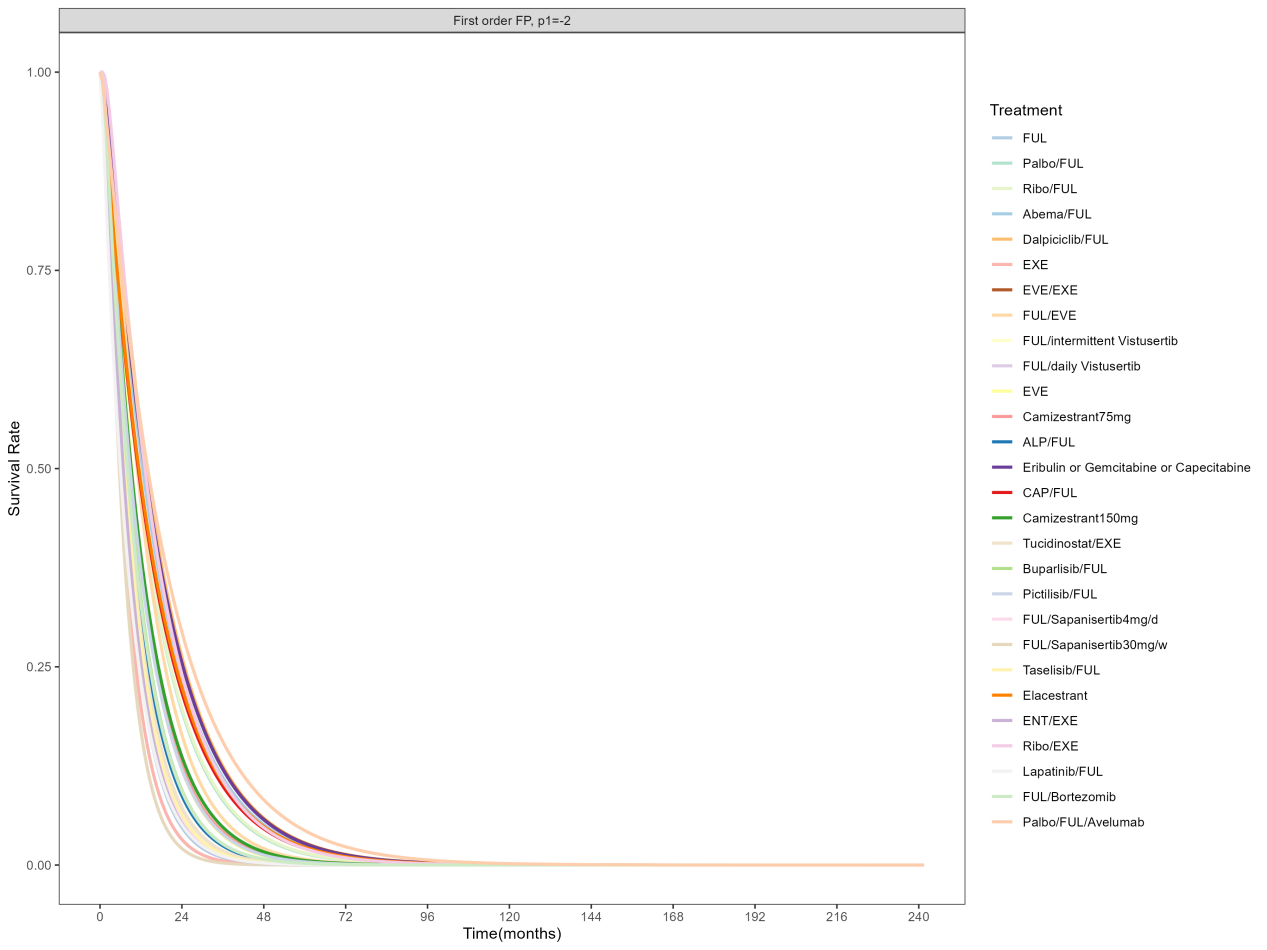 |
| C |
| 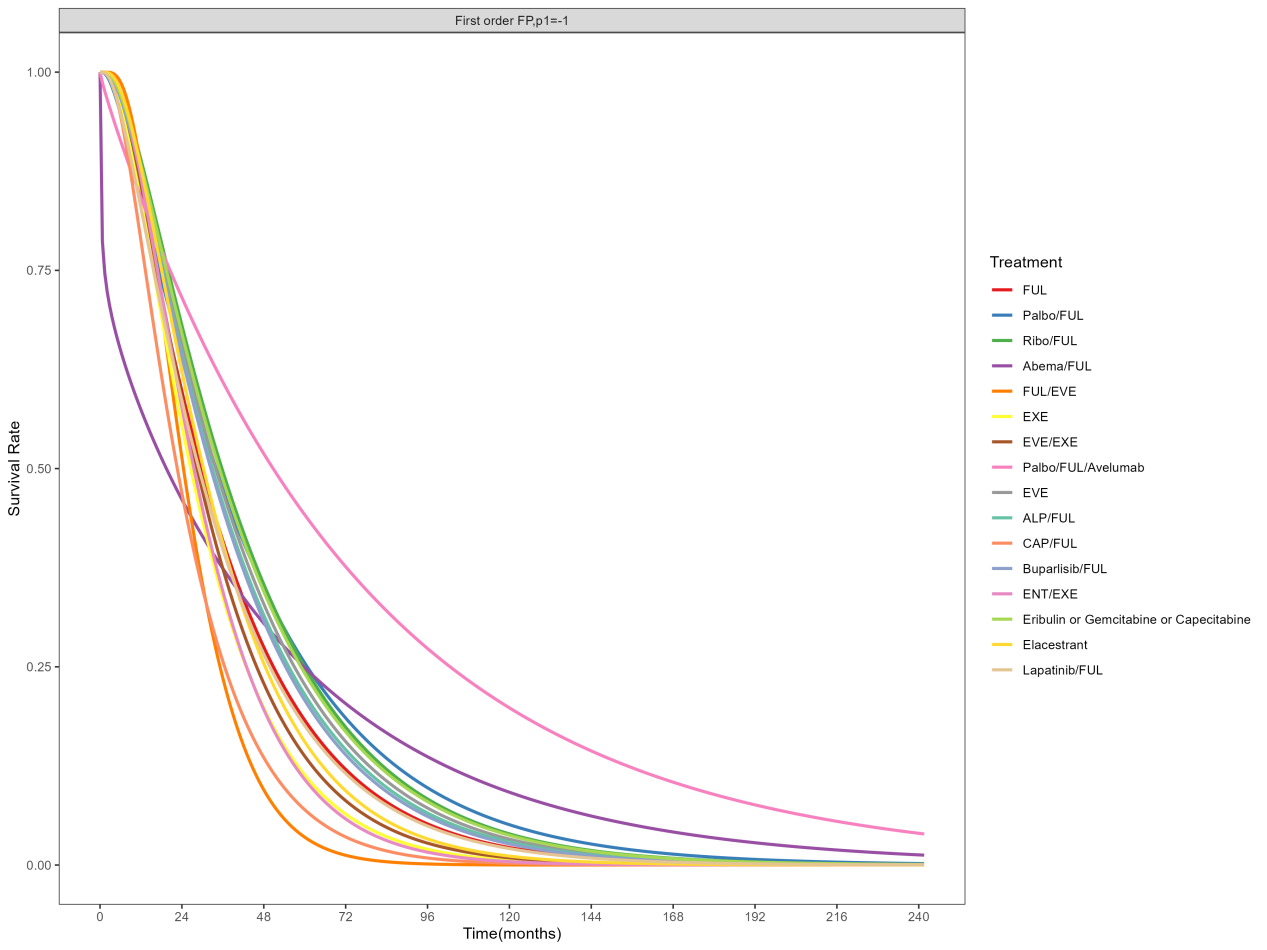 |

Note: A: First-line therapies’ PFS survival curves; B: Second/further-lines therapies’ PFS survival curves; C: Second/further-lines therapies’ OS survival curves.

Abbreviations: Abema, Abemaciclib; ALP, Alpelisib; ANA, Anastrozole; BEV, Bevacizumab; CAP, Capivasertib; ENT, Entinostat; EXE, Exemestane; EVE, Everolimus; FUL, Fulvestrant; Palbo, Palbociclib; Ribo, Ribociclib.

# Figure S4 Hazard ratios for PFS and OS from Cox-PH model analysis of first-line mechanisms


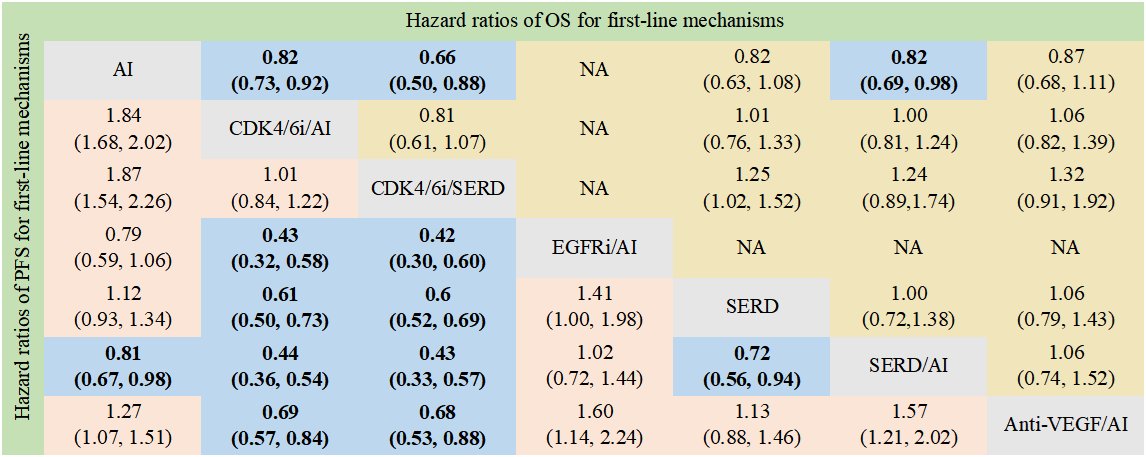


Note: Hazard ratios (HRs) for progression-free survival (PFS, lower triangle) and HRs for overall survival (OS, upper triangle) with their 95% credible intervals (95% CIs) derived from network meta-analysis of first-line mechanisms

The direction of the reported relative effects in each cell is defined as treatment on the right vs. treatment on the left. Values < 1 favor the intervention on the right. Values in parenthesis are 95% credible intervals (95% CIs). Bold cells correspond to statistically significant relative effects for the respective treatment categories.

Abbreviations: AI, Aromatase Inhibitor; Anti-VEGF, Anti-Vascular Endothelial Growth Factor; CDK4/6i, Cyclin-Dependent Kinase 4 and 6 inhibitor; EGFRi: Epidermal Growth Factor Receptor inhibitor; NA, Not Available; SERD, Selective Estrogen Receptor Degrader.

# Figure S5 Hazard ratios for PFS and OS from Cox-PH model analysis of second/further-lines mechanisms

Note: Hazard ratios (HRs) for progression-free survival (PFS, lower triangle) and HRs for overall survival (OS, upper triangle) with their 95% credible intervals (95% CIs) derived from network meta-analysis of second-line mechanisms

The direction of the reported relative effects in each cell is defined as treatment on the right vs. treatment on the left. Values < 1 favor the intervention on the right. Values in parenthesis are 95% credible intervals (95% CIs). Bold cells correspond to statistically significant relative effects for the respective treatment categories.

Abbreviations: AI, Aromatase inhibitor; AKTi, AKT inhibitor; Anti-VEGF, Anti-vascular endothelial growth factor; CDK4/6i, cyclin-dependent kinase 4 and 6 inhibitor; EGFRi, Epidermal Growth Factor Receptor inhibitor; HDACi, Histone deacetylase inhibitor; ICI, Immune checkpoint inhibitors; mTORi, mammalian target of rapamycin inhibitor; Pi, protease inhibitor; PI3Ki, phosphatidylinositol 3‐kinase inhibitor; SERD, Selective Estrogen Receptor Degrader.

# Figure S6 Subgroup analysis of Cox-PH model results in first-line and second/further-lines therapies


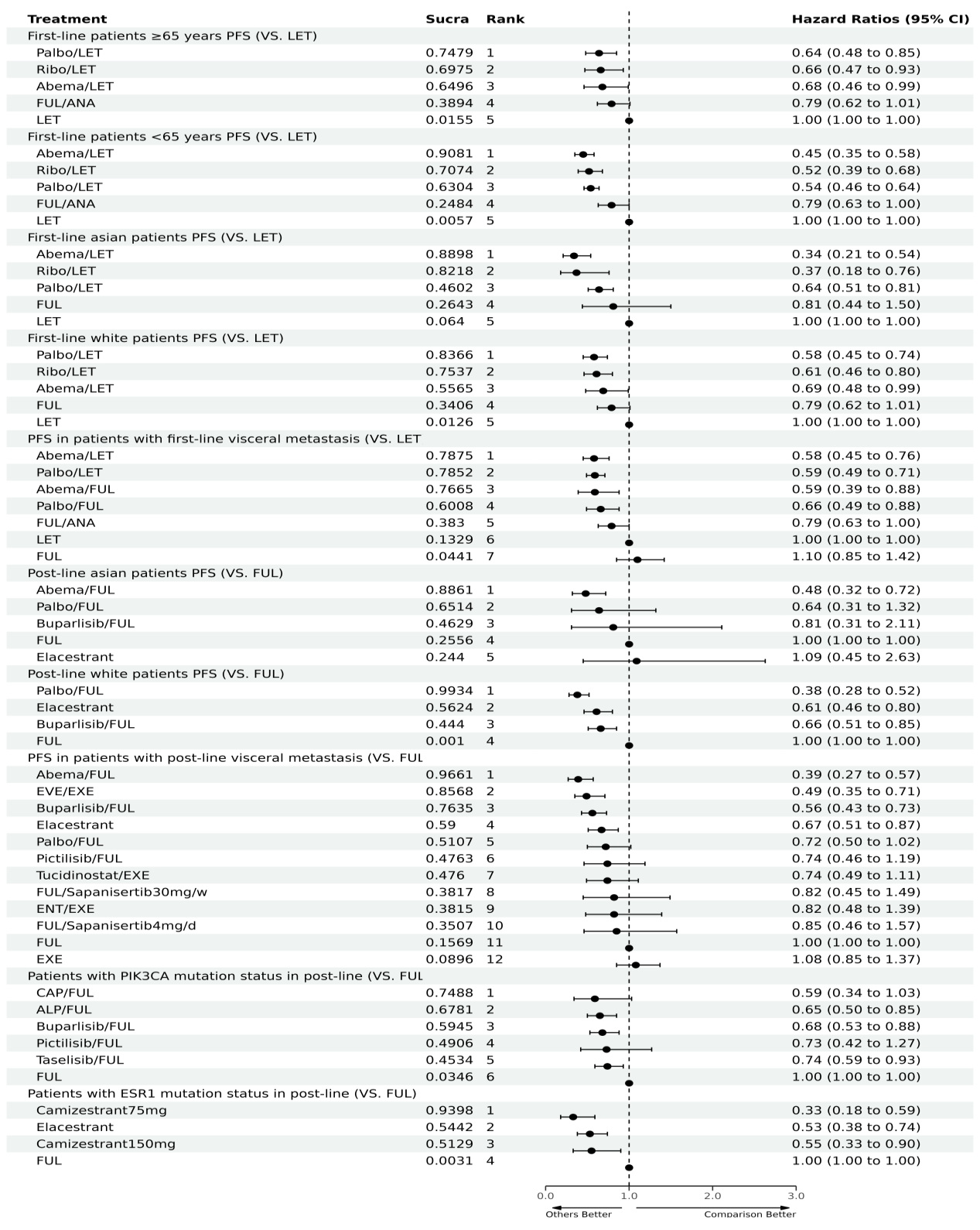


Abbreviations: Abema, Abemaciclib; ALP, Alpelisib; ANA: Anastrozole; CAP, Capivasertib; ENT, Entinostat; EVE, Everolimus; EXE, Exemestane; ESR1, Estrogen Receptor 1; FUL, Fulvestrant; LET, Letrozole; Palbo, Palbociclib; PIK3CA, Phosphatidylinositol-4,5-Bisphosphate 3-Kinase Catalytic Subunit Alpha; Ribo, Ribociclib.

# Figure S7 Hazard ratios for PFS and OS from Cox-PH model analysis of FDA-approved first-line therapies


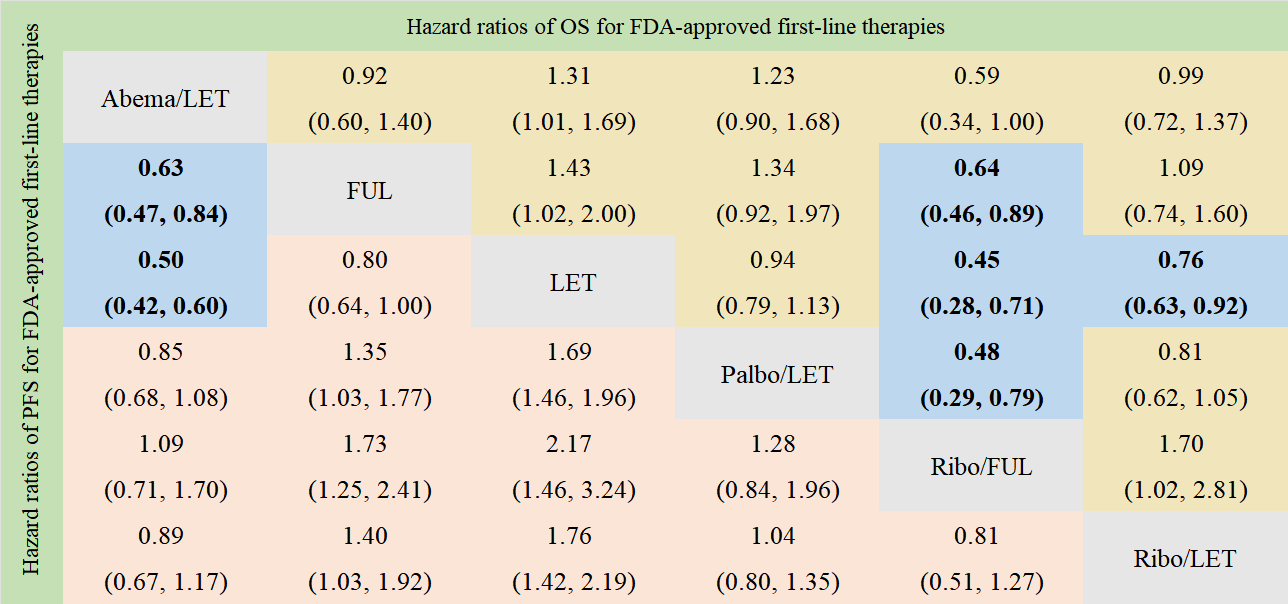


Note: Hazard ratios (HRs) for progression-free survival (PFS, lower triangle) and HRs for overall survival (OS, upper triangle) with their 95% credible intervals (95% CIs) derived from network meta-analysis of FDA-approved first-line therapies

The direction of the reported relative effects in each cell is defined as treatment on the right vs. treatment on the left. Values < 1 favor the intervention on the right. Values in parenthesis are 95% credible intervals (95% CIs). Bold cells correspond to statistically significant relative effects for the respective treatment categories.

Abbreviations: Abema, Abemaciclib; FUL, Fulvestrant; LET, Letrozole; Palbo, Palbociclib; Ribo, Ribociclib.

# Figure S8 Hazard Ratios for PFS and OS from Cox-PH model analysis of FDA-approved second/further-lines therapies

Note: Hazard ratios (HRs) for progression-free survival (PFS, lower triangle) and HRs for overall survival (OS, upper triangle) with their 95% credible intervals (95% CIs) derived from network meta-analysis of FDA-approved second-line therapies

The direction of the reported relative effects in each cell is defined as treatment on the right vs. treatment on the left. Values < 1 favor the intervention on the right. Values in parenthesis are 95% credible intervals (95% CIs). Bold cells correspond to statistically significant relative effects for the respective treatment categories.

Abbreviations: Abema, Abemaciclib; ALP, Alpelisib; ENT, Entinostat; EVE, Everolimus; EXE, Exemestane; FUL, Fulvestrant; LET, Letrozole; Palbo, Palbociclib; Ribo, Ribociclib.

# Figure S9 Transitivity assessment

| A |
| --- |
|  |
| B |
|  |
| C |
|  |

Note: A: Transitivity assessment of patients’ age; B: Transitivity assessment of the proportion of patients with an ECOG performance status of 0/1; C: Transitivity assessment of the proportion of white race patients

# Figure S10 Sensitivity analysis of patients with full menopause

| A | B |
| --- | --- |
| 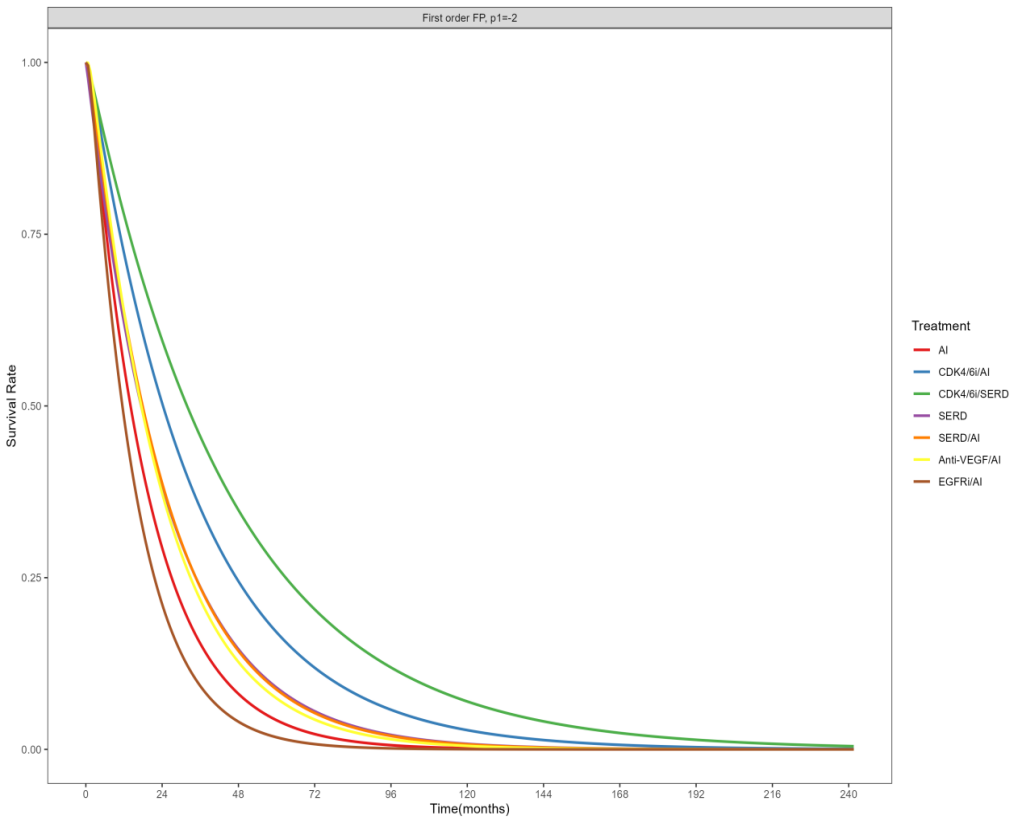 | 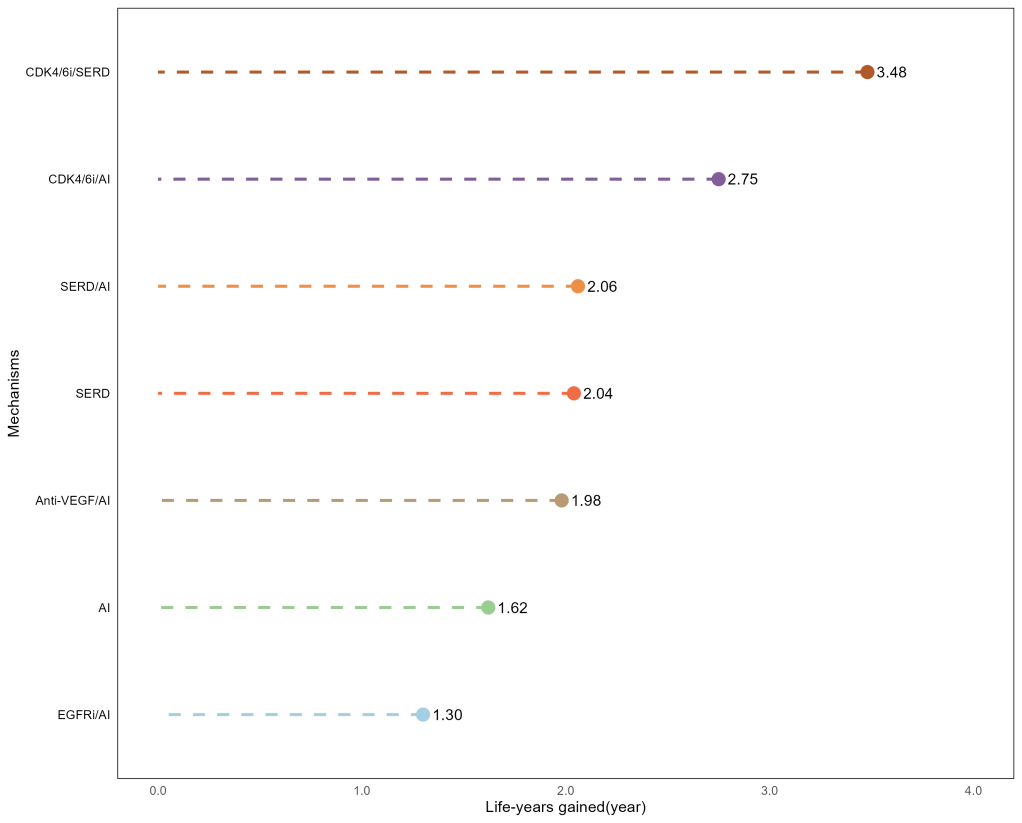 |
| C | D |

| 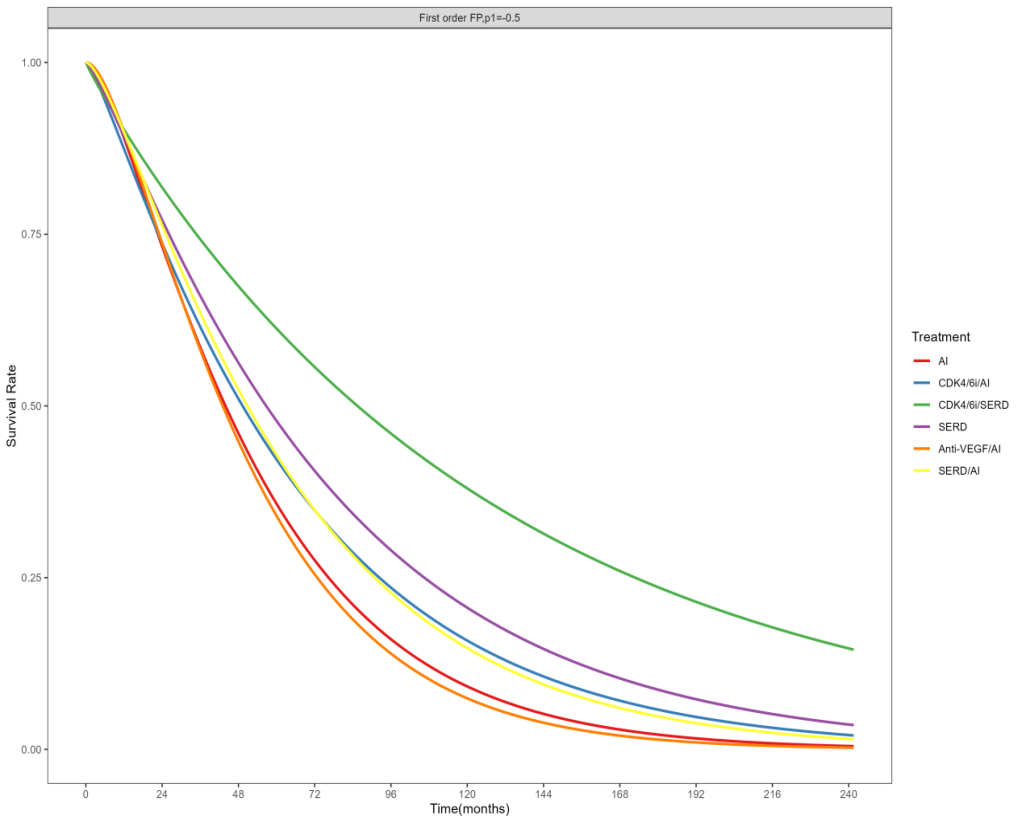 | 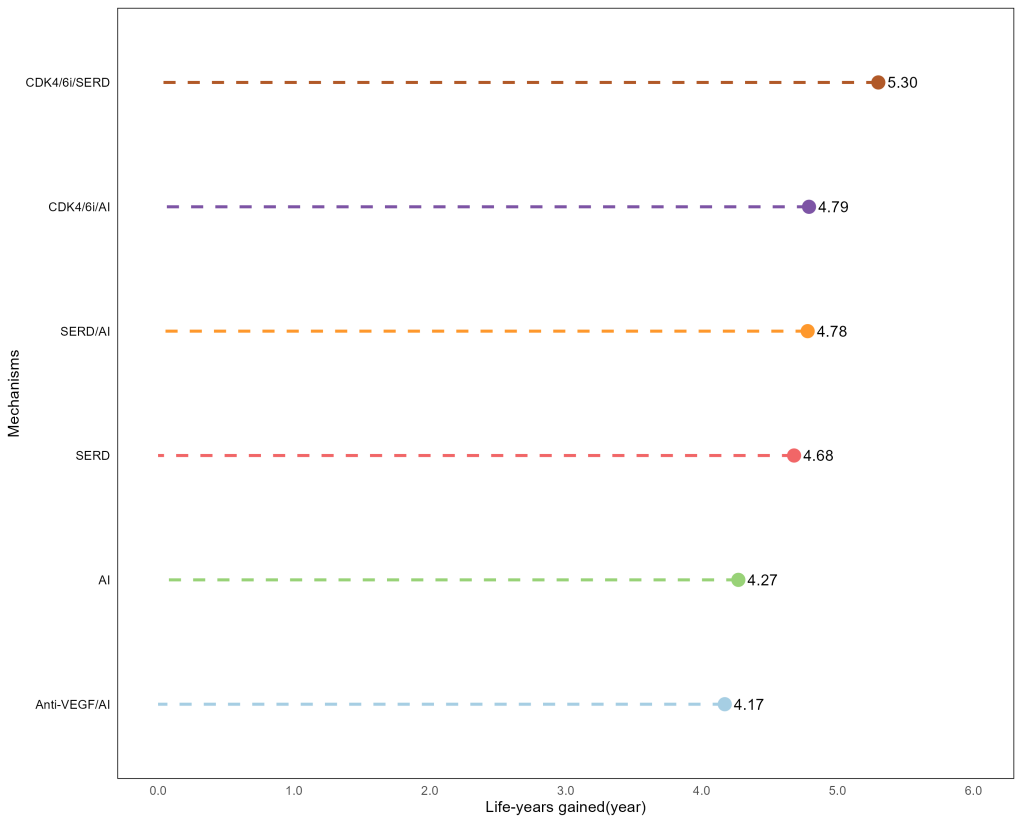 |
| --- | --- |
| E | F |
| 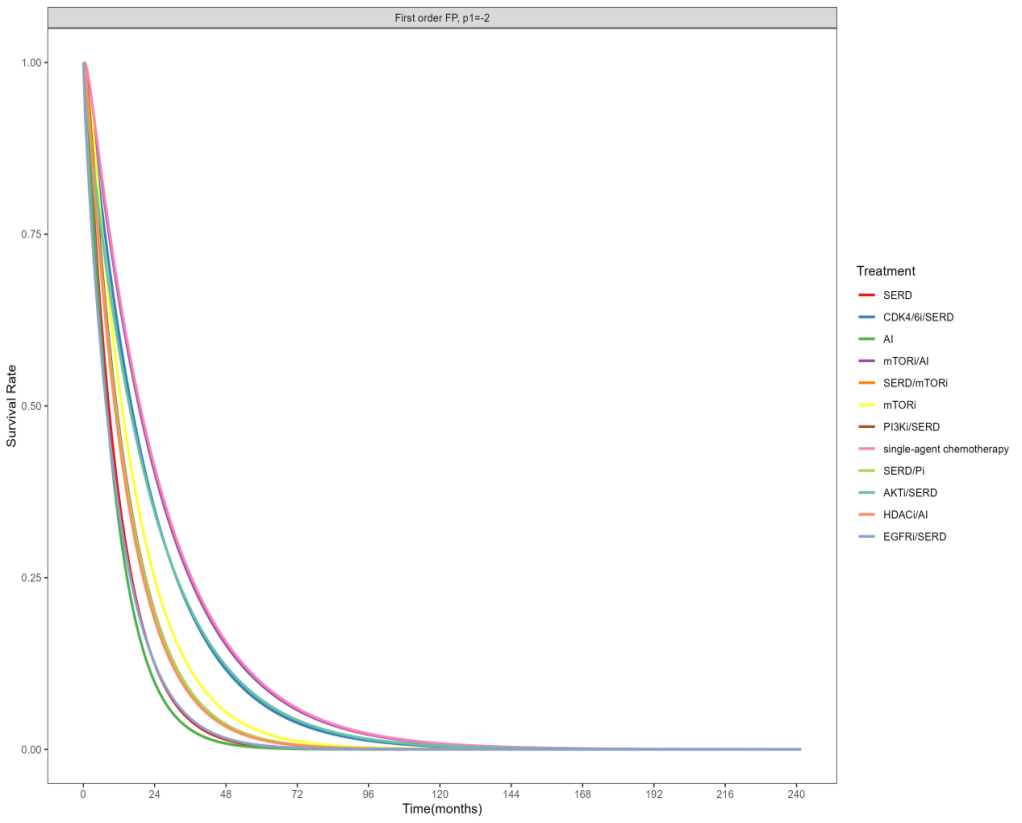 | 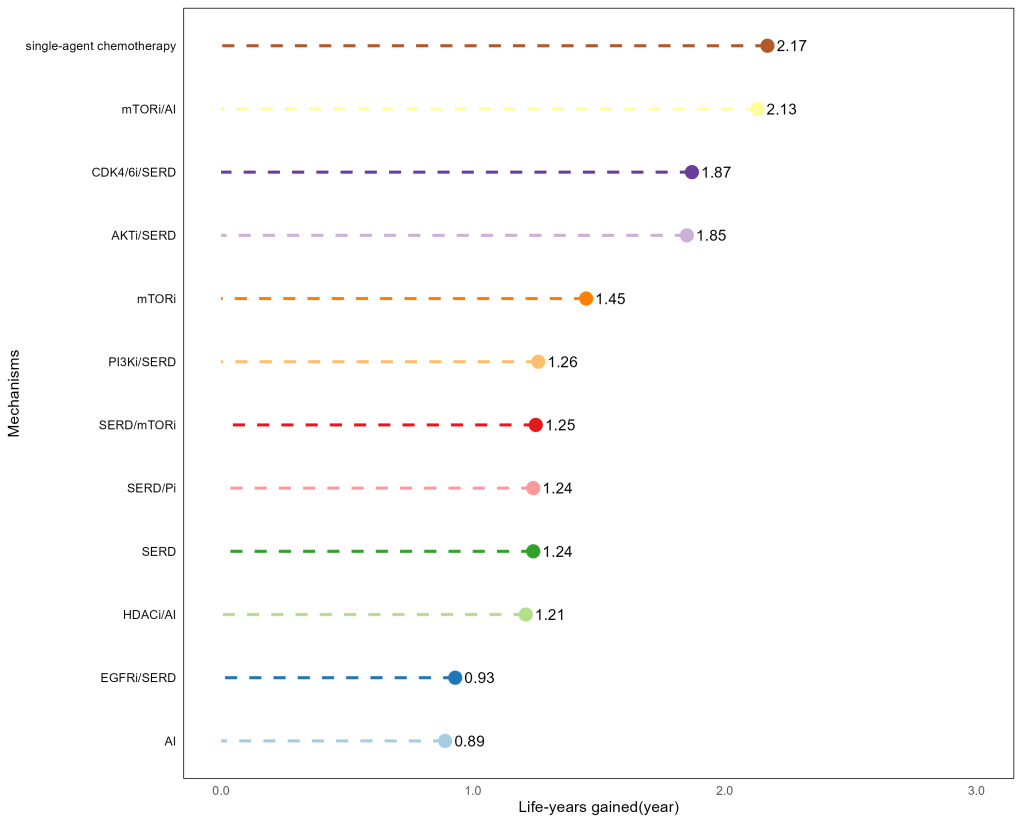 |
| G | H |
| 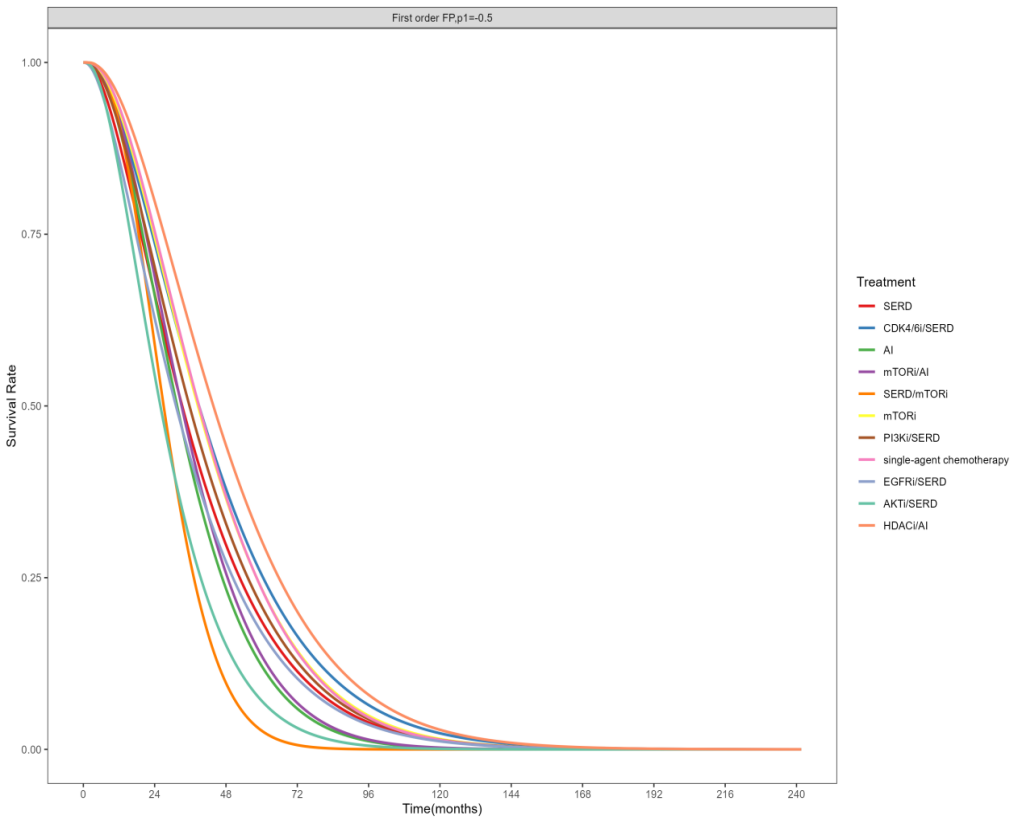 | 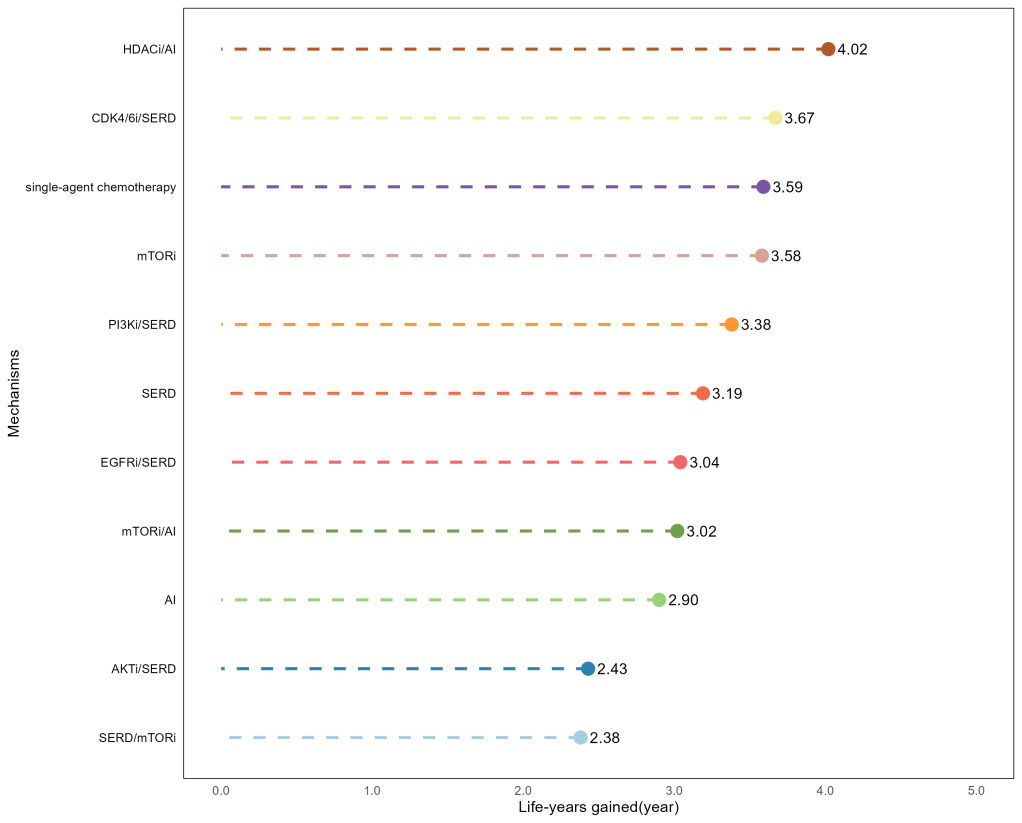 |

Note: Life-years results within 10 years for progression-free survival and overall survival of the first-line and second/further-lines mechanisms (A: Extrapolation results of first-line mechanisms’ PFS survival curves based on the Fractional polynomial model; B: Fractional polynomial model result for first-line mechanisms’ PFS; C: Extrapolation results of first-line mechanisms’ OS survival curves based on the Fractional polynomial model; D: Fractional polynomial model result for first-line mechanisms' OS; E: Extrapolation results of second/further-lines mechanisms’ PFS survival curves based on the Fractional polynomial model; F: Fractional polynomial model result for second/further-lines mechanisms' PFS; G: Extrapolation results of second/further-lines mechanisms’ OS survival curves based on the Fractional polynomial model; H: Fractional polynomial model result for second/further-lines mechanisms' OS)

Abbreviations: AI, Aromatase inhibitor; AKTi, AKT inhibitors; Anti-VEGF, Anti-vascular endothelial growth factor; CDK4/6i, Cyclin-dependent kinase 4 and 6 inhibitors; EGFRi: Epidermal Growth Factor Receptor inhibitor; HDACi, Histone deacetylase inhibitor; mTORi, Mammalian target of rapamycin inhibitor; Pi, protease inhibitor; PI3Ki, Phosphatidylinositol 3‐kinase inhibitor; SERD, Selective Estrogen Receptor Degrader
